# A subset of CD163^+^ macrophages displays mixed polarizations in discoid lupus skin

**DOI:** 10.1186/s13075-015-0839-3

**Published:** 2015-11-13

**Authors:** Benjamin F. Chong, Lin-chiang Tseng, Gregory A. Hosler, Noelle M. Teske, Song Zhang, David R. Karp, Nancy J. Olsen, Chandra Mohan

**Affiliations:** Department of Dermatology, University of Texas Southwestern Medical Center, 5323 Harry Hines Boulevard, Dallas, TX 75390-9069 USA; ProPath, 1355 River Bend Drive, Dallas, 75247 TX USA; Department of Clinical Sciences, University of Texas Southwestern Medical Center, 5323 Harry Hines Blvd., Dallas, 75390 TX USA; Department of Internal Medicine, Rheumatic Diseases Division, University of Texas Southwestern Medical Center, 5323 Harry Hines Blvd., Dallas, 75390-8884 TX USA; Department of Internal Medicine, Division of Rheumatology, Penn State Milton S. Hershey Medical Center, Mail Code H038, Hershey, 17033-0850 PA USA; Department of Biomedical Engineering, University of Houston, 500 University Drive, P.O. Box 850 3065 Cullen Street, Houston, 77204 TX USA

**Keywords:** Discoid lupus erythematosus, Macrophage, Transcriptome, Gene expression

## Abstract

**Introduction:**

Lesional skin of patients with discoid lupus erythematosus (DLE) contains macrophages, whose polarization has yet to be investigated. To test our hypothesis that M1 macrophages would be increased in DLE skin, we examined transcriptome alterations in immune cell gene expression and macrophage features in DLE and normal skin by using gene expression and histochemical approaches.

**Methods:**

Gene expression of RNA from DLE lesional and normal control skin was compared by microarrays and quantitative real-time polymerase chain reaction (RT-PCR). Both skin groups were analyzed for CD163 expression by immunohistochemistry. Double immunofluorescence studies were performed to characterize protein expression of CD163^+^ macrophages.

**Results:**

DLE skin had twice as many upregulated genes than downregulated genes compared with normal skin. Gene set enrichment analysis comparing differentially expressed genes in DLE and normal skin with previously published gene sets associated with M1 and M2 macrophages showed strong overlap between upregulated genes in DLE skin and M1 macrophages. Quantitative RT-PCR showed that several M1 macrophage-associated genes—e.g., chemokine (C-X-C motif) ligand 10 (CXCL10), chemokine (C-C motif) ligand 5 (CCL5), and signal transducer and activator of transcription 1 (STAT1)—had amplified mRNA levels in DLE skin. CD163^+^ macrophages were increased near the epidermal-dermal junction and perivascular areas in DLE skin compared with normal skin. However, double immunofluorescence studies of CD163^+^ macrophages revealed minor co-expression of M1 (CXCL10, tumor necrosis factor-alpha, and CD127) and M2 (CD209 and transforming growth factor-beta) macrophage-related proteins in DLE skin.

**Conclusion:**

Whereas a subset of CD163^+^ macrophages displays mixed polarizations in DLE skin, other immune cells such as T cells can contribute to the expression of these macrophage-related genes.

**Electronic supplementary material:**

The online version of this article (doi:10.1186/s13075-015-0839-3) contains supplementary material, which is available to authorized users.

## Introduction

Discoid lupus erythematosus (DLE), the most common chronic cutaneous lupus subtype [1, 2], is a photosensitive and disfiguring skin disease marked by erythematous scaly papules that transition into hyper- and hypopigmented scarring plaques located most commonly on the face, scalp, and neck [3, 4]. Ten to twenty percent of patients with systemic lupus erythematosus (SLE) have diagnosed DLE [5–7], whereas up to 17 % of patients presenting with an initial diagnosis of DLE develop SLE [1, 8, 9]. Moreover, in light of this overlap, recent studies have shown that multiple gene variations are shared by patients with SLE and patients with DLE [10, 11].

Much attention has been directed toward understanding how T and B cells are involved in cutaneous lupus. Although macrophages are the second most common inflammatory cells in DLE skin (next to T cells) [12–14], they are a relatively understudied population in DLE. Macrophages can direct T-cell differentiation and facilitate antigen presentation, thus influencing immune responses in DLE. Macrophages can be differentially activated into M1 and M2 subtypes, which have contrasting functions. M1 macrophages (classically activated macrophages) assume the traditional roles of phagocytes, which include targeting and clearing microbes, and depict type I immune responses. They produce pro-inflammatory reactive oxygen species and cytokines such as interleukin-12 (IL-12). M2 macrophages (alternatively activated macrophages) counteract type I immune responses with the induction of type II immune responses via secretion of IL-10, resulting in resolution of inflammation. Blood in patients with SLE demonstrated upregulation of M1 macrophage proteins such as chemokine (C-X-C motif) ligand 10 (CXCL10) [15] and downregulation of M2 macrophage proteins, including transforming growth factor-beta (TGF-β) [16]. However, whether this bias carries over into target tissues of patients with lupus, such as skin, is unknown.

Our objective in this study was to investigate changes in immune cell signatures in the transcriptomes of DLE lesional skin versus normal skin by microarray analysis and quantitative real-time polymerase chain reaction (qRT-PCR). Protein expression was assessed by immunohistochemistry and double immunofluorescence. Because lesional skin from patients with inflammatory skin diseases such as psoriasis [17] has been characterized by a distinctly greater presence of classically activated macrophages, we hypothesized that, compared with normal skin, DLE lesional skin would also exhibit a strong M1 macrophage polarization.

## Methods

### Patient recruitment and sample/data collection

Subjects with DLE, diagnosed by clinicopathological correlation, were recruited from the outpatient dermatology clinics at Parkland Health and Hospital System and University of Texas Southwestern (UTSW) Medical Center. Normal controls were additionally recruited from elective plastic surgery cases performed at UTSW Medical Center. DLE patients having individual lesions with a Cutaneous Lupus Activity and Severity Index (CLASI) activity score of at least 2 were included. DLE patients who had SLE, based on fulfillment of at least four American College of Rheumatology (ACR) diagnostic criteria [18] or a history of drug-induced DLE, and normal subjects with a personal history of autoimmune disease were excluded. On the basis of these screening criteria, 17 patients with DLE and 12 normal controls gave informed consent and were enrolled in the study. Medical, family, and medication histories were obtained, and clinical assessments (e.g., CLASI and Systemic Lupus Erythematosus Disease Activity Index, or SLEDAI) performed at the study visit were collected. Patient characteristics are summarized in Table 1, which is subdivided by disease group (normal and DLE) and experiments (microarray and qRT-PCR). DLE subjects underwent one 4- or 6-mm punch biopsy from active inflammatory borders of discoid lesions with individual CLASI activity scores of at least 2. Normal sun-exposed non-lesional skin was obtained as discarded specimens from cutaneous surgeries or elective plastic surgeries. Skin biopsies were placed in RNALater Solution (Ambion, Austin, TX, USA), kept at room temperature overnight, and stored at −80 °C for RNA analysis or in OCT and frozen in −80 °C for immunofluorescence experiments. This study was approved by the University of Texas Southwestern Institutional Review Board and complied with Declaration of Helsinki.

**Table 1:** Subject demographic data

	Normal (microarray)	DLE (microarray)	Normal (qRT-PCR)	DLE (qRT-PCR)
Number	8	9	12	17
Age at visit in years, median (range)	47.0 (40.9–54.0)	40.0 (21.5–56.5)	48.0 (39.3–66.4)	40.2 (21.5–59.9)
Gender, male/female	1/7	1/8	3/9	4/13
Ethnicity, N (%)				
African-American	0 (0)	7 (77.8)	0 (0)	12 (70.6)
Caucasian	8 (100)	0 (0)	12 (100)	4 (23.5)
Hispanic	0 (0)	2 (22.2)	0 (0)	1 (11.1)
CLASI activity score, median (range)	N/A	6 (2–20)	N/A	7 (2–21)
CLASI damage score, median (range)	N/A	14 (6–20)	N/A	10 (0–20)
SLEDAI score, median (range)	N/A	0 (0–0)	N/A	0 (0–4)
Disease duration in years, median (range)^a^	N/A	2.8 (0–16.3)	N/A	1.5 (0–16.3)
Lupus medications, N (%)				
Topical/intralesional corticosteroids/topical immunomodulators	N/A	5 (55.6)	N/A	12 (70.6)
Hydroxychloroquine	N/A	4 (44.4)	N/A	8 (47.0)
Quinacrine	N/A	0 (0)	N/A	1 (5.9)
Methotrexate	N/A	0 (0)	N/A	1 (5.9)
None	N/A	2 (22.2)	N/A	4 (23.5)
ACR SLE criteria, N (%)^b^				
Discoid rash	N/A	9 (100)	N/A	17 (100)
Photosensitivity	N/A	7 (77.8)	N/A	12 (70.6)
Oral ulcers	N/A	2 (22.2)	N/A	3 (17.6)
Hematological disorder	N/A	1 (11.1)	N/A	1 (11.1)
Anti-nuclear antibody^c^	N/A	3 (33.3)	N/A	3 (17.6)
Number of ACR SLE criteria, median (range)	N/A	3 (1–3)	N/A	2 (1–3)

Abbreviations: *ACR* American College of Rheumatology, *CLASI* cutaneous lupus disease area and severity index, *DLE* discoid lupus erythematosus, *N/A* not applicable, *qRT-PCR* quantitative real-time polymerase chain reaction, *SLE* systemic lupus erythematosus, *SLEDAI* systemic lupus erythematosus disease and activity index

^a^Disease duration was not available for two patients with DLE

^b^No patients with DLE had malar rash, arthritis, serositis, renal disorder, neurologic disorder, or immunological disorder

^c^Positive anti-nuclear antibody (ANA) test was determined by history of ANA titers of at least 1:160, as determined by indirect immunofluorescence, or positive enzyme-linked immunosorbent assay

### Microarray analysis

RNA from nine DLE lesional and eight normal skin sections was extracted by using RNeasy Lipid Tissue Mini kit (Qiagen, Hilden, Germany). RNA quality was evaluated with an Agilent Bioanalyzer (Agilent Technologies, Santa Clara, CA, USA) and quantified by spectrophotometry. Biotinylated RNA was amplified by using the TotalPrep RNA amplification kit (Ambion) and hybridized with Illumina Sentrix Expression Beadchips, Human HT-12v4 (Illumina, San Diego, CA, USA). Each array contains more than 31,000 probed genes. Microarray data have been submitted to the public repository, Gene Expression Omnibus.

### Reverse transcription and qRT-PCR

Skin RNA from 17 patients with DLE and 12 normal controls was reverse-transcribed into cDNA by using the iScript cDNA Synthesis kit (Bio-Rad Laboratories, Hercules, CA, USA). cDNA of selected genes using forward and reverse primers (Additional file 1: Table S1) was amplified with SYBR Green PCR Master Mix (Applied Biosystems, Foster City, CA, USA) in accordance with the instructions of the manufacturer. qRT-PCR under the following conditions (3 minutes at 95 °C, then 40 cycles of 20 seconds at 95 °C, 1 minute at 55 °C, and 30 seconds at 72 °C) was performed in a CFX96 qRT-PCR machine (Bio-Rad Laboratories). Cycle threshold (C_T_) values were standardized to the housekeeping gene GAPDH (glyceraldehyde-3-phosphate dehydrogenase) and converted to fold change by using the 2^−ΔΔCT^ formula [19].

### Immunohistochemistry analysis

A subset of DLE lesional (n = 5) and normal skin (n = 6) biopsies were bisected and transferred in 10 % formalin. Four-micron sections were deparaffinized in xylene and rehydrated in graded alcohols to distilled water. Endogenous peroxidase activity was quenched for 10 minutes at room temperature by using 0.3 % H_2_O_2_ and 0.1 % sodium azide. For epitope retrieval, slides were placed in 1 mM EDTA, pH 8.5, for 30 minutes in a steamer and then cooled for 10 minutes. Slides were incubated with mouse monoclonal anti-CD163 antibody (Neomarkers/Thermo Fisher Scientific, Waltham, MA, USA) or isotype control for 50 minutes at 25 °C [20]. After phosphate-buffered saline (PBS) rinse, horseradish peroxidase-conjugated goat anti-rabbit horseradish peroxidase-conjugated IgG antibody (Leica Novocastra, Wetzlar, Germany) [21] was added for 45 minutes at 25 °C [20]. Finally, the slides were immersed for 8 minutes in 25 °C diaminobenzidine (Invitrogen, Carlsbad, CA, USA), enhanced with 0.5 % copper sulfate in PBS for 1–3 minutes at 25 °C, counterstained in hematoxylin, and dehydrated in graded alcohols and xylene. Cell counts were performed by two independent evaluators (BC and GH) on the most representative sections in the epidermal-dermal junction, perifollicular, and perivascular areas of the biopsies by using ImageJ [22]. Cell counts were averaged and divided by surface area assessed.

### Immunofluorescence

Six-micron frozen DLE (n = 6–8) and normal (n = 4–5) skin sections fixed in acetone were blocked in 5 % normal goat serum in PBS with 0.3 % Triton X-100 for 1 hour. Slides were then incubated with rabbit anti-human primary antibodies—CD127 (Abcam, Cambridge, MA, USA), CD209 (Abcam), CXCL10 (PeproTech, Rocky Hill, NJ, USA), tumor necrosis factor-α (TNF-α) (Novus, Littleton, CO, USA), and TGF-β (Novus)—overnight followed by PBS washes. Alexa Fluor^®^ 488-conjugated goat anti-rabbit antibodies (Life Technologies, Carlsbad, CA, USA) were added for 30 minutes followed by PBS washes, except for TGF-β and TNF-α antibody-stained slides, whose signals were amplified by using the TSA Biotin kit in accordance with the instructions of the manufacturer (PerkinElmer, Waltham, MA, USA). Mouse anti-human CD163 antibody (Novus) or mouse anti-human CD3 antibody (eBioscience, Inc., San Diego, CA, USA) was added for 1 hour, followed by PBS washes. Slides were incubated with Alexa Fluor^®^ 594-conjugated goat anti-mouse antibodies (Life Technologies) for 30 minutes and washed with PBS. Slides were coverslipped with Vectashield mounting medium and analyzed. Images were acquired with an Olympus BX60 fluorescent microscope (Olympus, Tokyo, Japan).

### Statistical analysis

Based on 31,000 probed genes on the Illumina Human HT-12v4 Expression BeadChip, a sample size of eight for each study group was used and this had predicted power to detect twofold gene expression difference with more than 80 % power at a 5 % false-positive rate assuming a standard deviation of 0.65 [23]. Microarray data were quantum-normalized and background-subtracted by using Bead Studio Software version 3.2.2 and analyzed on GeneSpring GX (Agilent Technologies, Santa Clara, CA, USA). To connect genes of interest with existing biological pathways, Ingenuity Pathway Analysis (Ingenuity System Inc., Redwood City, CA, USA) was used to identify networks ordered by *P* values. Significance Analysis of Microarrays (SAM) [24] helped identify genes with statistically significant differences; q values, defined as the lowest false discovery rate at which genes were called significant by SAM analysis [25], were less than 0.05. Gene set enrichment analysis was performed to determine whether differentially expressed genes were over-represented in referenced gene lists [26], specifically those from macrophages incubated with either interferon-gamma (IFN-γ) (M1 macrophages) or IL-4 (M2 macrophages) [17]. Heat maps and clustering analyses were performed by using Cluster and Treeview software [27]. For qRT-PCR and immunohistochemistry data, Mann-Whitney *U* test was used to compare the two study groups.

## Results

### Transcriptomes of DLE lesional skin were enriched with M1 macrophage-related genes

**Fig. 1:**
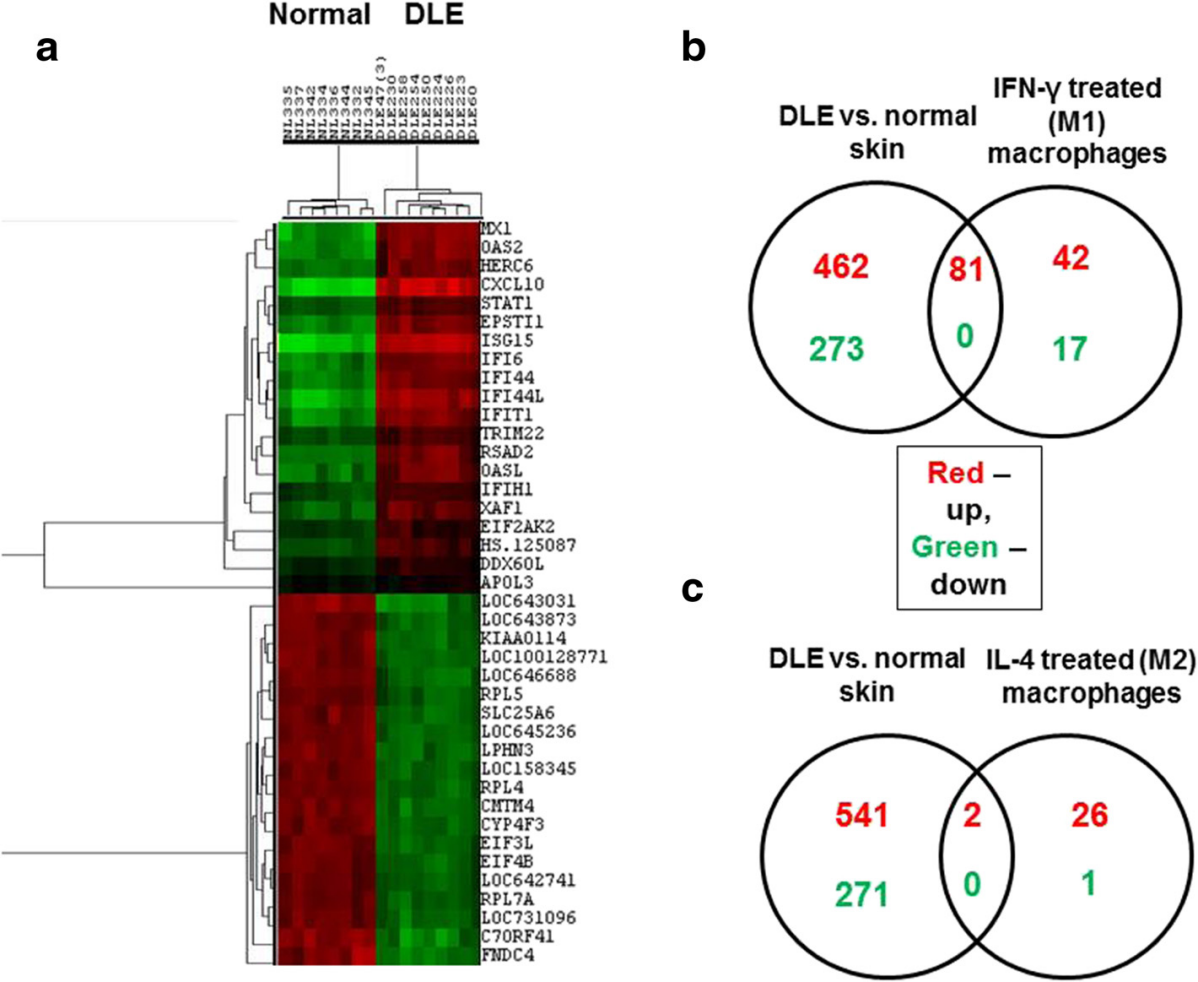
Microarray analysis of DLE lesional and normal skin. **a** Heat map depicts the top 20 upregulated and downregulated genes in DLE lesional skin (n = 9) versus normal skin (n = 8) (more than twofold, q < 0.05). *Red* and *green* represent high and low expression, respectively. **b**, **c** Venn diagrams of differentially expressed genes in DLE versus normal skin, and macrophages treated with IFN-γ (M1) (**b**) or IL-4 (M2) (**c**) [17] demonstrated that more upregulated genes in DLE skin were shared with those in IFN-γ-treated macrophages than IL-4 treated macrophages. *DLE* discoid lupus erythematosus, *IFN-γ* interferon-gamma, *IL-4* interleukin-4

From a total of 21,628 genes, whose expression was either “present” or “marginal” in at least 75 % of all samples, microarray analysis of DLE lesional skin (n = 9) versus normal sun-exposed skin (n = 8) revealed 543 upregulated and 273 downregulated genes in DLE skin (more than twofold, q < 0.05) (Fig. 1a and Additional file 1: Table S2). Pathway analysis showed that the two most significant signaling cascades included genes involved in antigen presentation and cytotoxic T cell-mediated apoptosis. Because IFN-γ was the top upstream regulator gene, these findings supported a prominent role of cell-mediated immunity in DLE pathogenesis. Specifically, we noted several upregulated genes in DLE skin that may be expressed by different cells associated with cell-mediated immunity (e.g., T_H_1 cells: CXCR3, signal transducer and activator of transcription 1 (STAT1), and STAT4; CD8^+^ cells: granzymes A, B, K, and perforin; and macrophages: CD68, CD163, and CXCL10) (highlighted in Additional file 1: Table S2).

We then focused on analyzing expression of macrophage-related genes in DLE versus normal skin to investigate macrophage polarization (M1 or M2) in DLE skin. We first generated Venn diagrams to identify common genes in our lists of differentially expressed genes in DLE and normal skin and previously published sets of differentially expressed genes in macrophages before and after treatment with IFN-γ (M1 macrophages) [17]. Eighty-one genes were commonly upregulated in both gene sets, but none was mutually downregulated (Fig. 1b and Additional file 1: Table S3). Differentially expressed genes in DLE and normal skin and macrophages before and after treatment with IL-4 (M2 macrophages) [17] were also compared. Only two genes were enhanced in both gene sets, and none was commonly decreased (Fig. 1c and Additional file 1: Table S3). Gene set enrichment analysis showed that upregulated genes in DLE skin showed significant overlap with those in M1 macrophages (normalized enrichment score = 1.25, *P* = 0.02) but not with those in M2 macrophages (Table 2). Thus, both analyses showed that DLE skin was significantly enriched with M1 macrophage-related genes.

**Table 2:** Gene set enrichment analysis of differentially expressed genes in DLE lesional and normal skin

Name	Size	ES	NES	*P* value	FDR q value
IFN-γ-stimulated (M1) macrophages upregulated	113	0.92	1.25	0.02	0.02
IFN-γ stimulated (M1) macrophages downregulated	16	−0.55	−1.24	0.25	0.25
IL-4 stimulated (M2) macrophages upregulated	26	0.39	0.97	0.49	0.49
IL-4 stimulated (M2) macrophages downregulated	1	0.95	1.11	0.27	0.27

Abbreviations: *DLE* discoid lupus erythematosus, *ES* enrichment score, *FDR* false discovery rate, *IFN-γ* interferon-gamma, *IL-4* interleukin-4, *NES* normalized enrichment score

### qRT-PCR analysis confirms upregulation of multiple M1 macrophage-related genes in DLE skin

**Fig. 2:**
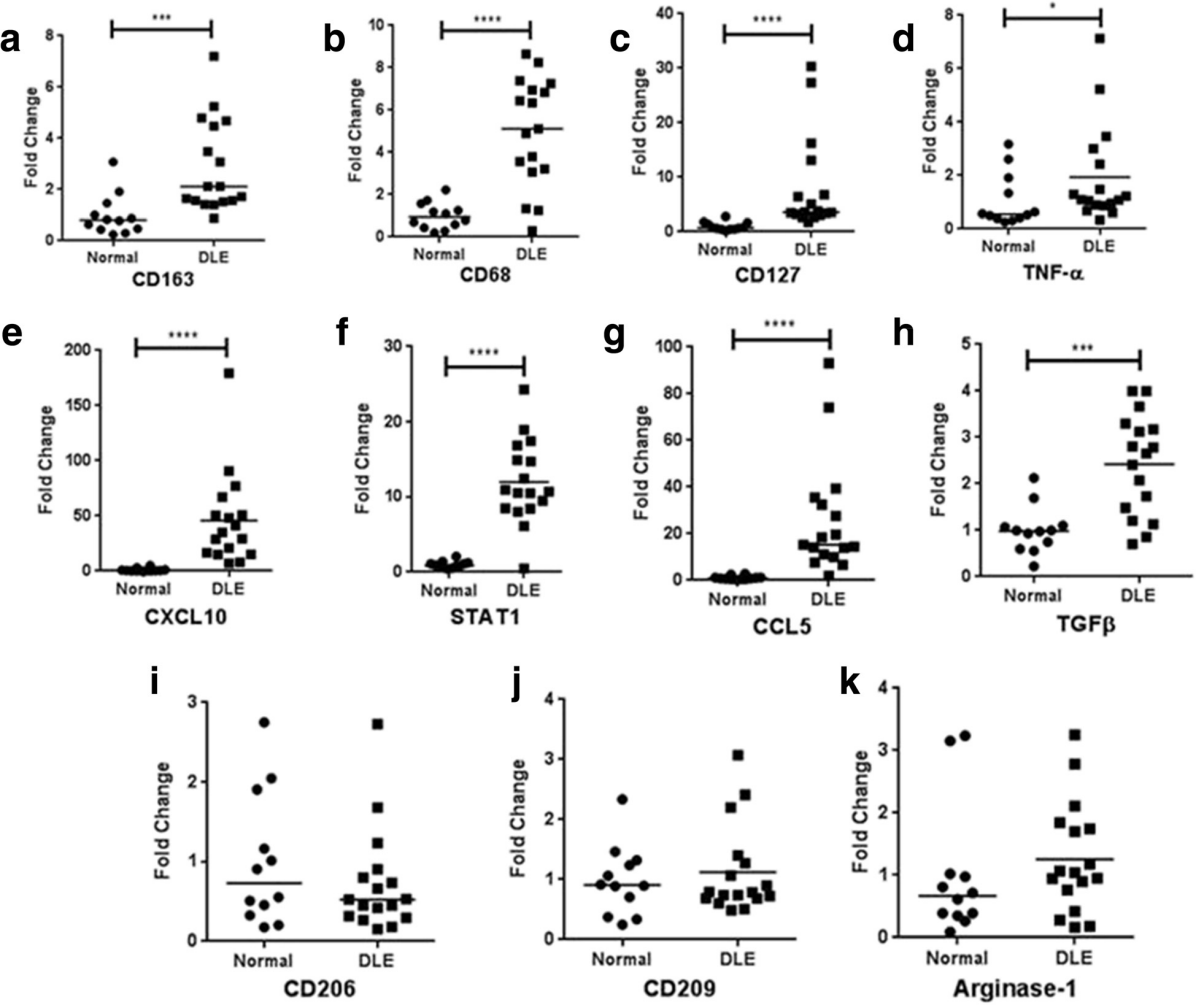
qRT-PCR analysis of selected genes in DLE lesional and normal skin. **a**–**k** RNA expression of general macrophage markers (CD163 and CD68) (**a**, **b**), M1 macrophage-related markers (CD127, TNF-α, CXCL10, STAT1, and CCL5) (**c**–**g**), and M2 macrophage-related markers (TGF-β, CD206, CD209, and arginase-1) (**h**–**k**) was compared in DLE lesional (n = 17) and normal (n = 12) skin via qRT-PCR. Multiple general and M1 macrophage-related markers were upregulated in DLE lesional skin. *P ≤ 0.05, **P ≤ 0.01, ***P ≤ 0.001, ****P ≤ 0.0001. *CCL* chemokine (C-C motif) ligand, *CXCL* chemokine (C-X-C motif) ligand, *DLE* discoid lupus erythematosus, *qRT-PCR* quantitative real-time polymerase chain reaction, *STAT* signal transducer and activator of transcription, *TGF-β* transforming growth factor-beta, *TNF-α* tumor necrosis factor-alpha

Several macrophage-related genes were selected for confirmatory qRT-PCR studies on DLE lesional (n = 17) and normal sun-exposed (n = 12) skin. CD163 (2.83 fold change (FC)) and CD68 (4.98 FC), which are both markers used to identify macrophages, had significantly higher levels in DLE skin than normal skin (*P* < 0.0001) (Fig. 2a, b). Multiple M1 macrophage genes—e.g., CD127 (8.01 FC, *P* < 0.0001), TNF-α (1.92 FC, *P* = 0.047), CXCL10 (45.91 FC, *P* < 0.0001), STAT1 (11.96 FC, *P* < 0.0001), CCL5 (25.85 FC, *P* < 0.0001), CD86, and Mx1—were also significantly upregulated in DLE skin compared with normal skin (Fig. 2c–g and Additional file 1: Table S4). IFN-γ and IL-12, two cytokines associated with M1 macrophages, were higher in DLE skin but did not reach statistical significance (Additional file 1: Table S4). With the exception of TGF-β (2.42 FC, *P* = 0.0002), which was significantly higher in DLE skin than normal skin, multiple M2 macrophage genes—e.g., CD206 (0.72 FC, *P* = 0.32), CD209 (1.10 FC, *P* = 0.97), arginase-1 (1.25 FC, *P* = 0.19), FOLR2, and IL-10—were not differentially expressed in DLE and normal skin (Fig. 2h–k and Additional file 1: Table S4). We also selected type I interferon-inducible genes (e.g., Mx1, ISG15, and Ly6E) and genes associated with T_H_1 cells (e.g., CXCR3) and CD8^+^ T cells (e.g., CD8 and granzyme B) for qRT-PCR analysis. These were significantly elevated in DLE skin versus normal skin (*P* < 0.0001) (Additional file 1: Table S4).

### DLE lesional skin contained higher numbers of CD163^+^ macrophages than normal skin

**Fig. 3:**
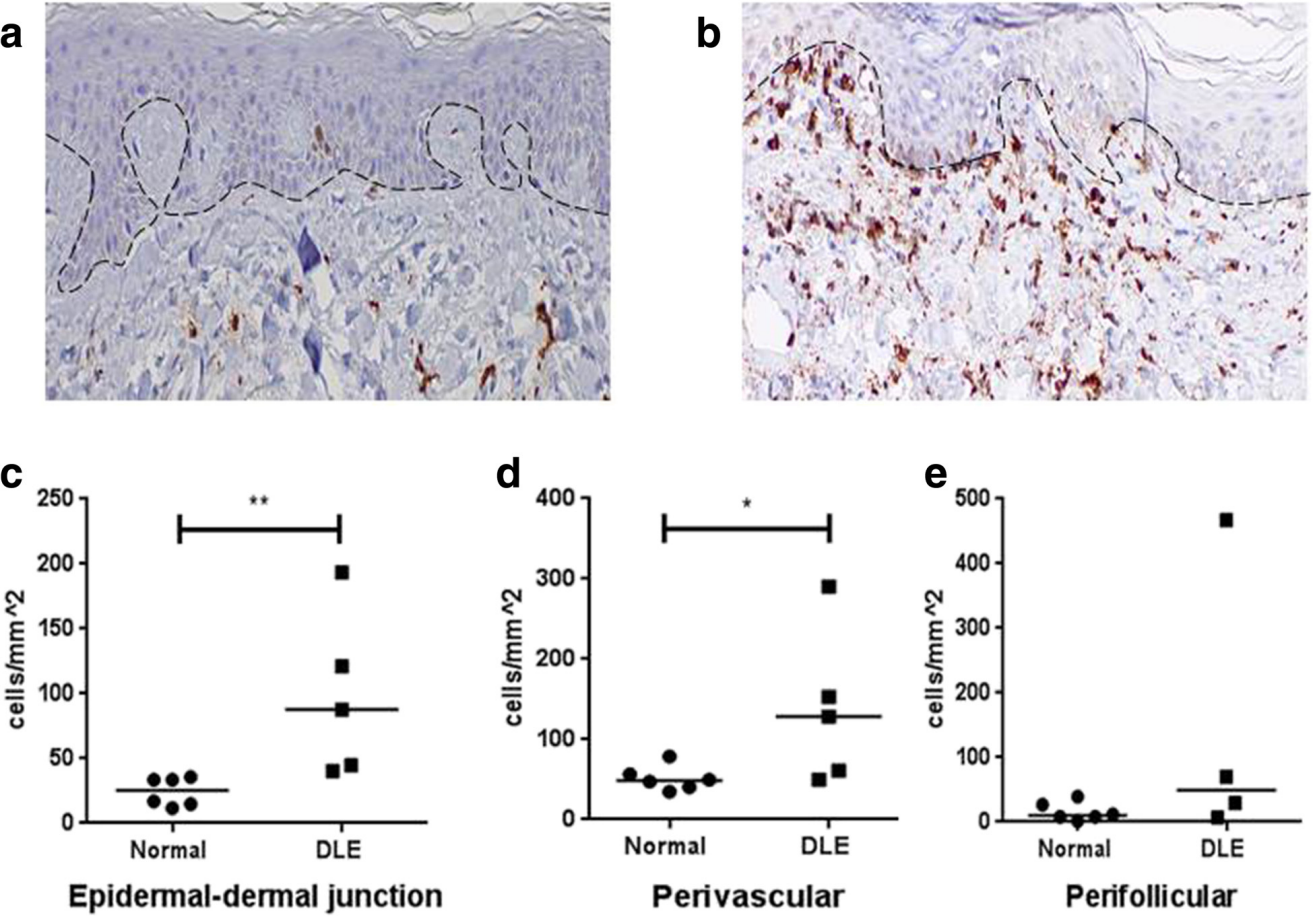
CD163^+^ macrophages in normal skin and DLE lesional skin. **a**, **b** Immunohistochemical analysis of CD163 was performed in normal skin (n = 6) (**a**) and DLE lesional skin (n = 5) (**b**). Total magnification: ×200. **c**–**e** CD163^+^ cell counts from representative high-powered fields of the epidermal-dermal junction (**c**), perivascular areas (**d**), and perifollicular areas (**e**) of normal and DLE lesional skin showed significantly higher numbers of CD163^+^ macrophages in the epidermal-dermal junction and perivascular areas of DLE skin. *P ≤ 0.05, **P ≤ 0.01. *DLE* discoid lupus erythematosus

Samples of DLE lesional skin (n = 5) and normal sun-exposed skin (n = 6) were immunostained with CD163, a scavenger receptor that is highly specific for macrophages in the skin [28] (Fig. 3a, b). DLE skin had significantly higher concentrations of CD163^+^ cells at the epidermal-dermal junction (median: 87.8 cells/mm^2^ versus 25.3 cells/mm^2^; *P* = 0.004) and perivascular areas (median: 128.9 cells/mm^2^ versus 48.6 cells/mm^2^; *P* = 0.03) than normal skin. DLE skin showed higher amounts of CD163^+^ cells in perifollicular areas compared with normal skin (median: 50.0 cells/mm^2^ versus 10.0 cells/mm^2^), but this difference did not reach statistical significance (*P* = 0.26) (Fig. 3c–e).

### A minority of CD163^+^ macrophages showed co-staining with M1- and M2-related proteins in DLE skin

**Fig. 4:**
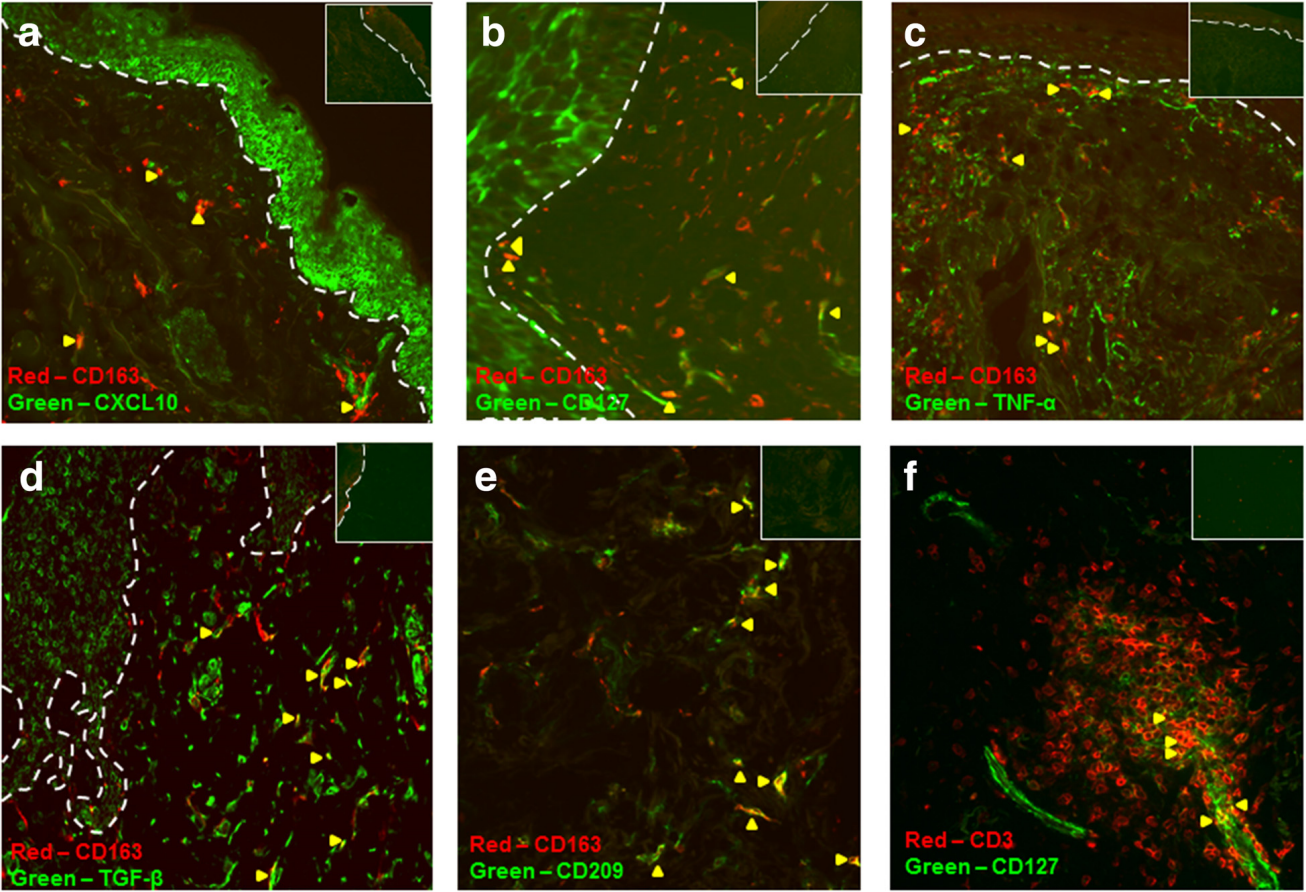
Double immunofluorescence staining for CD163^+^ macrophages and CD3^+^ T cells and selected macrophage markers in DLE lesional skin (n = 6–8). **a**–**e** DLE lesional skin was stained with both antibodies to CD163 (*red*) and selected macrophage markers (*green*), including CXCL10 (**a**), CD127 (**b**), TNF-α (**c**), TGF-β (**d**), and CD209 (**e**). Accompanying control slides are present on the *upper right. Yellow arrowheads* indicate example areas of overlap. A minority of CD163^+^ macrophages co-stained with these proteins, and the most overlap was seen with CD209. (**f**) DLE lesional skin was also stained with antibodies to CD3 (*red*) and CD127 (*green*). A subset of CD3^+^ T cells showed expression of CD127. *Dotted line* denotes epidermal-dermal junction. Total magnification: ×200. *DLE* discoid lupus erythematosus, *TGF-β* transforming growth factor-beta, *TNF-α* tumor necrosis factor-alpha

To determine whether CD163^+^ macrophages predominantly expressed M1 or M2 macrophage-related proteins or both, double immunostaining of CD163 and selected M1 and M2 macrophage-related proteins (CXCL10, CD127, TNF-α, TGF-β, and CD209) were performed in DLE lesional (n = 6–8) and normal (n = 4 or 5) skin. DLE and normal skin both showed a minority of CD163^+^ macrophages near the epidermal-dermal junction and in the perivascular and periadnexal areas of the dermis co-expressing CXCL10, CD127, TNF-α, TGF-β, and CD209, and the greatest overlap was seen with CD209 (Fig. 4a–e). Because of this result, we postulated that other inflammatory cells such as T cells could also contribute to the upregulation of M1 macrophage-related proteins in DLE lesional skin. Co-staining of a minority of CD3^+^ T cells with CD127 was detected in the dermis of DLE skin (Fig. 4f).

## Discussion

Transcriptome analysis of DLE lesional skin yielded strong expression of a signature of M1 macrophage-related genes. Inspection of selected M1 and M2 macrophage-related genes by qRT-PCR confirmed upregulation of multiple M1 macrophage-related genes in DLE skin. Because M1 macrophages are activated by IFN-γ that is produced by T_H_1 cells and promote T_H_1 responses through their antimicrobial activity [29], our findings are consistent with earlier studies showing a predominant T_H_1 bias in DLE skin [30]. Moreover, we performed gene set enrichment analysis comparing previously published gene sets of T_H_1 and T_H_2 cells [31] with DLE and normal skin transcriptome data and detected T_H_1-biased genes being highly expressed in DLE skin (*P* = 0.01) (data not shown).

Given the limitations that whole skin biopsies rather than isolated CD163^+^ skin macrophages were studied for transcriptome analysis and that several upregulated genes may be expressed by skin cells other than macrophages, we performed double immunofluorescence staining to examine protein expression of CD163^+^ macrophages in DLE skin. Some CD163^+^ macrophages showed co-expression of various M1 (e.g., CD127, CXCL10, and TNF-α) and M2 (CD209 and TGF-β) macrophage-related proteins, and the greatest overlap was seen with CD209. Although CD163 has been reported by in vitro studies to be a M2-related macrophage marker [32, 33], immunostaining studies of CD163^+^ macrophages in peripheral tissues of different diseases have shown that they can express transcription factors specific to either M1 or M2 macrophage signaling pathways [34]. Specifically, studies on squamous cell carcinoma of the skin have reported that CD163^+^ macrophages can produce both M1 and M2 macrophage-related proteins [35]. The presence of different macrophage subtypes may result from the ability of macrophages to change from one subtype to another in response to various stimuli from the surrounding milieu. For instance, IL-4 can guide the transformation of M1 macrophages into M2 macrophages, which promote wound healing and angiogenesis [36]. Likewise, cytokines such as IFN-γ produced by T_H_1 cells can alter the polarization of M2 macrophages to M1 macrophages that subsequently produce M1 macrophage-related cytokines and enhance their co-stimulatory molecule expression [37]. Furthermore, exogenous signals such as bacterial lipopolysaccharides can induce both M1 and M2 macrophages to produce M1 macrophage-related cytokines such as TNF-α and IL-1 [38].

The disease phase at the time of DLE skin biopsy may have impacted the immunostaining characterization of CD163^+^ macrophages. In the acute phase, DLE lesions present clinically as erythematous scaly papules and plaques and microscopically have interface dermatitis, scattered apoptotic keratinocytes, mucin deposition, and perivascular and periadnexal inflammatory infiltrates [39]. DLE lesions later evolve into hypopigmented scarred plaques with peripheral hyperpigmentation in the chronic phase, and their histopathology displays epidermal atrophy, mild interface dermatitis, loss of hair follicles, and dermal sclerosis [39]. M2 macrophages upregulate IL-10, which has anti-inflammatory properties. TGF-β is a pro-fibrotic cytokine produced mainly by M2c macrophages [40] and may contribute to the scarring process that is seen in the latter stages of DLE. Furthermore, TGF-β can have anti-inflammatory effects by downregulating production of pro-inflammatory cytokines such as TNF-α [41] and maintaining peripheral tolerance by limiting the expansion of self-reactive T cells [42]. Because many of these biopsied discoid lesions had clinical features of both acute and chronic DLE, we hypothesize that these lesions may be in a transitional phase between acute and chronic DLE, and this could explain the heterogeneity of macrophage subtypes. Further projects that compare protein expression in acute and chronic discoid skin lesions will be needed to test whether macrophages strongly favor one polarization over another in the two phases of DLE.

Macrophage bias in peripheral organs other than skin has been investigated in murine models with lupus nephritis. In lupus-prone MRL-lpr mice, transient ischemia/reperfusion injury resulted in early-onset lupus nephritis with an accompanying increase in M1 macrophages compared with M2 macrophages [43]. However, in other lupus murine models, heterogeneous populations of macrophages that did not show a distinct M1 or M2 bias were described in chronic lupus nephritis kidneys [44]. Because human lupus nephritis and DLE have similar features of acute inflammation and chronic scarring, comparisons of macrophages in these two end-organ diseases in lupus could be made to better understand disease pathogenesis in lupus.

Our double immunofluorescence data also showed that other cells, particularly T cells, contribute to enhanced levels of M1 macrophage-related proteins. A minority of CD3^+^ T cells showed co-expression of CD127, an M1 macrophage-related protein, in DLE lesional skin. T cells have been described to express other M1 macrophage-related proteins, including IFN-γ [30]. Moreover, given that a T_H_1 cell bias has been observed in DLE skin in our and other [30] data and that T_H_1 cells can activate classically activated macrophages through their secretion of IFN-γ, T_H_1 cells likely contribute to the M1 macrophage gene signature in DLE skin. Additionally, CD8^+^ T cells, which also produce IFN-γ and activate macrophages, have been observed in greater numbers in DLE skin [12, 45, 46]. Thus, the cross-talking between T_H_1 cells, CD8^+^ T cells, and macrophages implies that cell-mediated immunity can play a significant role in the pathogenesis of DLE. Further studies examining their interactions and impact on evolution of DLE will be planned. As DLE skin is enriched with multiple types of immune cells [13, 46], our qRT-PCR data demonstrated that DLE skin upregulates cell surface markers expressed by dendritic cells (CD86) and monocytes (CD14), which can also express M1 macrophage-related proteins (e.g., CXCL10 [47, 48] and ISG15 [49]).

## Conclusions

Whereas transcriptome analysis suggested an M1 macrophage gene signature in DLE skin, immunohistochemical studies of DLE skin uncovered a subset of CD163^+^ macrophages expressing both M1 and M2 macrophage-related proteins. The diversity of macrophage subtypes may be due to their gene expression plasticity and a mixture of acute and chronic phases in the DLE biopsy skin specimens. Moreover, other immune cells, such as CD3^+^ T cells, infiltrating into DLE skin can contribute to the M1 macrophage gene signature in DLE skin. Further exploration into the multiple immune cells expressing M1 macrophage-related proteins and their interactions with each other will help further explain the evolution of DLE.

## Additional file

Additional file 1:
**Table S1.** Forward and reverse primers for selected genes. **Table S2.** Differentially expressed genes in DLE lesional (n = 9) and normal (n = 8) skin from microarray analysis (more than twofold change, q value < 0.05). **Table S3.** List of commonly differentially expressed genes in DLE and normal skin, and IFN-γ treated (M1) and IL-4 treated (M2) macrophages. **Table S4.** qRT-PCR analysis of selected genes in DLE lesional (n = 17) and normal (n = 12) skin. *DLE* discoid lupus erythematosus, *IFN-γ* interferon-gamma, *qRT-PCR* quantitative real-time polymerase chain reaction. (DOCX 165 kb)

## Abbreviations

ACR: American College of Rheumatology
CCL: Chemokine (C-C motif) ligand
CLASI: Cutaneous Lupus Activity and Severity Index
CSF: Colony-stimulating factor
CXCL: Chemokine (C-X-C motif) ligand
DLE: Discoid lupus erythematosus
IFN: Interferon
IL: Interleukin
qRT-PCR: Quantitative real-time polymerase chain reaction
SAM: Significance Analysis of Microarrays
SLE: Systemic lupus erythematosus
SLEDAI: Systemic Lupus Erythematosus Disease Activity Index
STAT: Signal transducer and activator of transcription
TGF: Transforming growth factor
TNF: Tumor necrosis factor
UTSW: University of Texas Southwestern

## References

[1] Durosaro O, Davis MD, Reed KB, Rohlinger AL (2009). Incidence of cutaneous lupus erythematosus, 1965–2005: a population-based study. Arch Dermatol..

[2] Cardinali C, Caproni M, Bernacchi E, Amato L, Fabbri P (2000). The spectrum of cutaneous manifestations in lupus erythematosus--the Italian experience. Lupus..

[3] Tebbe B, Orfanos CE (1997). Epidemiology and socioeconomic impact of skin disease in lupus erythematosus. Lupus..

[4] Callen JP (1982). Chronic cutaneous lupus-erythematosus - clinical, laboratory, therapeutic, and prognostic examination of 62 patients. Arch Dermatol..

[5] Gronhagen CM, Gunnarsson I, Svenungsson E, Nyberg F (2010). Cutaneous manifestations and serological findings in 260 patients with systemic lupus erythematosus. Lupus..

[6] Pons-Estel BA, Catoggio LJ, Cardiel MH, Soriano ER, Gentiletti S, Villa AR (2004). The GLADEL multinational Latin American prospective inception cohort of 1,214 patients with systemic lupus erythematosus: ethnic and disease heterogeneity among “Hispanics”. Medicine (Baltimore).

[7] Jacobsen S, Petersen J, Ullman S, Junker P, Voss A, Rasmussen JM (1998). A multicentre study of 513 Danish patients with systemic lupus erythematosus. I. Disease manifestations and analyses of clinical subsets. Clin Rheumatol.

[8] Healy E, Kieran E, Rogers S (1995). Cutaneous lupus erythematosus--a study of clinical and laboratory prognostic factors in 65 patients. Ir J Med Sci..

[9] Gronhagen CM, Fored CM, Granath F, Nyberg F (2011). Cutaneous lupus erythematosus and the association with systemic lupus erythematosus: a population-based cohort of 1088 patients in Sweden. Br J Dermatol..

[10] Dey-Rao R, Sinha AA (2015). Genome-wide transcriptional profiling of chronic cutaneous lupus erythematosus (CCLE) peripheral blood identifies systemic alterations relevant to the skin manifestation. Genomics..

[11] Dey-Rao R, Smith JR, Chow S, Sinha AA (2014). Differential gene expression analysis in CCLE lesions provides new insights regarding the genetics basis of skin vs. systemic disease. Genomics.

[12] Wenzel J, Zahn S, Mikus S, Wiechert A, Bieber T, Tuting T (2007). The expression pattern of interferon-inducible proteins reflects the characteristic histological distribution of infiltrating immune cells in different cutaneous lupus erythematosus subsets. Br J Dermatol..

[13] Thorpe RB, Gray A, Kumar KR, Susa JS, Chong BF (2014). Site-specific analysis of inflammatory markers in discoid lupus erythematosus skin. ScientificWorldJournal..

[14] Hussein MR, Aboulhagag NM, Atta HS, Atta SM (2008). Evaluation of the profile of the immune cell infiltrate in lichen planus, discoid lupus erythematosus, and chronic dermatitis. Pathology..

[15] Bauer JW, Petri M, Batliwalla FM, Koeuth T, Wilson J, Slattery C (2009). Interferon-regulated chemokines as biomarkers of systemic lupus erythematosus disease activity: a validation study. Arthritis Rheum..

[16] Becker-Merok A, Eilertsen GO, Nossent JC (2010). Levels of transforming growth factor-beta are low in systemic lupus erythematosus patients with active disease. J Rheumatol..

[17] Fuentes-Duculan J, Suarez-Farinas M, Zaba LC, Nograles KE, Pierson KC, Mitsui H (2010). A subpopulation of CD163-positive macrophages is classically activated in psoriasis. J Invest Dermatol..

[18] Tan EM, Cohen AS, Fries JF, Masi AT, Mcshane DJ, Rothfield NF (1982). Special Article - the 1982 Revised Criteria for the Classification of Systemic Lupus-Erythematosus. Arthritis Rheum..

[19] Livak KJ, Schmittgen TD (2001). Analysis of relative gene expression data using real-time quantitative PCR and the 2(-Delta Delta C(T)) Method. Methods..

[20] Butz WR, Clark KC, Miller RT (1994). Improved manual immunohistochemistry employing orbital mixing of reagents during incubations. Appl Immunohistochem..

[21] Shi SR, Guo J, Cote RJ, Young LL, Hawes D, Shi Y (1999). Sensitivity and detection efficiency of a novel two-step detection system (PowerVision) for immunohistochemistry. Appl Immunohisto M M..

[22] Schneider CA, Rasband WS, Eliceiri KW (2012). NIH Image to ImageJ: 25 years of image analysis. Nat Methods..

[23] Sample Size for Microarray Experiments. http://bioinformatics.mdanderson.org/MicroarraySampleSize. Accessed 4 Nov. 2015.

[24] SAM: Significance Analysis of Microarrays. http://statweb.stanford.edu/~tibs/SAM. Accessed 4 Nov. 2015.

[25] Tusher VG, Tibshirani R, Chu G (2001). Significance analysis of microarrays applied to the ionizing radiation response. Proc Natl Acad Sci U S A..

[26] Subramanian A, Tamayo P, Mootha VK, Mukherjee S, Ebert BL, Gillette MA (2005). Gene set enrichment analysis: a knowledge-based approach for interpreting genome-wide expression profiles. Proc Natl Acad Sci U S A..

[27] http://bonsai.hgc.jp/~mdehoon/software/cluster/software.htm.. Accessed 4 Nov. 2015.

[28] Zaba LC, Fuentes-Duculan J, Steinman RM, Krueger JG, Lowes MA (2007). Normal human dermis contains distinct populations of CD11c + BDCA-1+ dendritic cells and CD163 + FXIIIA+ macrophages. J Clin Invest..

[29] Wang N, Liang H, Zen K (2014). Molecular mechanisms that influence the macrophage m1-m2 polarization balance. Front Immunol..

[30] Jabbari A, Suarez-Farinas M, Fuentes-Duculan J, Gonzalez J, Cueto I, Franks AG (2014). Dominant Th1 and minimal Th17 skewing in discoid lupus revealed by transcriptomic comparison with psoriasis. J Invest Dermatol..

[31] Zhang H, Nestor CE, Zhao S, Lentini A, Bohle B, Benson M (2013). Profiling of human CD4+ T-cell subsets identifies the TH2-specific noncoding RNA GATA3-AS1. J Allergy Clin Immunol..

[32] Buechler C, Ritter M, Orso E, Langmann T, Klucken J, Schmitz G (2000). Regulation of scavenger receptor CD163 expression in human monocytes and macrophages by pro- and antiinflammatory stimuli. J Leukoc Biol..

[33] Sulahian TH, Hogger P, Wahner AE, Wardwell K, Goulding NJ, Sorg C (2000). Human monocytes express CD163, which is upregulated by IL-10 and identical to p155. Cytokine..

[34] Barros MH, Hauck F, Dreyer JH, Kempkes B, Niedobitek G (2013). Macrophage polarisation: an immunohistochemical approach for identifying M1 and M2 macrophages. PLoS One..

[35] Pettersen JS, Fuentes-Duculan J, Suarez-Farinas M, Pierson KC, Pitts-Kiefer A, Fan L (2011). Tumor-associated macrophages in the cutaneous SCC microenvironment are heterogeneously activated. J Invest Dermatol..

[36] Spiller KL, Nassiri S, Witherel CE, Anfang RR, Ng J, Nakazawa KR (2015). Sequential delivery of immunomodulatory cytokines to facilitate the M1-to-M2 transition of macrophages and enhance vascularization of bone scaffolds. Biomaterials..

[37] Heusinkveld M, de Vos van Steenwijk PJ, Goedemans R, Ramwadhdoebe TH, Gorter A, Welters MJ (2011). M2 macrophages induced by prostaglandin E2 and IL-6 from cervical carcinoma are switched to activated M1 macrophages by CD4+ Th1 cells. J Immunol..

[38] Gratchev A, Kzhyshkowska J, Kothe K, Muller-Molinet I, Kannookadan S, Utikal J (2006). Mphi1 and Mphi2 can be re-polarized by Th2 or Th1 cytokines, respectively, and respond to exogenous danger signals. Immunobiology..

[39] Baltaci M, Fritsch P (2009). Histologic features of cutaneous lupus erythematosus. Autoimmun Rev..

[40] Lu J, Cao Q, Zheng D, Sun Y, Wang C, Yu X (2013). Discrete functions of M2a and M2c macrophage subsets determine their relative efficacy in treating chronic kidney disease. Kidney Int..

[41] Bogdan C, Paik J, Vodovotz Y, Nathan C (1992). Contrasting mechanisms for suppression of macrophage cytokine release by transforming growth factor-beta and interleukin-10. J Biol Chem..

[42] Sanjabi S, Zenewicz LA, Kamanaka M, Flavell RA (2009). Anti-inflammatory and pro-inflammatory roles of TGF-beta, IL-10, and IL-22 in immunity and autoimmunity. Curr Opin Pharmacol..

[43] Iwata Y, Bostrom EA, Menke J, Rabacal WA, Morel L, Wada T (2012). Aberrant macrophages mediate defective kidney repair that triggers nephritis in lupus-susceptible mice. J Immunol..

[44] Sahu R, Bethunaickan R, Singh S, Davidson A (2014). Structure and function of renal macrophages and dendritic cells from lupus-prone mice. Arthritis Rheumatol..

[45] Wenzel J, Uerlich M, Worrenkamper E, Freutel S, Bieber T, Tuting T (2005). Scarring skin lesions of discoid lupus erythematosus are characterized by high numbers of skin-homing cytotoxic lymphocytes associated with strong expression of the type I interferon-induced protein MxA. Br J Dermatol..

[46] Xie Y, Jinnin M, Zhang X, Wakasugi S, Makino T, Inoue Y (2011). Immunohistochemical characterization of the cellular infiltrate in discoid lupus erythematosus. Biosci Trends..

[47] Hong CY, Lee HJ, Kim HJ, Lee JJ (2014). The lymphoid chemokine CCL21 enhances the cytotoxic T lymphocyte-inducing functions of dendritic cells. Scand J Immunol..

[48] Luster AD, Ravetch JV (1987). Biochemical characterization of a gamma interferon-inducible cytokine (IP-10). J Exp Med..

[49] D’Cunha J, Ramanujam S, Wagner RJ, Witt PL, Knight E, Borden EC (1996). In vitro and in vivo secretion of human ISG15, an IFN-induced immunomodulatory cytokine. J Immunol..

