# Supplementary material for: A subset of CD163+ macrophages displays mixed polarizations in discoid lupus skin
Source: Arthritis Res Ther. 2015 Nov 13;17:324. doi: 10.1186/s13075-015-0839-3 (PMC4644297; doi:10.1186/s13075-015-0839-3)
Supplement: Additional file 1: — Table S1. Forward and reverse primers for selected genes. Table S2. Differentially expressed genes in DLE lesional (n = 9) and normal (n = 8) skin from microarray analysis (more than twofold change, q value < 0.05). Table S3. List of commonly differentially expressed genes in DLE and normal skin, and IFN-γ treated (M1) and IL-4 treated (M2) macrophages. Table S4. qRT-PCR analysis of selected genes in DLE lesional (n = 17) and normal (n = 12) skin. DLE discoid lupus erythematosus, IFN-γ interferon-gamma, qRT-PCR quantitative real-time polymerase chain reaction. (DOCX 165 kb) [file 13075_2015_839_MOESM1_ESM.docx]

Table S1. Forward and reverse primers for selected genes.

| Gene Symbol | Forward primer | Reverse primer |
| --- | --- | --- |
| ARG1 | 5'-TCATCTGGGTGGATGCTCACAC-3' | 5'-GAGAATCCTGGCACATCGGGAA-3' |
| CCL5 | 5'-CCCCATATTCCTCGGACACC-3' | 5'-CACACTTGGCGGTTCTTTCG-3' |
| CD1A | 5'-TTGCAGTCAGGGGAGGTTTG-3' | 5'-CTTGAGCCCGTCTGCATTG-3' |
| CD8A | 5'-TTACTGCAACCACAGGAACCG-3' | 5'-GGAAGGACTTGCTCCCTCAA-3' |
| CD14 | 5'-GCCGCTGTGTAGGAAAGAAG-3' | 5'-AGGTTCGGAGAAGTTGCAGA-3' |
| CD56 | 5'-GGAGACCCCATTCCCTCCAT-3' | 5'-ACGAAGCCTTTTCTTCGCTG-3' |
| CD68 | 5'-AGCACAGTGGACATTCTCGG-3' | 5'-TGGGGCAGGAGAAACTTTGC-3' |
| CD86 | 5'-CTGCTCATCTATACACGGTTACC-3' | 5'-GGAAACGTCGTACAGTTCTGTG-3' |
| CD123 | 5'-ACGAAGGAAGATCCAAACCCA-3' | 5'-GCATAGAATAGTCGGCGTCTTTA-3' |
| CD127 | 5'-TGCATGTGACGCCCCTATTC-3' | 5'-GTAGTCCCAAGGCTAAGCAGG-3’ |
| CD163 | 5'-TTTGTCAACTTGAGTCCCTTCAC-3' | 5'-TCCCGCTACACTTGTTTTCAC-3’ |
| CD206 | 5'-CGATCCGACCCTTCCTTGAC-3' | 5'-TGTCTCCGCTTCATGCCATT-3' |
| CD209 | 5'-GCAGTCTTCCAGAAGTAACCGC-3’ | 5'-GCTCTCCTCTGTTCCAATACTGC-3' |
| CSF-1R | 5'-GGGAATCCCAGTGATAGAGCC-3' | 5'-TTGGAAGGTAGCGTTGTTGGT-3' |
| CXCL10 | 5'-AGCAGAGGAACCTCCAGTCT-3' | 5'-CAGACATCTCTTCTCACCCTTC-3' |
| CXCR3 | 5'-CCACCTAGCTGTAGCAGACAC-3' | 5'-AGGGCTCCTGCGTAGAAGTT-3' |
| FOLR2 | 5'-CCACTTCATCCAGGACACCTGT-3' | 5'-CATCCAGGAAGCGTTCTTTGCG-3' |
| GAPDH | 5'-ACAACAGCCTCAAGATCATCAGCA-3' | 5'-TCATGAGTCCTTCCACGATACCAA-3' |
| GZMB | 5'-TACCATTGAGTTGTGCGTGGG-3' | 5'-GCCATTGTTTCGTCCATAGGAGA-3' |
| IFN-γ | 5'-TGAATGTCCAACGCAAAGCA-3' | 5'-CTGGGATGCTCTTCGACCTC-3' |
| IL-10* | N/A | N/A |
| IL-12A* | N/A | N/A |
| ISG15 | 5'-CGCAGATCACCCAGAAGATCG-3' | 5'-TTCGTCGCATTTGTCCACCA-3' |
| LY6E | 5'-CAGCTCGCTGATGTGCTTCT-3' | 5'-CAGACACAGTCACGCAGTAGT-3' |
| MX1 | 5'-GTTTCCGAAGTGGACATCGCA-3' | 5'-CTGCACAGGTTGTTCTCAGC-3' |
| OAS1 | 5'-AGTTGACTGGCGGCTATAAAC-3' | 5'-GTGCTTGACTAGGCGGATGAG-3' |
| NOS2 | 5'-GCTCTACACCTCCAATGTGACC-3' | 5'-CTGCCGAGATTTGAGCCTCATG-3' |
| STAT1 | 5'-TGACGAGGTGTCTCGGATAGT-3' | 5'-GTAGCAGGAGGGAATCACAGA-3' |
| TGF-β1* | N/A | N/A |
| TNF-α | 5'-CACAGTGAAGTGCTGGCAAC-3' | 5'-AGGAAGGCCTAAGGTCCACT-3' |

*: Primer sequences purchased from Bio-Rad were unavailable.

Table S2. Differentially expressed genes in DLE lesional (N=9) and normal skin (N=8) from microarray analysis (>2 fold change, q-value<0.05).

| Gene Symbol | Entrez  ID | Gene Title | Fold Change (DLE vs normal) | d-score | q-value |
| --- | --- | --- | --- | --- | --- |
| CXCL10 | 3627 | chemokine (C-X-C motif) ligand 10 (CXCL10), mRNA. | 66.66 | 31.78 | 0 |
| ISG15 | 9636 | ISG15 ubiquitin-like modifier (ISG15), mRNA. | 57.09 | 33.09 | 0 |
| IFI44L | 10964 | interferon-induced protein 44-like (IFI44L), mRNA. | 35.74 | 24.85 | 0 |
| MX1 | 4599 | myxovirus (influenza virus) resistance 1, interferon-inducible protein p78 (mouse) (MX1), mRNA. | 26.52 | 28.77 | 0 |
| CXCL9 | 4283 | chemokine (C-X-C motif) ligand 9 (CXCL9), mRNA. | 23.09 | 15.45 | 0 |
| OAS2 | 4939 | 2'-5'-oligoadenylate synthetase 2, 69/71kDa (OAS2), transcript variant 2, mRNA./// 2'-5'-oligoadenylate synthetase 2, 69/71kDa (OAS2), transcript variant 1, mRNA. | 22.22 | 23.82 | 0 |
| IFIT1 | 3434 | interferon-induced protein with tetratricopeptide repeats 1 (IFIT1), transcript variant 2, mRNA. | 20.38 | 20.01 | 0 |
| IFIT2 | 3433 | interferon-induced protein with tetratricopeptide repeats 2 (IFIT2), mRNA. | 20.09 | 16.31 | 0 |
| EPSTI1 | 94240 | epithelial stromal interaction 1 (breast) (EPSTI1), transcript variant 2, mRNA. | 19.97 | 27.99 | 0 |
| IFI6 | 2537 | interferon, alpha-inducible protein 6 (IFI6), transcript variant 3, mRNA./// interferon, alpha-inducible protein 6 (IFI6), transcript variant 2, mRNA. | 19.33 | 26.73 | 0 |
| IFI44 | 10561 | interferon-induced protein 44 (IFI44), mRNA. | 18.31 | 25.13 | 0 |
| IGLL1 | 3543 | immunoglobulin lambda-like polypeptide 1 (IGLL1), transcript variant 1, mRNA. | 18.31 | 5.53 | 0 |
| CCL5 | 6352 | chemokine (C-C motif) ligand 5 (CCL5), mRNA. | 16.25 | 17.93 | 0 |
| OASL | 8638 | 2'-5'-oligoadenylate synthetase-like (OASL), transcript variant 2, mRNA. | 16.10 | 19.14 | 0 |
| HERC6 | 55008 | hect domain and RLD 6 (HERC6), transcript variant 1, mRNA. | 16.05 | 24.14 | 0 |
| MX2 | 4600 | myxovirus (influenza virus) resistance 2 (mouse) (MX2), mRNA. | 15.99 | 18.37 | 0 |
| RSAD2 | 91543 | radical S-adenosyl methionine domain containing 2 (RSAD2), mRNA. | 15.87 | 20.22 | 0 |
| IFIT3 | 3437 | interferon-induced protein with tetratricopeptide repeats 3 (IFIT3), mRNA. | 15.78 | 17.25 | 0 |
| LOC642113 | 642113 | PREDICTED: similar to Ig kappa chain V-III region HAH precursor (LOC642113), mRNA. | 15.63 | 4.75 | 0 |
| LOC652493 | 652493 | PREDICTED: similar to Ig kappa chain V-I region HK102 precursor (LOC652493), mRNA. | 15.60 | 5.08 | 0 |
| HERC5 | 51191 | hect domain and RLD 5 (HERC5), mRNA. | 15.11 | 18.22 | 0 |
| LOC647450 | 647450 | PREDICTED: similar to Ig kappa chain V-I region HK101 precursor (LOC647450), mRNA. | 15.02 | 5.37 | 0 |
| LOC647506 | 647506 | PREDICTED: hypothetical LOC647506 (LOC647506), mRNA. | 14.90 | 5.83 | 0 |
| BST2 | 684 | bone marrow stromal cell antigen 2 (BST2), mRNA. | 14.38 | 16.76 | 0 |
| IGFL1 | 374918 | IGF-like family member 1 (IGFL1), mRNA. | 13.96 | 8.74 | 0 |
| LOC100129681 | 100129681 | PREDICTED: similar to NPC-A-7 (LOC100129681), mRNA. | 13.75 | 16.67 | 0 |
| KRT6C | 286887 | keratin 6C (KRT6C), mRNA. | 13.72 | 6.78 | 0 |
| LOC652694 | 652694 | PREDICTED: similar to Ig kappa chain V-I region HK102 precursor (LOC652694), mRNA. | 13.30 | 4.68 | 0 |
| IFI27 | 3429 | interferon, alpha-inducible protein 27 (IFI27), transcript variant 2, mRNA. | 13.23 | 13.80 | 0 |
| XAF1 | 54739 | XIAP associated factor 1 (XAF1), transcript variant 2, mRNA. | 12.57 | 19.64 | 0 |
| GZMB | 3002 | granzyme B (granzyme 2, cytotoxic T-lymphocyte-associated serine esterase 1) (GZMB), mRNA. | 11.30 | 11.48 | 0 |
| OAS3 | 4940 | 2'-5'-oligoadenylate synthetase 3, 100kDa (OAS3), mRNA. | 11.16 | 16.25 | 0 |
| ADAMDEC1 | 27299 | ADAM-like, decysin 1 (ADAMDEC1), mRNA. | 10.58 | 12.09 | 0 |
| PLAC8 | 51316 | placenta-specific 8 (PLAC8), mRNA. | 10.36 | 10.44 | 0 |
| STAT1 | 6772 | signal transducer and activator of transcription 1, 91kDa (STAT1), transcript variant alpha, mRNA./// signal transducer and activator of transcription 1, 91kDa (STAT1), transcript variant beta, mRNA. | 10.33 | 28.93 | 0 |
| MMP9 | 4318 | matrix metallopeptidase 9 (gelatinase B, 92kDa gelatinase, 92kDa type IV collagenase) (MMP9), mRNA. | 9.94 | 10.75 | 0 |
| SAMD9 | 54809 | sterile alpha motif domain containing 9 (SAMD9), mRNA. | 9.80 | 17.76 | 0 |
| IDO1///INDO | 3620 | indoleamine 2,3-dioxygenase 1 (IDO1), mRNA./// indoleamine-pyrrole 2,3 dioxygenase (INDO), mRNA. | 9.44 | 10.94 | 0 |
| ISG20 | 3669 | interferon stimulated exonuclease gene 20kDa (ISG20), mRNA. | 9.39 | 10.58 | 0 |
| C1QB | 713 | complement component 1, q subcomponent, B chain (C1QB), mRNA. | 9.31 | 11.30 | 0 |
| SAMD9L | 219285 | sterile alpha motif domain containing 9-like (SAMD9L), mRNA. | 9.27 | 16.39 | 0 |
| IFITM1 | 8519 | interferon induced transmembrane protein 1 (9-27) (IFITM1), mRNA. | 9.26 | 16.92 | 0 |
| LOC651751 | 651751 | PREDICTED: similar to Ig kappa chain V-II region RPMI 6410 precursor (LOC651751), mRNA. | 8.99 | 4.34 | 0 |
| GBP4 | 115361 | guanylate binding protein 4 (GBP4), mRNA. | 8.91 | 15.12 | 0 |
| ZBP1 | 81030 | Z-DNA binding protein 1 (ZBP1), mRNA. | 8.89 | 17.68 | 0 |
| HS.125087 |  | AGENCOURT_7914287 NIH_MGC_71 cDNA clone IMAGE:6156595 5, mRNA sequence | 8.71 | 19.15 | 0 |
| CCL8 | 6355 | chemokine (C-C motif) ligand 8 (CCL8), mRNA. | 8.63 | 9.36 | 0 |
| IRF8 | 3394 | interferon regulatory factor 8 (IRF8), mRNA. | 8.54 | 11.62 | 0 |
| IRF7 | 3665 | interferon regulatory factor 7 (IRF7), transcript variant b, mRNA. | 8.53 | 13.63 | 0 |
| TRIM22 | 10346 | tripartite motif-containing 22 (TRIM22), mRNA. | 8.45 | 20.71 | 0 |
| IFI35 | 3430 | interferon-induced protein 35 (IFI35), mRNA. | 8.09 | 17.16 | 0 |
| GBP5 | 115362 | guanylate binding protein 5 (GBP5), mRNA. | 7.90 | 12.65 | 0 |
| IFIH1 | 64135 | interferon induced with helicase C domain 1 (IFIH1), mRNA. | 7.84 | 23.25 | 0 |
| IGLL3 | 91353 | immunoglobulin lambda-like polypeptide 3 (IGLL3), mRNA. | 7.76 | 3.91 | 0 |
| LOC729252 | 729252 | PREDICTED: similar to Keratin, type I cytoskeletal 14 (Cytokeratin-14) (CK-14) (Keratin-14) (K14) (LOC729252), mRNA. | 7.58 | 5.70 | 0 |
| C1QC | 714 | complement component 1, q subcomponent, C chain (C1QC), mRNA. | 7.52 | 9.12 | 0 |
| OAS1 | 4938 | 2',5'-oligoadenylate synthetase 1, 40/46kDa (OAS1), transcript variant 2, mRNA./// 2',5'-oligoadenylate synthetase 1, 40/46kDa (OAS1), transcript variant 1, mRNA./// 2',5'-oligoadenylate synthetase 1, 40/46kDa (OAS1), transcript variant 3, mRNA. | 7.36 | 13.84 | 0 |
| CD48 | 962 | CD48 molecule (CD48), mRNA. | 7.18 | 10.30 | 0 |
| PARP14 | 54625 | poly (ADP-ribose) polymerase family, member 14 (PARP14), mRNA. | 7.12 | 18.58 | 0 |
| AIM2 | 9447 | absent in melanoma 2 (AIM2), mRNA. | 7.12 | 12.92 | 0 |
| PRIC285 | 85441 | peroxisomal proliferator-activated receptor A interacting complex 285 (PRIC285), transcript variant 2, mRNA. | 7.10 | 14.44 | 0 |
| SPINK7 | 84651 | serine peptidase inhibitor, Kazal type 7 (putative) (SPINK7), mRNA. | 7.05 | 4.97 | 0 |
| HS.534427 |  | Human mRNA for T-cell specific protein | 6.95 | 8.78 | 0 |
| GBP1 | 2633 | guanylate binding protein 1, interferon-inducible, 67kDa (GBP1), mRNA. | 6.89 | 13.20 | 0 |
| USP18 | 11274 | ubiquitin specific peptidase 18 (USP18), mRNA. | 6.86 | 14.79 | 0 |
| GZMK | 3003 | granzyme K (granzyme 3; tryptase II) (GZMK), mRNA. | 6.84 | 8.72 | 0 |
| CXCL11 | 6373 | chemokine (C-X-C motif) ligand 11 (CXCL11), mRNA. | 6.78 | 6.85 | 0 |
| LAG3 | 3902 | lymphocyte-activation gene 3 (LAG3), mRNA. | 6.70 | 11.46 | 0 |
| CCR7 | 1236 | chemokine (C-C motif) receptor 7 (CCR7), mRNA. | 6.62 | 9.73 | 0 |
| LOC400578 | 400578 | PREDICTED: similar to Keratin, type I cytoskeletal 14 (Cytokeratin-14) (CK-14) (Keratin-14) (K14) (LOC400578), mRNA. | 6.59 | 5.94 | 0 |
| PI3 | 5266 | peptidase inhibitor 3, skin-derived (SKALP) (PI3), mRNA. | 6.58 | 4.71 | 0 |
| UBE2L6 | 9246 | ubiquitin-conjugating enzyme E2L 6 (UBE2L6), transcript variant 1, mRNA. | 6.43 | 16.33 | 0 |
| MGC102966 | 644945 | PREDICTED: similar to Keratin, type I cytoskeletal 16 (Cytokeratin-16) (CK-16) (Keratin-16) (K16) (MGC102966), misc RNA. | 6.41 | 5.97 | 0 |
| GZMA | 3001 | granzyme A (granzyme 1, cytotoxic T-lymphocyte-associated serine esterase 3) (GZMA), mRNA. | 6.39 | 9.95 | 0 |
| NKG7 | 4818 | natural killer cell group 7 sequence (NKG7), mRNA. | 6.35 | 14.70 | 0 |
| DEFB4 | 1673 | defensin, beta 4 (DEFB4), mRNA. | 6.35 | 5.04 | 0 |
| SLC15A3 | 51296 | solute carrier family 15, member 3 (SLC15A3), mRNA. | 6.32 | 14.82 | 0 |
| DEFB103B | 414325 | defensin, beta 103B (DEFB103B), mRNA. | 6.17 | 5.05 | 0 |
| PARP9 | 83666 | poly (ADP-ribose) polymerase family, member 9 (PARP9), mRNA. | 6.15 | 17.36 | 0 |
| CD247 | 919 | CD247 molecule (CD247), transcript variant 2, mRNA./// CD247 molecule (CD247), transcript variant 1, mRNA. | 6.12 | 10.78 | 0 |
| EIF2AK2 | 5610 | eukaryotic translation initiation factor 2-alpha kinase 2 (EIF2AK2), mRNA. | 6.11 | 20.74 | 0 |
| NAPSB | 256236 | napsin B aspartic peptidase pseudogene (NAPSB), non-coding RNA. XR_001413///PREDICTED: napsin B aspartic peptidase pseudogene, transcript variant 3 (NAPSB), misc RNA. | 6.04 | 10.53 | 0 |
| DDX60 | 55601 | DEAD (Asp-Glu-Ala-Asp) box polypeptide 60 (DDX60), mRNA. | 6.01 | 14.78 | 0 |
| CD2 | 914 | CD2 molecule (CD2), mRNA. | 5.99 | 9.95 | 0 |
| TNFSF13B | 10673 | tumor necrosis factor (ligand) superfamily, member 13b (TNFSF13B), transcript variant 1, mRNA. | 5.93 | 12.84 | 0 |
| HLA-F | 3134 | major histocompatibility complex, class I, F (HLA-F), transcript variant 1, mRNA./// major histocompatibility complex, class I, F (HLA-F), mRNA. | 5.90 | 9.06 | 0 |
| IGJ | 3512 | immunoglobulin J polypeptide, linker protein for immunoglobulin alpha and mu polypeptides (IGJ), mRNA. | 5.79 | 5.13 | 0 |
| C10ORF99 | 387695 | chromosome 10 open reading frame 99 (C10orf99), mRNA. | 5.72 | 5.61 | 0 |
| CFB | 629 | complement factor B (CFB), mRNA. | 5.70 | 12.68 | 0 |
| PARP12 | 64761 | poly (ADP-ribose) polymerase family, member 12 (PARP12), mRNA. | 5.56 | 16.71 | 0 |
| RARRES3 | 5920 | retinoic acid receptor responder (tazarotene induced) 3 (RARRES3), mRNA. | 5.51 | 14.27 | 0 |
| LOC649923 | 649923 | PREDICTED: similar to Ig gamma-2 chain C region (LOC649923), mRNA. | 5.45 | 2.77 | 0.0007 |
| LTB | 4050 | lymphotoxin beta (TNF superfamily, member 3) (LTB), transcript variant 1, mRNA. | 5.42 | 8.66 | 0 |
| SP110 | 3431 | SP110 nuclear body protein (SP110), transcript variant c, mRNA./// SP110 nuclear body protein (SP110), transcript variant b, mRNA. | 5.40 | 17.00 | 0 |
| IL7R | 3575 | interleukin 7 receptor (IL7R), mRNA.///PREDICTED: interleukin 7 receptor (IL7R), mRNA. | 5.40 | 6.87 | 0 |
| GVIN1 | 387751 | PREDICTED: GTPase, very large interferon inducible 1 (GVIN1), mRNA. | 5.38 | 11.16 | 0 |
| LCE3D | 84648 | late cornified envelope 3D (LCE3D), mRNA. | 5.34 | 4.84 | 0 |
| CD3D | 915 | CD3d molecule, delta (CD3-TCR complex) (CD3D), transcript variant 1, mRNA./// CD3d molecule, delta (CD3-TCR complex) (CD3D), transcript variant 2, mRNA. | 5.33 | 8.33 | 0 |
| GIMAP4 | 55303 | GTPase, IMAP family member 4 (GIMAP4), mRNA. | 5.28 | 9.33 | 0 |
| HS.554324 |  | full-length cDNA clone CS0DI056YK21 of Placenta Cot 25-normalized of (human) | 5.27 | 8.27 | 0 |
| LGALS3BP | 3959 | lectin, galactoside-binding, soluble, 3 binding protein (LGALS3BP), mRNA. | 5.23 | 8.62 | 0 |
| TAP1 | 6890 | transporter 1, ATP-binding cassette, sub-family B (MDR/TAP) (TAP1), mRNA. | 5.23 | 10.98 | 0 |
| RTP4 | 64108 | receptor (chemosensory) transporter protein 4 (RTP4), mRNA. | 5.23 | 12.17 | 0 |
| SPRR2A | 6700 | small proline-rich protein 2A (SPRR2A), mRNA. | 5.13 | 3.48 | 0 |
| CD8A | 925 | CD8a molecule (CD8A), transcript variant 2, mRNA./// CD8a molecule (CD8A), transcript variant 1, mRNA. | 5.10 | 9.02 | 0 |
| STAT2 | 6773 | signal transducer and activator of transcription 2, 113kDa (STAT2), mRNA. | 5.04 | 12.42 | 0 |
| IFI30 | 10437 | interferon, gamma-inducible protein 30 (IFI30), mRNA. | 5.02 | 10.62 | 0 |
| TDO2 | 6999 | tryptophan 2,3-dioxygenase (TDO2), mRNA. | 4.97 | 8.28 | 0 |
| HLA-H | 3136 | major histocompatibility complex, class I, H (pseudogene) (HLA-H), non-coding RNA. | 4.93 | 7.44 | 0 |
| DDX60L | 91351 | DEAD (Asp-Glu-Ala-Asp) box polypeptide 60-like (DDX60L), mRNA. | 4.84 | 18.95 | 0 |
| RAC2 | 5880 | ras-related C3 botulinum toxin substrate 2 (rho family, small GTP binding protein Rac2) (RAC2), mRNA. | 4.82 | 9.66 | 0 |
| HSH2D | 84941 | hematopoietic SH2 domain containing (HSH2D), mRNA. | 4.81 | 12.48 | 0 |
| LY6E | 4061 | lymphocyte antigen 6 complex, locus E (LY6E), mRNA. | 4.76 | 8.41 | 0 |
| LOC728454 | 728454 | PREDICTED: similar to Beta-defensin 2 precursor (BD-2) (hBD-2) (Defensin, beta 2) (Skin-antimicrobial peptide 1) (SAP1) (LOC728454), mRNA. | 4.70 | 4.85 | 0 |
| CXCL13 | 10563 | chemokine (C-X-C motif) ligand 13 (B-cell chemoattractant) (CXCL13), mRNA. | 4.66 | 6.24 | 0 |
| HLA-DRB6 | 3128 | major histocompatibility complex, class II, DR beta 6 (pseudogene) (HLA-DRB6), non-coding RNA. | 4.65 | 5.59 | 0 |
| LAMP3 | 27074 | lysosomal-associated membrane protein 3 (LAMP3), mRNA. | 4.65 | 11.54 | 0 |
| PTPRCAP | 5790 | protein tyrosine phosphatase, receptor type, C-associated protein (PTPRCAP), mRNA. | 4.64 | 9.66 | 0 |
| KRT16 | 3868 | keratin 16 (focal non-epidermolytic palmoplantar keratoderma) (KRT16), mRNA. | 4.63 | 6.16 | 0 |
| HCST | 10870 | hematopoietic cell signal transducer (HCST), transcript variant 2, mRNA./// hematopoietic cell signal transducer (HCST), transcript variant 1, mRNA. | 4.60 | 9.71 | 0 |
| HLA-B | 3106 | major histocompatibility complex, class I, B (HLA-B), mRNA. | 4.58 | 7.94 | 0 |
| CORO1A | 11151 | coronin, actin binding protein, 1A (CORO1A), mRNA. | 4.57 | 9.67 | 0 |
| WARS | 7453 | tryptophanyl-tRNA synthetase (WARS), transcript variant 2, mRNA./// tryptophanyl-tRNA synthetase (WARS), transcript variant 1, mRNA. | 4.47 | 8.29 | 0 |
| UBA7 | 7318 | ubiquitin-like modifier activating enzyme 7 (UBA7), mRNA. | 4.42 | 11.11 | 0 |
| UBD | 10537 | ubiquitin D (UBD), mRNA. | 4.39 | 6.20 | 0 |
| HLA-G | 3135 | HLA-G histocompatibility antigen, class I, G (HLA-G), mRNA. | 4.39 | 8.45 | 0 |
| C19ORF66 | 55337 | chromosome 19 open reading frame 66 (C19orf66), mRNA. | 4.38 | 15.25 | 0 |
| TYMP///ECGF1 | 1890 | thymidine phosphorylase (TYMP), transcript variant 1, mRNA./// endothelial cell growth factor 1 (platelet-derived) (ECGF1), mRNA./// thymidine phosphorylase (TYMP), transcript variant 3, mRNA. | 4.36 | 9.79 | 0 |
| IFITM3 | 10410 | interferon induced transmembrane protein 3 (1-8U) (IFITM3), mRNA. | 4.36 | 12.38 | 0 |
| TLR7 | 51284 | toll-like receptor 7 (TLR7), mRNA. | 4.33 | 8.85 | 0 |
| TMEM140 | 55281 | transmembrane protein 140 (TMEM140), mRNA. | 4.32 | 10.57 | 0 |
| HS.546375 |  | T cell receptor alpha locus, mRNA (cDNA clone MGC:88342 IMAGE:30352166), complete cds | 4.32 | 7.79 | 0 |
| CD74 | 972 | CD74 molecule, major histocompatibility complex, class II invariant chain (CD74), transcript variant 2, mRNA./// CD74 molecule, major histocompatibility complex, class II invariant chain (CD74), transcript variant 1, mRNA. | 4.31 | 8.53 | 0 |
| DHX58 | 79132 | DEXH (Asp-Glu-X-His) box polypeptide 58 (DHX58), mRNA. | 4.29 | 14.12 | 0 |
| SPRR2G | 6706 | small proline-rich protein 2G (SPRR2G), mRNA. | 4.23 | 5.44 | 0 |
| FAIM3 | 9214 | Fas apoptotic inhibitory molecule 3 (FAIM3), mRNA. | 4.17 | 7.94 | 0 |
| SPOCK2 | 9806 | sparc/osteonectin, cwcv and kazal-like domains proteoglycan (testican) 2 (SPOCK2), mRNA. | 4.17 | 8.46 | 0 |
| GIMAP7 | 168537 | GTPase, IMAP family member 7 (GIMAP7), mRNA. | 4.15 | 6.70 | 0 |
| RNASE6 | 6039 | ribonuclease, RNase A family, k6 (RNASE6), mRNA. | 4.14 | 7.23 | 0 |
| TBC1D10C | 374403 | TBC1 domain family, member 10C (TBC1D10C), mRNA. | 4.13 | 9.10 | 0 |
| LYZ | 4069 | lysozyme (renal amyloidosis) (LYZ), mRNA. | 4.13 | 5.45 | 0 |
| CECR1 | 51816 | cat eye syndrome chromosome region, candidate 1 (CECR1), transcript variant 2, mRNA. | 4.13 | 8.88 | 0 |
| SLAMF6 | 114836 | SLAM family member 6 (SLAMF6), mRNA. | 4.10 | 9.19 | 0 |
| RPTN | 126638 | PREDICTED: repetin (RPTN), mRNA. | 4.09 | 4.82 | 0 |
| GIMAP5 | 55340 | GTPase, IMAP family member 5 (GIMAP5), mRNA. | 4.05 | 8.40 | 0 |
| PSMB9 | 5698 | proteasome (prosome, macropain) subunit, beta type, 9 (large multifunctional peptidase 2) (PSMB9), transcript variant 1, mRNA. | 4.03 | 9.67 | 0 |
| MCOLN2 | 255231 | mucolipin 2 (MCOLN2), mRNA. | 4.03 | 8.53 | 0 |
| FPR3 | 2359 | formyl peptide receptor 3 (FPR3), mRNA. | 4.02 | 11.02 | 0 |
| IL2RB | 3560 | interleukin 2 receptor, beta (IL2RB), mRNA. | 3.99 | 6.93 | 0 |
| SPRR2F | 6705 | small proline-rich protein 2F (SPRR2F), mRNA. | 3.95 | 3.89 | 0 |
| LOC643384 | 643384 | PREDICTED: hypothetical LOC643384 (LOC643384), mRNA.///PREDICTED: similar to hCG2036843 (LOC643384), mRNA. | 3.95 | 11.45 | 0 |
| PARP10 | 84875 | poly (ADP-ribose) polymerase family, member 10 (PARP10), mRNA.///PREDICTED: poly (ADP-ribose) polymerase family, member 10 (PARP10), mRNA. | 3.88 | 11.86 | 0 |
| HCG4 | 54435 | HLA complex group 4 (HCG4), non-coding RNA. | 3.86 | 7.49 | 0 |
| LGALS9 | 3965 | lectin, galactoside-binding, soluble, 9 (LGALS9), transcript variant 1, mRNA. | 3.84 | 12.86 | 0 |
| CD52 | 1043 | CD52 molecule (CD52), mRNA. | 3.84 | 5.86 | 0 |
| HCLS1 | 3059 | hematopoietic cell-specific Lyn substrate 1 (HCLS1), mRNA. | 3.84 | 7.33 | 0 |
| CD6 | 923 | CD6 molecule (CD6), mRNA. | 3.81 | 8.40 | 0 |
| PLEK | 5341 | pleckstrin (PLEK), mRNA. | 3.79 | 6.53 | 0 |
| LCE3E | 353145 | late cornified envelope 3E (LCE3E), mRNA. | 3.79 | 5.72 | 0 |
| HLA-DMB | 3109 | major histocompatibility complex, class II, DM beta (HLA-DMB), mRNA. | 3.78 | 8.30 | 0 |
| DDX58 | 23586 | DEAD (Asp-Glu-Ala-Asp) box polypeptide 58 (DDX58), mRNA. | 3.77 | 11.19 | 0 |
| PVRIG | 79037 | poliovirus receptor related immunoglobulin domain containing (PVRIG), mRNA. | 3.76 | 9.35 | 0 |
| TNFSF10 | 8743 | tumor necrosis factor (ligand) superfamily, member 10 (TNFSF10), mRNA. | 3.76 | 8.65 | 0 |
| HS.72010 |  | RST24587 Athersys RAGE Library cDNA, mRNA sequence | 3.74 | 10.69 | 0 |
| HCP5 | 10866 | HLA complex P5 (HCP5), mRNA. | 3.72 | 6.14 | 0 |
| PSMB10 | 5699 | proteasome (prosome, macropain) subunit, beta type, 10 (PSMB10), mRNA. | 3.70 | 14.61 | 0 |
| SPRR2B | 6701 | small proline-rich protein 2B (SPRR2B), mRNA. | 3.68 | 4.34 | 0 |
| EOMES | 8320 | eomesodermin homolog (Xenopus laevis) (EOMES), mRNA. | 3.67 | 8.08 | 0 |
| APOBEC3G | 60489 | apolipoprotein B mRNA editing enzyme, catalytic polypeptide-like 3G (APOBEC3G), mRNA. | 3.67 | 11.35 | 0 |
| IRF9 | 10379 | interferon regulatory factor 9 (IRF9), mRNA. | 3.64 | 13.35 | 0 |
| SELL | 6402 | selectin L (SELL), mRNA. | 3.64 | 7.55 | 0 |
| AKR1B10 | 57016 | aldo-keto reductase family 1, member B10 (aldose reductase) (AKR1B10), mRNA. | 3.63 | 5.23 | 0 |
| ZNFX1 | 57169 | zinc finger, NFX1-type containing 1 (ZNFX1), mRNA. | 3.58 | 10.80 | 0 |
| CD96 | 10225 | CD96 molecule (CD96), transcript variant 1, mRNA./// CD96 molecule (CD96), transcript variant 2, mRNA. | 3.55 | 8.22 | 0 |
| LOC606724 | 606724 | coronin, actin binding protein, 1A pseudogene (LOC606724), non-coding RNA. | 3.55 | 8.65 | 0 |
| SLPI | 6590 | secretory leukocyte peptidase inhibitor (SLPI), mRNA. | 3.53 | 4.58 | 0 |
| LOC730415 | 730415 | PREDICTED: hypothetical LOC730415, transcript variant 2 (LOC730415), mRNA. | 3.51 | 8.23 | 0 |
| SH2D1A | 4068 | SH2 domain protein 1A, Duncan's disease (lymphoproliferative syndrome) (SH2D1A), mRNA. | 3.49 | 7.90 | 0 |
| IFITM2 | 10581 | interferon induced transmembrane protein 2 (1-8D) (IFITM2), mRNA. | 3.49 | 9.69 | 0 |
| NAPSA | 9476 | napsin A aspartic peptidase (NAPSA), mRNA. | 3.48 | 7.70 | 0 |
| LPXN | 9404 | leupaxin (LPXN), mRNA. | 3.46 | 8.91 | 0 |
| FCER1G | 2207 | Fc fragment of IgE, high affinity I, receptor for; gamma polypeptide (FCER1G), mRNA. | 3.45 | 7.49 | 0 |
| IL4I1 | 259307 | interleukin 4 induced 1 (IL4I1), transcript variant 2, mRNA. | 3.44 | 10.63 | 0 |
| MS4A6A | 64231 | membrane-spanning 4-domains, subfamily A, member 6A (MS4A6A), transcript variant 2, mRNA./// membrane-spanning 4-domains, subfamily A, member 6A (MS4A6A), transcript variant 3, mRNA. | 3.43 | 5.80 | 0 |
| FGD3 | 89846 | FYVE, RhoGEF and PH domain containing 3 (FGD3), transcript variant 2, mRNA. | 3.42 | 9.11 | 0 |
| IL10RA | 3587 | interleukin 10 receptor, alpha (IL10RA), mRNA. | 3.42 | 8.71 | 0 |
| FCRLA | 84824 | Fc receptor-like A (FCRLA), mRNA. | 3.42 | 5.55 | 0 |
| HLA-A29.1///LOC649853 | 649853 | major histocompatibility complex class I HLA-A29.1 (HLA-A29.1), mRNA. XM_001132736 XM_001132742 XM_001132747 XM_001132750 XM_001132753 XM_001132757 XM_001132760 XM_001132769 XM_001132772 XM_001132776 XM_001132779 XM_001132781 XM_001132783 XM_001132785 XM_001132794///PREDICTED: similar to HLA class I histocompatibility antigen, A-29 alpha chain precursor (MHC class I antigen A*29) (Aw-19), transcript variant 1 (LOC649853), mRNA. | 3.41 | 2.62 | 0.0007 |
| MAP4K1 | 11184 | mitogen-activated protein kinase kinase kinase kinase 1 (MAP4K1), transcript variant 1, mRNA. | 3.41 | 9.08 | 0 |
| TYROBP | 7305 | TYRO protein tyrosine kinase binding protein (TYROBP), transcript variant 1, mRNA. | 3.40 | 7.92 | 0 |
| NCKAP1L | 3071 | NCK-associated protein 1-like (NCKAP1L), mRNA. | 3.40 | 9.14 | 0 |
| SPRR1B | 6699 | small proline-rich protein 1B (cornifin) (SPRR1B), mRNA. | 3.37 | 4.09 | 0 |
| ITGB2 | 3689 | integrin, beta 2 (antigen CD18 (p95), lymphocyte function-associated antigen 1; macrophage antigen 1 (mac-1) beta subunit) (ITGB2), mRNA./// integrin, beta 2 (complement component 3 receptor 3 and 4 subunit) (ITGB2), mRNA. | 3.37 | 7.46 | 0 |
| LBA1 | 9881 | lupus brain antigen 1 (LBA1), mRNA. | 3.37 | 11.07 | 0 |
| ICOS | 29851 | inducible T-cell co-stimulator (ICOS), mRNA. | 3.36 | 6.24 | 0 |
| HLA-DRB4 | 3126 | major histocompatibility complex, class II, DR beta 4 (HLA-DRB4), mRNA. | 3.36 | 4.83 | 0 |
| FAM26F | 441168 | family with sequence similarity 26, member F (FAM26F), mRNA. | 3.36 | 10.56 | 0 |
| APOL3 | 80833 | apolipoprotein L, 3 (APOL3), transcript variant beta/a, mRNA. | 3.34 | 18.81 | 0 |
| CHN1 | 1123 | chimerin (chimaerin) 1 (CHN1), transcript variant 2, mRNA. | 3.34 | 14.27 | 0 |
| CD79A | 973 | CD79a molecule, immunoglobulin-associated alpha (CD79A), transcript variant 2, mRNA. | 3.33 | 5.24 | 0 |
| STAT4 | 6775 | signal transducer and activator of transcription 4 (STAT4), mRNA. | 3.33 | 7.77 | 0 |
| HLA-DMA | 3108 | major histocompatibility complex, class II, DM alpha (HLA-DMA), mRNA. | 3.32 | 7.89 | 0 |
| WAS | 7454 | Wiskott-Aldrich syndrome (eczema-thrombocytopenia) (WAS), mRNA. | 3.32 | 7.28 | 0 |
| CYBA | 1535 | cytochrome b-245, alpha polypeptide (CYBA), mRNA. | 3.32 | 8.53 | 0 |
| CD38 | 952 | CD38 molecule (CD38), mRNA. | 3.32 | 8.52 | 0 |
| LAP3 | 51056 | leucine aminopeptidase 3 (LAP3), mRNA. | 3.31 | 11.21 | 0 |
| BTN3A1 | 11119 | butyrophilin, subfamily 3, member A1 (BTN3A1), mRNA. | 3.31 | 10.63 | 0 |
| CD163 | 9332 | CD163 molecule (CD163), transcript variant 2, mRNA./// CD163 molecule (CD163), transcript variant 1, mRNA. | 3.30 | 7.46 | 0 |
| MDK | 4192 | midkine (neurite growth-promoting factor 2) (MDK), transcript variant 1, mRNA. | 3.29 | 7.90 | 0 |
| RGS1 | 5996 | regulator of G-protein signaling 1 (RGS1), mRNA. | 3.28 | 3.77 | 0 |
| PRF1 | 5551 | perforin 1 (pore forming protein) (PRF1), transcript variant 1, mRNA. | 3.27 | 8.67 | 0 |
| CD69 | 969 | CD69 molecule (CD69), mRNA. | 3.26 | 4.50 | 0 |
| IRF1 | 3659 | interferon regulatory factor 1 (IRF1), mRNA. | 3.26 | 6.61 | 0 |
| HLA-E | 3133 | major histocompatibility complex, class I, E (HLA-E), mRNA. | 3.26 | 9.98 | 0 |
| PSMB8 | 5696 | proteasome (prosome, macropain) subunit, beta type, 8 (large multifunctional peptidase 7) (PSMB8), transcript variant 2, mRNA./// proteasome (prosome, macropain) subunit, beta type, 8 (large multifunctional peptidase 7) (PSMB8), transcript variant 1, mRNA. | 3.25 | 12.35 | 0 |
| LYN | 4067 | v-yes-1 Yamaguchi sarcoma viral related oncogene homolog (LYN), mRNA. | 3.24 | 7.62 | 0 |
| LAPTM5 | 7805 | lysosomal multispanning membrane protein 5 (LAPTM5), mRNA. | 3.23 | 7.58 | 0 |
| ADAR | 103 | adenosine deaminase, RNA-specific (ADAR), transcript variant 1, mRNA./// adenosine deaminase, RNA-specific (ADAR), transcript variant 2, mRNA. | 3.22 | 9.21 | 0 |
| FGD2 | 221472 | FYVE, RhoGEF and PH domain containing 2 (FGD2), mRNA. | 3.21 | 9.80 | 0 |
| KRT6B | 3854 | keratin 6B (KRT6B), mRNA. | 3.19 | 7.02 | 0 |
| LCE2A | 353139 | late cornified envelope 2A (LCE2A), mRNA. | 3.17 | 3.76 | 0 |
| CD53 | 963 | CD53 molecule (CD53), transcript variant 2, mRNA. | 3.16 | 7.59 | 0 |
| GIMAP8 | 155038 | GTPase, IMAP family member 8 (GIMAP8), mRNA. | 3.16 | 8.05 | 0 |
| AKNA | 80709 | AT-hook transcription factor (AKNA), mRNA. | 3.16 | 8.78 | 0 |
| ITK | 3702 | IL2-inducible T-cell kinase (ITK), mRNA. | 3.16 | 7.27 | 0 |
| PIM2 | 11040 | pim-2 oncogene (PIM2), mRNA. | 3.15 | 6.75 | 0 |
| ITGAL | 3683 | integrin, alpha L (antigen CD11A (p180), lymphocyte function-associated antigen 1; alpha polypeptide) (ITGAL), mRNA. | 3.15 | 8.23 | 0 |
| GSDMD | 79792 | gasdermin D (GSDMD), mRNA. | 3.14 | 14.14 | 0 |
| DOCK2 | 1794 | dedicator of cytokinesis 2 (DOCK2), mRNA. | 3.13 | 7.49 | 0 |
| TRIM21 | 6737 | tripartite motif-containing 21 (TRIM21), mRNA. | 3.13 | 12.64 | 0 |
| CD7 | 924 | CD7 molecule (CD7), mRNA. | 3.12 | 8.62 | 0 |
| RASAL3 | 64926 | RAS protein activator like 3 (RASAL3), mRNA. | 3.11 | 9.99 | 0 |
| SPRR2C | 6702 | small proline-rich protein 2C (pseudogene) (SPRR2C), non-coding RNA. | 3.09 | 3.71 | 0 |
| HESX1 | 8820 | HESX homeobox 1 (HESX1), mRNA. | 3.07 | 7.18 | 0 |
| PARVG | 64098 | parvin, gamma (PARVG), mRNA. | 3.07 | 8.87 | 0 |
| HLA-DRB3 | 3125 | major histocompatibility complex, class II, DR beta 3 (HLA-DRB3), mRNA. | 3.07 | 6.87 | 0 |
| CD27 | 939 | CD27 molecule (CD27), mRNA. | 3.05 | 7.12 | 0 |
| LOC649210 | 649210 | PREDICTED: similar to Ig lambda chain V region 4A precursor (LOC649210), mRNA. | 3.05 | 3.73 | 0 |
| GPSM3 | 63940 | G-protein signaling modulator 3 (AGS3-like, C. elegans) (GPSM3), mRNA. | 3.05 | 7.51 | 0 |
| ITGB7 | 3695 | integrin, beta 7 (ITGB7), mRNA. | 3.04 | 10.28 | 0 |
| SIGLEC14 | 100049587 | sialic acid binding Ig-like lectin 14 (SIGLEC14), mRNA. | 3.03 | 6.43 | 0 |
| LYSMD2 | 256586 | LysM, putative peptidoglycan-binding, domain containing 2 (LYSMD2), mRNA. | 3.03 | 9.85 | 0 |
| CDC25B | 994 | cell division cycle 25 homolog B (S. pombe) (CDC25B), transcript variant 2, mRNA. | 3.03 | 9.76 | 0 |
| ARHGAP4 | 393 | Rho GTPase activating protein 4 (ARHGAP4), mRNA. | 3.03 | 9.90 | 0 |
| CD37 | 951 | CD37 antigen (CD37), mRNA./// CD37 molecule (CD37), transcript variant 1, mRNA. | 3.03 | 7.88 | 0 |
| HLA-A | 3105 | major histocompatibility complex, class I, A (HLA-A), mRNA. | 3.01 | 6.49 | 0 |
| C1QA | 712 | complement component 1, q subcomponent, alpha polypeptide (C1QA), mRNA. | 3.01 | 8.64 | 0 |
| SLC7A7 | 9056 | solute carrier family 7 (cationic amino acid transporter, y+ system), member 7 (SLC7A7), mRNA. | 3.01 | 9.16 | 0 |
| LY86 | 9450 | lymphocyte antigen 86 (LY86), mRNA. | 3.01 | 7.88 | 0 |
| MMP1 | 4312 | matrix metallopeptidase 1 (interstitial collagenase) (MMP1), mRNA. | 3.00 | 2.35 | 0.002 |
| PTPN6 | 5777 | protein tyrosine phosphatase, non-receptor type 6 (PTPN6), transcript variant 2, mRNA. | 3.00 | 7.41 | 0 |
| LGMN | 5641 | legumain (LGMN), transcript variant 2, mRNA. | 2.99 | 7.72 | 0 |
| LOC389386 | 389386 | PREDICTED: misc_RNA (LOC389386), partial miscRNA. | 2.99 | 10.04 | 0 |
| SASH3 | 54440 | SAM and SH3 domain containing 3 (SASH3), mRNA. | 2.98 | 7.77 | 0 |
| FBXO6 | 26270 | F-box protein 6 (FBXO6), mRNA. | 2.97 | 10.50 | 0 |
| CYBB | 1536 | cytochrome b-245, beta polypeptide (chronic granulomatous disease) (CYBB), mRNA. | 2.96 | 6.94 | 0 |
| HAVCR2 | 84868 | hepatitis A virus cellular receptor 2 (HAVCR2), mRNA. | 2.96 | 9.71 | 0 |
| LCP1 | 3936 | lymphocyte cytosolic protein 1 (L-plastin) (LCP1), mRNA. | 2.95 | 6.87 | 0 |
| LGALS2 | 3957 | lectin, galactoside-binding, soluble, 2 (LGALS2), mRNA. | 2.95 | 6.22 | 0 |
| SAMD3 | 154075 | sterile alpha motif domain containing 3 (SAMD3), transcript variant 1, mRNA. | 2.95 | 7.66 | 0 |
| KIAA1618 | 57714 | KIAA1618 (KIAA1618), mRNA.///PREDICTED: KIAA1618 (KIAA1618), mRNA. | 2.94 | 14.11 | 0 |
| LOC644936 | 644936 | cytoplasmic beta-actin pseudogene (LOC644936), non-coding RNA. | 2.93 | 3.31 | 0 |
| HS.572649 |  | cDNA FLJ46527 fis, clone THYMU3034853 | 2.93 | 7.12 | 0 |
| CD86 | 942 | CD86 antigen (CD28 antigen ligand 2, B7-2 antigen) (CD86), transcript variant 1, mRNA./// CD86 molecule (CD86), transcript variant 2, mRNA. | 2.92 | 9.04 | 0 |
| BTN3A3 | 10384 | butyrophilin, subfamily 3, member A3 (BTN3A3), transcript variant 2, mRNA. | 2.92 | 8.89 | 0 |
| DNASE1L3 | 1776 | deoxyribonuclease I-like 3 (DNASE1L3), mRNA. | 2.92 | 6.73 | 0 |
| LOC649143 | 649143 | PREDICTED: similar to HLA class II histocompatibility antigen, DRB1-9 beta chain precursor (MHC class I antigen DRB1*9) (DR-9) (DR9), transcript variant 2 (LOC649143), mRNA. | 2.91 | 2.16 | 0.003 |
| GIMAP6 | 474344 | GTPase, IMAP family member 6 (GIMAP6), transcript variant 3, mRNA. | 2.91 | 7.51 | 0 |
| FERMT3 | 83706 | fermitin family homolog 3 (Drosophila) (FERMT3), transcript variant URP2SF, mRNA. | 2.88 | 8.18 | 0 |
| EVI2B | 2124 | ecotropic viral integration site 2B (EVI2B), mRNA. | 2.88 | 5.99 | 0 |
| ARHGAP15 | 55843 | Rho GTPase activating protein 15 (ARHGAP15), mRNA. | 2.87 | 7.14 | 0 |
| UCP2 | 7351 | uncoupling protein 2 (mitochondrial, proton carrier) (UCP2), nuclear gene encoding mitochondrial protein, mRNA. | 2.87 | 5.81 | 0 |
| LAX1 | 54900 | lymphocyte transmembrane adaptor 1 (LAX1), mRNA. | 2.87 | 6.77 | 0 |
| LOC400759 | 400759 | similar to Interferon-induced guanylate-binding protein 1 (GTP-binding protein 1) (Guanine nucleotide-binding protein 1) (HuGBP-1) (LOC400759) on chromosome 1. | 2.85 | 11.52 | 0 |
| PSME2 | 5721 | proteasome (prosome, macropain) activator subunit 2 (PA28 beta) (PSME2), mRNA. | 2.84 | 14.98 | 0 |
| SEL1L3 | 23231 | sel-1 suppressor of lin-12-like 3 (C. elegans) (SEL1L3), mRNA. | 2.83 | 7.12 | 0 |
| HLA-DPA1 | 3113 | major histocompatibility complex, class II, DP alpha 1 (HLA-DPA1), mRNA. | 2.83 | 6.15 | 0 |
| LILRB4 | 11006 | leukocyte immunoglobulin-like receptor, subfamily B (with TM and ITIM domains), member 4 (LILRB4), transcript variant 2, mRNA. | 2.81 | 7.97 | 0 |
| DOCK10 | 55619 | dedicator of cytokinesis 10 (DOCK10), mRNA. | 2.81 | 7.27 | 0 |
| SHISA5 | 51246 | shisa homolog 5 (Xenopus laevis) (SHISA5), mRNA. | 2.81 | 9.03 | 0 |
| IL1F9 | 56300 | interleukin 1 family, member 9 (IL1F9), mRNA. | 2.81 | 3.79 | 0 |
| CYTH4///PSCD4 | 27128 | cytohesin 4 (CYTH4), mRNA./// pleckstrin homology, Sec7 and coiled-coil domains 4 (PSCD4), mRNA. | 2.80 | 6.57 | 0 |
| PHF11 | 51131 | PHD finger protein 11 (PHF11), transcript variant 1, mRNA. | 2.80 | 11.32 | 0 |
| MICB | 4277 | MHC class I polypeptide-related sequence B (MICB), mRNA. | 2.79 | 12.43 | 0 |
| ADAM19 | 8728 | ADAM metallopeptidase domain 19 (meltrin beta) (ADAM19), mRNA. | 2.79 | 5.69 | 0 |
| TDRD7 | 23424 | tudor domain containing 7 (TDRD7), mRNA. | 2.78 | 10.99 | 0 |
| FCRL3 | 115352 | Fc receptor-like 3 (FCRL3), transcript variant 2, mRNA. | 2.78 | 5.87 | 0 |
| CASP1 | 834 | caspase 1, apoptosis-related cysteine peptidase (interleukin 1, beta, convertase) (CASP1), transcript variant delta, mRNA. | 2.77 | 7.59 | 0 |
| WFDC12 | 128488 | WAP four-disulfide core domain 12 (WFDC12), mRNA. | 2.76 | 4.14 | 0 |
| GIMAP2 | 26157 | GTPase, IMAP family member 2 (GIMAP2), mRNA. | 2.76 | 5.61 | 0 |
| UNC93B1 | 81622 | unc-93 homolog B1 (C. elegans) (UNC93B1), mRNA. | 2.75 | 11.08 | 0 |
| SCO2 | 9997 | SCO cytochrome oxidase deficient homolog 2 (yeast) (SCO2), nuclear gene encoding mitochondrial protein, mRNA. | 2.74 | 6.71 | 0 |
| ALOX5AP | 241 | arachidonate 5-lipoxygenase-activating protein (ALOX5AP), mRNA. | 2.73 | 4.78 | 0 |
| TMEM149 | 79713 | transmembrane protein 149 (TMEM149), mRNA. | 2.73 | 6.33 | 0 |
| PACAP///MGC29506 | 51237 | proapoptotic caspase adaptor protein (PACAP), mRNA./// hypothetical protein MGC29506 (MGC29506), mRNA. | 2.72 | 5.69 | 0 |
| FAM113B | 91523 | family with sequence similarity 113, member B (FAM113B), mRNA. | 2.71 | 7.43 | 0 |
| SEMA4D | 10507 | sema domain, immunoglobulin domain (Ig), transmembrane domain (TM) and short cytoplasmic domain, (semaphorin) 4D (SEMA4D), mRNA. | 2.71 | 8.25 | 0 |
| CTHRC1 | 115908 | collagen triple helix repeat containing 1 (CTHRC1), mRNA. | 2.70 | 6.62 | 0 |
| IL18BP | 10068 | interleukin 18 binding protein (IL18BP), transcript variant D, mRNA./// interleukin 18 binding protein (IL18BP), transcript variant A, mRNA./// interleukin 18 binding protein (IL18BP), transcript variant C, mRNA. | 2.70 | 10.51 | 0 |
| P2RY8 | 286530 | purinergic receptor P2Y, G-protein coupled, 8 (P2RY8), mRNA. | 2.69 | 6.37 | 0 |
| MYO1G | 64005 | myosin IG (MYO1G), mRNA. | 2.68 | 7.78 | 0 |
| C1ORF162 | 128346 | chromosome 1 open reading frame 162 (C1orf162), mRNA. | 2.68 | 9.52 | 0 |
| NT5E | 4907 | 5'-nucleotidase, ecto (CD73) (NT5E), mRNA. | 2.68 | 9.51 | 0 |
| LYL1 | 4066 | lymphoblastic leukemia derived sequence 1 (LYL1), mRNA. | 2.67 | 7.16 | 0 |
| IL1F5 | 26525 | interleukin 1 family, member 5 (delta) (IL1F5), transcript variant 2, mRNA. | 2.66 | 5.01 | 0 |
| HLA-DRB1 | 3123 | major histocompatibility complex, class II, DR beta 1 (HLA-DRB1), mRNA. | 2.66 | 1.39 | 0.008 |
| TAP2 | 6891 | transporter 2, ATP-binding cassette, sub-family B (MDR/TAP) (TAP2), transcript variant 1, mRNA./// transporter 2, ATP-binding cassette, sub-family B (MDR/TAP) (TAP2), transcript variant 2, mRNA. | 2.66 | 9.06 | 0 |
| VCAM1 | 7412 | vascular cell adhesion molecule 1 (VCAM1), transcript variant 2, mRNA./// vascular cell adhesion molecule 1 (VCAM1), transcript variant 1, mRNA. | 2.66 | 7.01 | 0 |
| GIMAP1 | 170575 | GTPase, IMAP family member 1 (GIMAP1), mRNA. | 2.65 | 7.36 | 0 |
| ACTG2 | 72 | actin, gamma 2, smooth muscle, enteric (ACTG2), mRNA. | 2.65 | 2.55 | 0.0007 |
| TCN2 | 6948 | transcobalamin II; macrocytic anemia (TCN2), mRNA. | 2.65 | 9.07 | 0 |
| KRT6A | 3853 | keratin 6A (KRT6A), mRNA. | 2.65 | 5.44 | 0 |
| ACAP1 | 9744 | ArfGAP with coiled-coil, ankyrin repeat and PH domains 1 (ACAP1), mRNA. | 2.64 | 8.02 | 0 |
| RUNX3 | 864 | runt-related transcription factor 3 (RUNX3), transcript variant 2, mRNA. | 2.63 | 7.80 | 0 |
| ZNF683 | 257101 | zinc finger protein 683 (ZNF683), transcript variant 2, mRNA. | 2.63 | 5.22 | 0 |
| NUB1 | 51667 | negative regulator of ubiquitin-like proteins 1 (NUB1), mRNA. | 2.63 | 9.52 | 0 |
| PRR4 | 11272 | proline rich 4 (lacrimal) (PRR4), transcript variant 1, mRNA. | 2.62 | 2.27 | 0.002 |
| BTN3A2 | 11118 | butyrophilin, subfamily 3, member A2 (BTN3A2), mRNA. | 2.60 | 6.38 | 0 |
| HS.489254 |  | cDNA clone IMAGE:5277162 | 2.60 | 8.45 | 0 |
| GRN | 2896 | granulin (GRN), mRNA. | 2.60 | 4.81 | 0 |
| PLSCR1 | 5359 | phospholipid scramblase 1 (PLSCR1), mRNA. | 2.59 | 5.69 | 0 |
| SP140 | 11262 | SP140 nuclear body protein (SP140), transcript variant 1, mRNA./// SP140 nuclear body protein (SP140), transcript variant 2, mRNA. | 2.59 | 7.01 | 0 |
| SERPING1 | 710 | serpin peptidase inhibitor, clade G (C1 inhibitor), member 1 (SERPING1), transcript variant 2, mRNA./// serpin peptidase inhibitor, clade G (C1 inhibitor), member 1 (SERPING1), transcript variant 1, mRNA. | 2.59 | 6.40 | 0 |
| PIK3AP1 | 118788 | phosphoinositide-3-kinase adaptor protein 1 (PIK3AP1), mRNA. | 2.58 | 7.50 | 0 |
| TRAF3IP3 | 80342 | TRAF3 interacting protein 3 (TRAF3IP3), mRNA. | 2.57 | 6.29 | 0 |
| OTOF | 9381 | otoferlin (OTOF), transcript variant 4, mRNA. | 2.57 | 5.23 | 0 |
| DEFB103A | 55894 | defensin, beta 103A (DEFB103A), mRNA. | 2.57 | 3.97 | 0 |
| INPP5D | 3635 | inositol polyphosphate-5-phosphatase, 145kDa (INPP5D), transcript variant 2, mRNA. | 2.57 | 8.07 | 0 |
| C20ORF103 | 24141 | chromosome 20 open reading frame 103 (C20orf103), mRNA. | 2.57 | 5.27 | 0 |
| RNASE7 | 84659 | ribonuclease, RNase A family, 7 (RNASE7), mRNA. | 2.56 | 3.69 | 0 |
| MFNG | 4242 | MFNG O-fucosylpeptide 3-beta-N-acetylglucosaminyltransferase (MFNG), mRNA. | 2.56 | 8.13 | 0 |
| GMFG | 9535 | glia maturation factor, gamma (GMFG), mRNA. | 2.56 | 5.91 | 0 |
| MICAL1 | 64780 | microtubule associated monoxygenase, calponin and LIM domain containing 1 (MICAL1), mRNA. | 2.55 | 9.30 | 0 |
| C2 | 717 | complement component 2 (C2), mRNA. | 2.55 | 9.64 | 0 |
| DOCK8 | 81704 | dedicator of cytokinesis 8 (DOCK8), mRNA. | 2.54 | 6.50 | 0 |
| LYNX1 | 66004 | Ly6/neurotoxin 1 (LYNX1), transcript variant SLURP2, mRNA. | 2.54 | 3.74 | 0 |
| FYB | 2533 | FYN binding protein (FYB-120/130) (FYB), transcript variant 1, mRNA. | 2.54 | 5.39 | 0 |
| IKZF1 | 10320 | IKAROS family zinc finger 1 (Ikaros) (IKZF1), mRNA. | 2.54 | 6.95 | 0 |
| CD14 | 929 | CD14 molecule (CD14), transcript variant 1, mRNA./// CD14 molecule (CD14), transcript variant 2, mRNA. | 2.53 | 5.44 | 0 |
| TMEM119 | 338773 | transmembrane protein 119 (TMEM119), mRNA. | 2.52 | 5.16 | 0 |
| CARD11 | 84433 | caspase recruitment domain family, member 11 (CARD11), mRNA. | 2.52 | 7.65 | 0 |
| SPATS2L | 26010 | spermatogenesis associated, serine-rich 2-like (SPATS2L), transcript variant 2, mRNA. | 2.51 | 8.47 | 0 |
| CCL3L3 | 414062 | chemokine (C-C motif) ligand 3-like 3 (CCL3L3), mRNA. | 2.50 | 6.15 | 0 |
| C5ORF20 | 140947 | chromosome 5 open reading frame 20 (C5orf20), mRNA. | 2.50 | 6.33 | 0 |
| ST6GAL1 | 6480 | ST6 beta-galactosamide alpha-2,6-sialyltranferase 1 (ST6GAL1), transcript variant 2, mRNA./// ST6 beta-galactosamide alpha-2,6-sialyltranferase 1 (ST6GAL1), transcript variant 1, mRNA. | 2.50 | 5.89 | 0 |
| ICAM2 | 3384 | intercellular adhesion molecule 2 (ICAM2), transcript variant 1, mRNA. | 2.50 | 6.72 | 0 |
| AMICA1 | 120425 | adhesion molecule, interacts with CXADR antigen 1 (AMICA1), mRNA. | 2.49 | 4.84 | 0 |
| PSCDBP///CYTIP | 9595 | pleckstrin homology, Sec7 and coiled-coil domains, binding protein (PSCDBP), mRNA./// cytohesin 1 interacting protein (CYTIP), mRNA. | 2.48 | 5.74 | 0 |
| RBCK1 | 10616 | RanBP-type and C3HC4-type zinc finger containing 1 (RBCK1), transcript variant 1, mRNA./// RanBP-type and C3HC4-type zinc finger containing 1 (RBCK1), transcript variant 2, mRNA. | 2.48 | 8.05 | 0 |
| SLAMF8 | 56833 | SLAM family member 8 (SLAMF8), mRNA. | 2.48 | 7.47 | 0 |
| CSF1R | 1436 | colony stimulating factor 1 receptor, formerly McDonough feline sarcoma viral (v-fms) oncogene homolog (CSF1R), mRNA. | 2.48 | 4.94 | 0 |
| P2RY6 | 5031 | pyrimidinergic receptor P2Y, G-protein coupled, 6 (P2RY6), transcript variant 3, mRNA. | 2.48 | 7.21 | 0 |
| GLIPR2 | 152007 | GLI pathogenesis-related 2 (GLIPR2), mRNA. | 2.47 | 10.05 | 0 |
| IL18RAP | 8807 | interleukin 18 receptor accessory protein (IL18RAP), mRNA. | 2.45 | 4.71 | 0 |
| CNFN | 84518 | cornifelin (CNFN), mRNA. | 2.45 | 3.60 | 0 |
| CYFIP2 | 26999 | cytoplasmic FMR1 interacting protein 2 (CYFIP2), transcript variant 1, mRNA./// cytoplasmic FMR1 interacting protein 2 (CYFIP2), transcript variant 3, mRNA. | 2.45 | 7.70 | 0 |
| SERPINB13 | 5275 | serpin peptidase inhibitor, clade B (ovalbumin), member 13 (SERPINB13), mRNA. | 2.45 | 4.28 | 0 |
| VAMP5 | 10791 | vesicle-associated membrane protein 5 (myobrevin) (VAMP5), mRNA. | 2.45 | 9.33 | 0 |
| ADA | 100 | adenosine deaminase (ADA), mRNA. | 2.45 | 7.77 | 0 |
| GAPT | 202309 | GRB2-binding adaptor protein, transmembrane (GAPT), mRNA. | 2.44 | 4.81 | 0 |
| IFIT5 | 24138 | interferon-induced protein with tetratricopeptide repeats 5 (IFIT5), mRNA. | 2.44 | 10.66 | 0 |
| TAPBP | 6892 | TAP binding protein (tapasin) (TAPBP), transcript variant 3, mRNA./// TAP binding protein (tapasin) (TAPBP), transcript variant 2, mRNA./// TAP binding protein (tapasin) (TAPBP), transcript variant 1, mRNA. | 2.44 | 7.72 | 0 |
| EMP3 | 2014 | epithelial membrane protein 3 (EMP3), mRNA. | 2.44 | 6.62 | 0 |
| TCN1 | 6947 | transcobalamin I (vitamin B12 binding protein, R binder family) (TCN1), mRNA. | 2.44 | 2.31 | 0.002 |
| FCN1 | 2219 | ficolin (collagen/fibrinogen domain containing) 1 (FCN1), mRNA. | 2.43 | 4.19 | 0 |
| LCE6A | 448835 | late cornified envelope 6A (LCE6A), mRNA. | 2.43 | 2.96 | 0 |
| ITM2C | 81618 | integral membrane protein 2C (ITM2C), transcript variant 2, mRNA. | 2.43 | 4.24 | 0 |
| CCL19 | 6363 | chemokine (C-C motif) ligand 19 (CCL19), mRNA. | 2.42 | 4.27 | 0 |
| CD68 | 968 | CD68 molecule (CD68), transcript variant 1, mRNA./// CD68 antigen (CD68), mRNA. | 2.42 | 10.82 | 0 |
| PTGER2 | 5732 | prostaglandin E receptor 2 (subtype EP2), 53kDa (PTGER2), mRNA. | 2.42 | 7.42 | 0 |
| TCIRG1 | 10312 | T-cell, immune regulator 1, ATPase, H+ transporting, lysosomal V0 subunit A3 (TCIRG1), transcript variant 1, mRNA./// T-cell, immune regulator 1, ATPase, H+ transporting, lysosomal V0 subunit A3 (TCIRG1), transcript variant 2, mRNA. | 2.42 | 9.08 | 0 |
| NCF1C | 654817 | neutrophil cytosolic factor 1C pseudogene (NCF1C), non-coding RNA. | 2.41 | 7.02 | 0 |
| PHF15 | 23338 | PHD finger protein 15 (PHF15), mRNA. | 2.41 | 7.41 | 0 |
| EVL | 51466 | Enah/Vasp-like (EVL), mRNA. | 2.40 | 6.55 | 0 |
| CD79B | 974 | CD79b molecule, immunoglobulin-associated beta (CD79B), transcript variant 3, mRNA./// CD79B antigen (immunoglobulin-associated beta) (CD79B), transcript variant 2, mRNA./// CD79B antigen (immunoglobulin-associated beta) (CD79B), transcript variant 1, mRNA. | 2.39 | 4.78 | 0 |
| ARHGEF3 | 50650 | Rho guanine nucleotide exchange factor (GEF) 3 (ARHGEF3), mRNA. | 2.39 | 6.95 | 0 |
| S100A9 | 6280 | S100 calcium binding protein A9 (calgranulin B) (S100A9), mRNA. | 2.38 | 2.19 | 0.003 |
| DRAM1 | 55332 | DNA-damage regulated autophagy modulator 1 (DRAM1), mRNA. | 2.38 | 9.32 | 0 |
| FXYD5 | 53827 | FXYD domain containing ion transport regulator 5 (FXYD5), transcript variant 2, mRNA./// FXYD domain containing ion transport regulator 5 (FXYD5), transcript variant 1, mRNA. | 2.37 | 6.19 | 0 |
| HLA-DQA1 | 3117 | PREDICTED: major histocompatibility complex, class II, DQ alpha 1, transcript variant 10 (HLA-DQA1), mRNA. | 2.37 | 4.31 | 0 |
| RASGRP3 | 25780 | RAS guanyl releasing protein 3 (calcium and DAG-regulated) (RASGRP3), mRNA. | 2.37 | 7.32 | 0 |
| RGL1 | 23179 | ral guanine nucleotide dissociation stimulator-like 1 (RGL1), mRNA. | 2.36 | 6.82 | 0 |
| CST3 | 1471 | cystatin C (CST3), mRNA. | 2.36 | 6.38 | 0 |
| LOC100133678 | 100133678 | PREDICTED: similar to hCG2042724 (LOC100133678), partial mRNA. | 2.36 | 4.53 | 0 |
| PLA1A | 51365 | phospholipase A1 member A (PLA1A), mRNA. | 2.36 | 7.22 | 0 |
| S100A7 | 6278 | S100 calcium binding protein A7 (S100A7), mRNA. | 2.36 | 2.12 | 0.003 |
| NT5C3 | 51251 | 5'-nucleotidase, cytosolic III (NT5C3), transcript variant 1, mRNA. | 2.35 | 6.30 | 0 |
| LOC728835 | 728835 | PREDICTED: similar to cytokine, transcript variant 3 (LOC728835), mRNA. | 2.35 | 6.92 | 0 |
| PPP1R16B | 26051 | protein phosphatase 1, regulatory (inhibitor) subunit 16B (PPP1R16B), mRNA. | 2.34 | 6.61 | 0 |
| LOC401115///C4ORF48 | 401115 | PREDICTED: hypothetical gene supported by BC038466; BC062790 (LOC401115), mRNA./// chromosome 4 open reading frame 48 (C4orf48), mRNA. | 2.34 | 9.92 | 0 |
| SPRR2D | 6703 | small proline-rich protein 2D (SPRR2D), mRNA. | 2.34 | 2.87 | 0.0007 |
| TXNDC3 | 51314 | thioredoxin domain containing 3 (spermatozoa) (TXNDC3), mRNA. | 2.34 | 4.79 | 0 |
| NLRC5 | 84166 | NLR family, CARD domain containing 5 (NLRC5), mRNA. | 2.34 | 10.46 | 0 |
| LOC100130229 | 100130229 | PREDICTED: hypothetical protein LOC100130229 (LOC100130229), mRNA. | 2.34 | 5.25 | 0 |
| ARHGDIB | 397 | Rho GDP dissociation inhibitor (GDI) beta (ARHGDIB), mRNA. | 2.34 | 7.06 | 0 |
| MMP7 | 4316 | matrix metallopeptidase 7 (matrilysin, uterine) (MMP7), mRNA. | 2.33 | 2.22 | 0.002 |
| LY96 | 23643 | lymphocyte antigen 96 (LY96), mRNA. | 2.33 | 4.74 | 0 |
| CD97 | 976 | CD97 molecule (CD97), transcript variant 1, mRNA./// CD97 molecule (CD97), transcript variant 2, mRNA. | 2.32 | 7.45 | 0 |
| FABP5 | 2171 | fatty acid binding protein 5 (psoriasis-associated) (FABP5), mRNA. | 2.32 | 4.10 | 0 |
| BASP1 | 10409 | brain abundant, membrane attached signal protein 1 (BASP1), mRNA. | 2.31 | 4.98 | 0 |
| CYP2J2 | 1573 | cytochrome P450, family 2, subfamily J, polypeptide 2 (CYP2J2), mRNA. | 2.31 | 4.37 | 0 |
| ARHGAP9 | 64333 | Rho GTPase activating protein 9 (ARHGAP9), transcript variant 2, mRNA./// Rho GTPase activating protein 9 (ARHGAP9), transcript variant 3, mRNA. | 2.31 | 5.61 | 0 |
| GBP6 | 163351 | guanylate binding protein family, member 6 (GBP6), mRNA. | 2.31 | 7.94 | 0 |
| DTX3L | 151636 | deltex 3-like (Drosophila) (DTX3L), mRNA. | 2.30 | 8.64 | 0 |
| HS.534439 |  | full-length cDNA clone CS0CAP005YH21 of Thymus of (human) | 2.30 | 4.89 | 0 |
| IL32 | 9235 | interleukin 32 (IL32), transcript variant 4, mRNA./// interleukin 32 (IL32), transcript variant 7, mRNA. | 2.30 | 5.04 | 0 |
| C1ORF54 | 79630 | chromosome 1 open reading frame 54 (C1orf54), mRNA. | 2.29 | 7.14 | 0 |
| FAM65B | 9750 | family with sequence similarity 65, member B (FAM65B), transcript variant 2, mRNA. | 2.29 | 5.47 | 0 |
| CAMK1G | 57172 | calcium/calmodulin-dependent protein kinase IG (CAMK1G), mRNA. | 2.28 | 3.20 | 0 |
| THY1 | 7070 | Thy-1 cell surface antigen (THY1), mRNA. | 2.28 | 6.53 | 0 |
| TUBB3 | 10381 | tubulin, beta 3 (TUBB3), mRNA. | 2.28 | 3.19 | 0 |
| ENPP2 | 5168 | ectonucleotide pyrophosphatase/phosphodiesterase 2 (ENPP2), transcript variant 2, mRNA. | 2.27 | 6.03 | 0 |
| CSK | 1445 | c-src tyrosine kinase (CSK), mRNA. | 2.27 | 7.07 | 0 |
| LOC642956 | 642956 | PREDICTED: hypothetical LOC642956 (LOC642956), mRNA. | 2.27 | 3.69 | 0 |
| HLA-DOB | 3112 | major histocompatibility complex, class II, DO beta (HLA-DOB), mRNA. | 2.26 | 5.84 | 0 |
| RGS19 | 10287 | regulator of G-protein signaling 19 (RGS19), transcript variant 1, mRNA. | 2.26 | 6.90 | 0 |
| APBB1IP | 54518 | amyloid beta (A4) precursor protein-binding, family B, member 1 interacting protein (APBB1IP), mRNA. | 2.26 | 6.93 | 0 |
| AKR1B15 | 441282 | aldo-keto reductase family 1, member B15 (AKR1B15), mRNA. | 2.26 | 5.05 | 0 |
| SUSD3 | 203328 | sushi domain containing 3 (SUSD3), mRNA. | 2.26 | 7.28 | 0 |
| CCND3 | 896 | cyclin D3 (CCND3), mRNA. | 2.25 | 7.06 | 0 |
| TRIM38 | 10475 | tripartite motif-containing 38 (TRIM38), mRNA. | 2.25 | 9.88 | 0 |
| NCF1 | 653361 | neutrophil cytosolic factor 1 (NCF1), mRNA. | 2.25 | 5.30 | 0 |
| TRIM5 | 85363 | tripartite motif-containing 5 (TRIM5), transcript variant gamma, mRNA./// tripartite motif-containing 5 (TRIM5), transcript variant alpha, mRNA./// tripartite motif-containing 5 (TRIM5), transcript variant delta, mRNA. | 2.25 | 9.27 | 0 |
| TRPV2 | 51393 | transient receptor potential cation channel, subfamily V, member 2 (TRPV2), mRNA. | 2.24 | 7.67 | 0 |
| HOXB2 | 3212 | homeobox B2 (HOXB2), mRNA. | 2.24 | 8.68 | 0 |
| SIAE | 54414 | sialic acid acetylesterase (SIAE), mRNA. | 2.24 | 2.94 | 0 |
| SKAP1 | 8631 | src kinase associated phosphoprotein 1 (SKAP1), transcript variant 1, mRNA./// src kinase associated phosphoprotein 1 (SKAP1), transcript variant 2, mRNA. | 2.24 | 6.20 | 0 |
| LOC729086 | 729086 | PREDICTED: similar to EGFR-coamplified and overexpressed protein (LOC729086), mRNA. | 2.24 | 8.79 | 0 |
| SH2B3 | 10019 | SH2B adaptor protein 3 (SH2B3), mRNA. | 2.24 | 6.75 | 0 |
| TLR10 | 81793 | toll-like receptor 10 (TLR10), transcript variant 1, mRNA./// toll-like receptor 10 (TLR10), transcript variant 2, mRNA. | 2.23 | 4.45 | 0 |
| APOBEC3D | 140564 | apolipoprotein B mRNA editing enzyme, catalytic polypeptide-like 3D (APOBEC3D), mRNA. | 2.23 | 8.81 | 0 |
| GBP2 | 2634 | guanylate binding protein 2, interferon-inducible (GBP2), mRNA. | 2.23 | 9.90 | 0 |
| SMAP2 | 64744 | small ArfGAP2 (SMAP2), mRNA. | 2.22 | 5.97 | 0 |
| LOC100130246 | 100130246 | PREDICTED: hypothetical protein LOC100130246 (LOC100130246), mRNA. | 2.22 | 1.87 | 0.005 |
| CST7 | 8530 | cystatin F (leukocystatin) (CST7), mRNA. | 2.22 | 6.35 | 0 |
| VWF | 7450 | von Willebrand factor (VWF), mRNA. | 2.21 | 3.42 | 0 |
| CALML5 | 51806 | calmodulin-like 5 (CALML5), mRNA. | 2.21 | 4.47 | 0 |
| LOC100133583 | 100133583 | PREDICTED: similar to major histocompatibility complex, class II, DQ beta 1, transcript variant 2 (LOC100133583), mRNA. | 2.21 | 3.63 | 0 |
| LIME1 | 54923 | Lck interacting transmembrane adaptor 1 (LIME1), mRNA. | 2.20 | 5.89 | 0 |
| PIK3CD | 5293 | phosphoinositide-3-kinase, catalytic, delta polypeptide (PIK3CD), mRNA. | 2.20 | 7.41 | 0 |
| TNFRSF14 | 8764 | tumor necrosis factor receptor superfamily, member 14 (herpesvirus entry mediator) (TNFRSF14), mRNA. | 2.19 | 5.87 | 0 |
| ADAP2 | 55803 | ArfGAP with dual PH domains 2 (ADAP2), mRNA. | 2.19 | 5.39 | 0 |
| CXCR3 | 2833 | chemokine (C-X-C motif) receptor 3 (CXCR3), transcript variant A, mRNA. | 2.19 | 6.57 | 0 |
| CD248 | 57124 | CD248 molecule, endosialin (CD248), mRNA. | 2.19 | 3.57 | 0 |
| WASPIP///WIPF1 | 7456 | Wiskott-Aldrich syndrome protein interacting protein (WASPIP), mRNA./// WAS/WASL interacting protein family, member 1 (WIPF1), transcript variant 2, mRNA. | 2.18 | 5.73 | 0 |
| STAMBPL1 | 57559 | STAM binding protein-like 1 (STAMBPL1), mRNA. | 2.18 | 5.76 | 0 |
| HS.62927 |  | RST29852 Athersys RAGE Library cDNA, mRNA sequence | 2.18 | 2.65 | 0.0007 |
| AIF1 | 199 | allograft inflammatory factor 1 (AIF1), transcript variant 3, mRNA./// allograft inflammatory factor 1 (AIF1), transcript variant 1, mRNA. | 2.18 | 5.17 | 0 |
| CLIC3 | 9022 | chloride intracellular channel 3 (CLIC3), mRNA. | 2.18 | 3.87 | 0 |
| LOC401845 | 401845 | PREDICTED: similar to Ig heavy chain V-II region SESS precursor (LOC401845), mRNA. | 2.17 | 2.59 | 0.0007 |
| CD4 | 920 | CD4 molecule (CD4), mRNA. | 2.17 | 5.98 | 0 |
| SPRR1A | 6698 | small proline-rich protein 1A (SPRR1A), mRNA. | 2.17 | 3.32 | 0 |
| PYHIN1 | 149628 | pyrin and HIN domain family, member 1 (PYHIN1), transcript variant b2, mRNA./// pyrin and HIN domain family, member 1 (PYHIN1), transcript variant a1, mRNA. | 2.17 | 7.66 | 0 |
| SPI1 | 6688 | spleen focus forming virus (SFFV) proviral integration oncogene spi1 (SPI1), transcript variant 1, mRNA./// spleen focus forming virus (SFFV) proviral integration oncogene spi1 (SPI1), transcript variant 2, mRNA. | 2.17 | 5.41 | 0 |
| ENG | 2022 | endoglin (Osler-Rendu-Weber syndrome 1) (ENG), mRNA. | 2.17 | 4.18 | 0 |
| FKBP11 | 51303 | FK506 binding protein 11, 19 kDa (FKBP11), mRNA. | 2.16 | 6.20 | 0 |
| ADCY7 | 113 | adenylate cyclase 7 (ADCY7), mRNA. | 2.16 | 6.31 | 0 |
| FCGR1B | 2210 | Fc fragment of IgG, high affinity Ib, receptor (CD64) (FCGR1B), transcript variant 2, mRNA./// Fc fragment of IgG, high affinity Ib, receptor (CD64) (FCGR1B), transcript variant 1, mRNA. | 2.16 | 6.99 | 0 |
| FLJ22662 | 79887 | hypothetical protein FLJ22662 (FLJ22662), mRNA. | 2.16 | 5.93 | 0 |
| TSPAN32 | 10077 | tetraspanin 32 (TSPAN32), transcript variant 2, mRNA. | 2.16 | 4.95 | 0 |
| CTSC | 1075 | cathepsin C (CTSC), transcript variant 1, mRNA./// cathepsin C (CTSC), transcript variant 2, mRNA. | 2.16 | 9.95 | 0 |
| GALM | 130589 | galactose mutarotase (aldose 1-epimerase) (GALM), mRNA. | 2.16 | 8.74 | 0 |
| FAM46C | 54855 | family with sequence similarity 46, member C (FAM46C), mRNA. | 2.15 | 2.77 | 0.0007 |
| FAM46A | 55603 | family with sequence similarity 46, member A (FAM46A), mRNA. | 2.15 | 4.98 | 0 |
| APOL2 | 23780 | apolipoprotein L, 2 (APOL2), transcript variant beta, mRNA. | 2.15 | 7.56 | 0 |
| C1S | 716 | complement component 1, s subcomponent (C1S), transcript variant 1, mRNA./// complement component 1, s subcomponent (C1S), transcript variant 2, mRNA. | 2.15 | 6.52 | 0 |
| SLFN5 | 162394 | schlafen family member 5 (SLFN5), mRNA. | 2.14 | 5.84 | 0 |
| CRKRS | 51755 | Cdc2-related kinase, arginine/serine-rich (CRKRS), mRNA. | 2.14 | 3.01 | 0 |
| RNASE1 | 6035 | ribonuclease, RNase A family, 1 (pancreatic) (RNASE1), transcript variant 3, mRNA./// ribonuclease, RNase A family, 1 (pancreatic) (RNASE1), transcript variant 1, mRNA. | 2.14 | 3.50 | 0 |
| C17ORF62 | 79415 | chromosome 17 open reading frame 62 (C17orf62), transcript variant 3, mRNA. | 2.14 | 8.04 | 0 |
| CCM2 | 83605 | cerebral cavernous malformation 2 (CCM2), transcript variant 2, mRNA./// cerebral cavernous malformation 2 (CCM2), transcript variant 1, mRNA. | 2.14 | 7.71 | 0 |
| LCE2C | 353140 | late cornified envelope 2C (LCE2C), mRNA. | 2.14 | 3.26 | 0 |
| LCE1B | 353132 | late cornified envelope 1B (LCE1B), mRNA. | 2.13 | 2.52 | 0.001 |
| PSME1 | 5720 | proteasome (prosome, macropain) activator subunit 1 (PA28 alpha) (PSME1), transcript variant 1, mRNA./// proteasome (prosome, macropain) activator subunit 1 (PA28 alpha) (PSME1), transcript variant 2, mRNA. | 2.13 | 7.55 | 0 |
| ARHGAP30 | 257106 | Rho GTPase activating protein 30 (ARHGAP30), transcript variant 1, mRNA./// Rho GTPase activating protein 30 (ARHGAP30), transcript variant 2, mRNA. | 2.13 | 7.28 | 0 |
| LTA | 4049 | lymphotoxin alpha (TNF superfamily, member 1) (LTA), transcript variant 2, mRNA. | 2.13 | 6.78 | 0 |
| LCE1A | 353131 | late cornified envelope 1A (LCE1A), mRNA. | 2.13 | 2.39 | 0.001 |
| GZMH | 2999 | granzyme H (cathepsin G-like 2, protein h-CCPX) (GZMH), mRNA. | 2.13 | 6.83 | 0 |
| OGFR | 11054 | opioid growth factor receptor (OGFR), mRNA. | 2.12 | 8.86 | 0 |
| IFI16 | 3428 | interferon, gamma-inducible protein 16 (IFI16), mRNA. | 2.12 | 5.01 | 0 |
| CCL4L1 | 9560 | chemokine (C-C motif) ligand 4-like 1 (CCL4L1), mRNA. | 2.12 | 5.73 | 0 |
| MYD88 | 4615 | myeloid differentiation primary response gene (88) (MYD88), mRNA. | 2.12 | 6.22 | 0 |
| TMSB10 | 9168 | thymosin beta 10 (TMSB10), mRNA. | 2.12 | 11.62 | 0 |
| HLA-DRA | 3122 | major histocompatibility complex, class II, DR alpha (HLA-DRA), mRNA. | 2.12 | 8.65 | 0 |
| RHOG | 391 | ras homolog gene family, member G (rho G) (RHOG), mRNA. | 2.12 | 5.58 | 0 |
| FCGR1A | 2209 | Fc fragment of IgG, high affinity Ia, receptor (CD64) (FCGR1A), mRNA. | 2.12 | 6.87 | 0 |
| MLKL | 197259 | PREDICTED: mixed lineage kinase domain-like (MLKL), mRNA./// mixed lineage kinase domain-like (MLKL), mRNA. | 2.11 | 8.63 | 0 |
| PLCB2 | 5330 | phospholipase C, beta 2 (PLCB2), mRNA. | 2.11 | 7.09 | 0 |
| LOC392437 | 392437 | PREDICTED: misc_RNA (LOC392437), miscRNA. | 2.10 | 5.52 | 0 |
| CSTA | 1475 | cystatin A (stefin A) (CSTA), mRNA. | 2.10 | 3.80 | 0 |
| HRNR | 388697 | hornerin (HRNR), mRNA. | 2.10 | 2.20 | 0.003 |
| LOC652775 | 652775 | PREDICTED: similar to Ig kappa chain V-V region L7 precursor (LOC652775), mRNA. | 2.09 | 2.86 | 0.0007 |
| CLECL1 | 160365 | C-type lectin-like 1 (CLECL1), mRNA. | 2.09 | 4.23 | 0 |
| NCF4 | 4689 | neutrophil cytosolic factor 4, 40kDa (NCF4), transcript variant 1, mRNA./// neutrophil cytosolic factor 4, 40kDa (NCF4), transcript variant 2, mRNA. | 2.09 | 6.35 | 0 |
| EMILIN2 | 84034 | elastin microfibril interfacer 2 (EMILIN2), mRNA. | 2.09 | 4.71 | 0 |
| CCR1 | 1230 | chemokine (C-C motif) receptor 1 (CCR1), mRNA. | 2.09 | 4.76 | 0 |
| LCE2B | 26239 | late cornified envelope 2B (LCE2B), mRNA. | 2.09 | 2.91 | 0.0007 |
| MS4A7 | 58475 | membrane-spanning 4-domains, subfamily A, member 7 (MS4A7), transcript variant 2, mRNA. | 2.08 | 4.46 | 0 |
| HCK | 3055 | hemopoietic cell kinase (HCK), mRNA. | 2.08 | 5.25 | 0 |
| ZFYVE26 | 23503 | zinc finger, FYVE domain containing 26 (ZFYVE26), mRNA. | 2.08 | 6.62 | 0 |
| C17ORF60 | 284021 | chromosome 17 open reading frame 60 (C17orf60), mRNA. | 2.07 | 7.16 | 0 |
| SYK | 6850 | spleen tyrosine kinase (SYK), mRNA. | 2.07 | 6.48 | 0 |
| LOC643161///FAM25A | 643161 | PREDICTED: hypothetical LOC643161 (LOC643161), mRNA.///PREDICTED: family with sequence similarity 25, member A (FAM25A), mRNA. | 2.06 | 2.44 | 0.001 |
| TNFRSF1B | 7133 | tumor necrosis factor receptor superfamily, member 1B (TNFRSF1B), mRNA. | 2.06 | 4.28 | 0 |
| MARCH1 | 55016 | membrane-associated ring finger (C3HC4) 1 (MARCH1), mRNA. | 2.06 | 5.63 | 0 |
| CTLA4 | 1493 | cytotoxic T-lymphocyte-associated protein 4 (CTLA4), transcript variant 1, mRNA. | 2.06 | 4.61 | 0 |
| TRIM25 | 7706 | tripartite motif-containing 25 (TRIM25), mRNA. | 2.06 | 6.81 | 0 |
| LCE2D | 353141 | late cornified envelope 2D (LCE2D), mRNA. | 2.06 | 2.75 | 0.0007 |
| SOCS1 | 8651 | suppressor of cytokine signaling 1 (SOCS1), mRNA. | 2.06 | 5.96 | 0 |
| CNTNAP2 | 26047 | contactin associated protein-like 2 (CNTNAP2), mRNA. | 2.05 | 5.98 | 0 |
| LOC647993 | 647993 | PREDICTED: hypothetical protein LOC647993 (LOC647993), mRNA. | 2.05 | 2.48 | 0.001 |
| HIST2H2AA4 | 723790 | histone cluster 2, H2aa4 (HIST2H2AA4), mRNA. | 2.05 | 6.03 | 0 |
| MIR155HG | 114614 | MIR155 host gene (non-protein coding) (MIR155HG), non-coding RNA. | 2.05 | 5.39 | 0 |
| WDFY1 | 57590 | WD repeat and FYVE domain containing 1 (WDFY1), mRNA. | 2.04 | 8.10 | 0 |
| RAB8A | 4218 | RAB8A, member RAS oncogene family (RAB8A), mRNA. | 2.04 | 7.79 | 0 |
| LOC729009 | 729009 | PREDICTED: misc_RNA (LOC729009), miscRNA. | 2.04 | 5.26 | 0 |
| HLA-DOA | 3111 | major histocompatibility complex, class II, DO alpha (HLA-DOA), mRNA. | 2.04 | 5.32 | 0 |
| PRKCQ | 5588 | protein kinase C, theta (PRKCQ), mRNA. | 2.04 | 7.64 | 0 |
| HAPLN3 | 145864 | hyaluronan and proteoglycan link protein 3 (HAPLN3), mRNA. | 2.04 | 7.18 | 0 |
| KLRG1 | 10219 | killer cell lectin-like receptor subfamily G, member 1 (KLRG1), mRNA. | 2.04 | 5.06 | 0 |
| LOC643479 | 643479 | PREDICTED: hypothetical LOC643479 (LOC643479), mRNA. | 2.04 | 2.73 | 0.0007 |
| SULF1 | 23213 | sulfatase 1 (SULF1), mRNA. | 2.04 | 4.17 | 0 |
| FYN | 2534 | FYN oncogene related to SRC, FGR, YES (FYN), transcript variant 2, mRNA./// FYN oncogene related to SRC, FGR, YES (FYN), transcript variant 1, mRNA./// FYN oncogene related to SRC, FGR, YES (FYN), transcript variant 3, mRNA. | 2.03 | 7.49 | 0 |
| CXCR4 | 7852 | chemokine (C-X-C motif) receptor 4 (CXCR4), transcript variant 1, mRNA./// chemokine (C-X-C motif) receptor 4 (CXCR4), transcript variant 2, mRNA. | 2.03 | 3.61 | 0 |
| MVP | 9961 | major vault protein (MVP), transcript variant 1, mRNA./// major vault protein (MVP), transcript variant 2, mRNA. | 2.03 | 7.20 | 0 |
| HIST2H2AC | 8338 | histone cluster 2, H2ac (HIST2H2AC), mRNA. | 2.03 | 6.16 | 0 |
| GNAI2 | 2771 | guanine nucleotide binding protein (G protein), alpha inhibiting activity polypeptide 2 (GNAI2), transcript variant 1, mRNA. | 2.03 | 4.95 | 0 |
| FSCN1 | 6624 | fascin homolog 1, actin-bundling protein (Strongylocentrotus purpuratus) (FSCN1), mRNA. | 2.03 | 3.04 | 0 |
| GMIP | 51291 | GEM interacting protein (GMIP), mRNA. | 2.03 | 6.96 | 0 |
| MAP4K2 | 5871 | mitogen-activated protein kinase kinase kinase kinase 2 (MAP4K2), mRNA. | 2.03 | 6.79 | 0 |
| RGS18 | 64407 | regulator of G-protein signaling 18 (RGS18), mRNA. | 2.02 | 3.69 | 0 |
| LOC339192 | 339192 | PREDICTED: hypothetical protein LOC339192 (LOC339192), mRNA. | 2.02 | 7.68 | 0 |
| S100A8 | 6279 | S100 calcium binding protein A8 (S100A8), mRNA. | 2.02 | 1.83 | 0.005 |
| HLA-DRB5 | 3127 | major histocompatibility complex, class II, DR beta 5 (HLA-DRB5), mRNA. | 2.01 | 1.02 | 0.01 |
| CDSN | 1041 | corneodesmosin (CDSN), mRNA. | 2.01 | 2.62 | 0.0007 |
| RIPK3 | 11035 | receptor-interacting serine-threonine kinase 3 (RIPK3), mRNA. | 2.01 | 8.05 | 0 |
| CTSK | 1513 | cathepsin K (CTSK), mRNA. | 2.01 | 6.36 | 0 |
| KLRB1 | 3820 | killer cell lectin-like receptor subfamily B, member 1 (KLRB1), mRNA. | 2.00 | 4.24 | 0 |
| KPRP | 448834 | keratinocyte proline-rich protein (KPRP), mRNA. | 2.00 | 2.19 | 0.003 |
| DNMT1 | 1786 | DNA (cytosine-5-)-methyltransferase 1 (DNMT1), mRNA. | 2.00 | 4.59 | 0 |
| LOC728855 | 728855 | hypothetical LOC728855 (LOC728855), non-coding RNA. | 2.00 | 6.72 | 0 |
| LMO2 | 4005 | LIM domain only 2 (rhombotin-like 1) (LMO2), mRNA. | 2.00 | 6.42 | 0 |
| CARHSP1 | 23589 | calcium regulated heat stable protein 1, 24kDa (CARHSP1), transcript variant 2, mRNA. | 2.00 | 4.32 | 0 |
| LONRF1 | 91694 | LON peptidase N-terminal domain and ring finger 1 (LONRF1), mRNA. | -2.00 | -5.57 | 0 |
| LOC645236 | 645236 | PREDICTED: similar to similar to RPL23AP7 protein (LOC645236), mRNA. | -2.01 | -10.72 | 0 |
| SLC25A6 | 293 | solute carrier family 25 (mitochondrial carrier; adenine nucleotide translocator), member 6 (SLC25A6), nuclear gene encoding mitochondrial protein, mRNA. | -2.01 | -8.51 | 0 |
| RDH11 | 51109 | retinol dehydrogenase 11 (all-trans/9-cis/11-cis) (RDH11), mRNA. | -2.01 | -4.63 | 0 |
| PDHA1 | 5160 | pyruvate dehydrogenase (lipoamide) alpha 1 (PDHA1), mRNA. | -2.01 | -4.56 | 0 |
| CA6 | 765 | carbonic anhydrase VI (CA6), mRNA. | -2.01 | -2.61 | 0.0007 |
| SLC11A2 | 4891 | solute carrier family 11 (proton-coupled divalent metal ion transporters), member 2 (SLC11A2), mRNA. | -2.02 | -5.10 | 0 |
| GPT2 | 84706 | glutamic pyruvate transaminase (alanine aminotransferase) 2 (GPT2), mRNA. | -2.02 | -4.52 | 0 |
| SLC15A1 | 6564 | solute carrier family 15 (oligopeptide transporter), member 1 (SLC15A1), mRNA. | -2.02 | -5.82 | 0 |
| HSPA1A | 3303 | heat shock 70kDa protein 1A (HSPA1A), mRNA. | -2.03 | -2.63 | 0.0007 |
| NACAP1 | 83955 | nascent-polypeptide-associated complex alpha polypeptide pseudogene 1 (NACAP1), non-coding RNA. | -2.03 | -5.58 | 0 |
| ALDH3B2 | 222 | aldehyde dehydrogenase 3 family, member B2 (ALDH3B2), transcript variant 2, mRNA./// aldehyde dehydrogenase 3 family, member B2 (ALDH3B2), transcript variant 1, mRNA. | -2.04 | -3.07 | 0 |
| SDC4 | 6385 | syndecan 4 (SDC4), mRNA. | -2.04 | -4.48 | 0 |
| BRP44L | 51660 | brain protein 44-like (BRP44L), mRNA. | -2.04 | -5.01 | 0 |
| LOC100128771 | 100128771 | PREDICTED: misc_RNA (LOC100128771), miscRNA. | -2.05 | -10.92 | 0 |
| GNG12 | 55970 | guanine nucleotide binding protein (G protein), gamma 12 (GNG12), mRNA. | -2.05 | -3.61 | 0 |
| IQCG | 84223 | IQ motif containing G (IQCG), mRNA. | -2.05 | -5.40 | 0 |
| PCTP | 58488 | phosphatidylcholine transfer protein (PCTP), mRNA. | -2.05 | -3.41 | 0 |
| LDHD | 197257 | lactate dehydrogenase D (LDHD), nuclear gene encoding mitochondrial protein, transcript variant 2, mRNA. | -2.05 | -3.54 | 0 |
| LOC646527 | 646527 | PREDICTED: misc_RNA (LOC646527), miscRNA. | -2.05 | -4.93 | 0 |
| ABHD12B | 145447 | abhydrolase domain containing 12B (ABHD12B), transcript variant 2, mRNA. | -2.06 | -3.54 | 0 |
| ACAD8 | 27034 | acyl-Coenzyme A dehydrogenase family, member 8 (ACAD8), mRNA./// acyl-Coenzyme A dehydrogenase family, member 8 (ACAD8), nuclear gene encoding mitochondrial protein, mRNA. | -2.06 | -4.35 | 0 |
| SOX7 | 83595 | SRY (sex determining region Y)-box 7 (SOX7), mRNA. | -2.07 | -4.95 | 0 |
| ANO1///TMEM16A | 55107 | anoctamin 1, calcium activated chloride channel (ANO1), transcript variant 1, mRNA./// transmembrane protein 16A (TMEM16A), mRNA. | -2.07 | -3.53 | 0 |
| SLC44A3 | 126969 | solute carrier family 44, member 3 (SLC44A3), mRNA. | -2.07 | -4.00 | 0 |
| CCL27 | 10850 | chemokine (C-C motif) ligand 27 (CCL27), mRNA. | -2.07 | -2.49 | 0.001 |
| CPA3 | 1359 | carboxypeptidase A3 (mast cell) (CPA3), mRNA. | -2.07 | -4.06 | 0 |
| PIK3C2G | 5288 | phosphoinositide-3-kinase, class 2, gamma polypeptide (PIK3C2G), mRNA. | -2.07 | -6.19 | 0 |
| HS.560343 |  | mRNA; cDNA DKFZp686M2414 (from clone DKFZp686M2414) | -2.08 | -4.49 | 0 |
| EI24 | 9538 | etoposide induced 2.4 mRNA (EI24), transcript variant 1, mRNA./// etoposide induced 2.4 mRNA (EI24), transcript variant 2, mRNA. | -2.08 | -5.45 | 0 |
| C2ORF7 | 84279 | chromosome 2 open reading frame 7 (C2orf7), mRNA. | -2.08 | -5.69 | 0 |
| LOC641848 | 641848 | PREDICTED: similar to ribosomal protein S3a (LOC641848), mRNA. | -2.08 | -4.70 | 0 |
| ACOT4 | 122970 | acyl-CoA thioesterase 4 (ACOT4), mRNA. | -2.09 | -3.22 | 0 |
| OLAH | 55301 | oleoyl-ACP hydrolase (OLAH), transcript variant 1, mRNA. | -2.09 | -2.86 | 0.0007 |
| LOC646688 | 646688 | PREDICTED: misc_RNA (LOC646688), miscRNA. | -2.09 | -11.03 | 0 |
| HS.335413 |  | , clone IMAGE:5241654, mRNA | -2.09 | -7.12 | 0 |
| CCDC3 | 83643 | coiled-coil domain containing 3 (CCDC3), mRNA. | -2.10 | -3.00 | 0 |
| IL20 | 50604 | interleukin 20 (IL20), mRNA. | -2.10 | -3.87 | 0 |
| ZNF807///LOC643719 | 643719 | PREDICTED: zinc finger protein 807 (ZNF807), mRNA.///PREDICTED: hypothetical LOC643719 (LOC643719), mRNA. | -2.10 | -1.50 | 0.005 |
| PTPLB | 201562 | protein tyrosine phosphatase-like (proline instead of catalytic arginine), member b (PTPLB), mRNA. | -2.10 | -6.46 | 0 |
| C1ORF122 | 127687 | chromosome 1 open reading frame 122 (C1orf122), mRNA. | -2.10 | -3.26 | 0 |
| EGR2 | 1959 | early growth response 2 (Krox-20 homolog, Drosophila) (EGR2), mRNA. | -2.10 | -4.63 | 0 |
| HS.190748 |  | PREDICTED: hypothetical LOC388214 (LOC388214), mRNA | -2.10 | -3.50 | 0 |
| SLC41A1 | 254428 | solute carrier family 41, member 1 (SLC41A1), mRNA. | -2.10 | -5.16 | 0 |
| KIAA0114 | 57291 | KIAA0114 (KIAA0114), non-coding RNA. | -2.11 | -12.58 | 0 |
| APOE | 348 | apolipoprotein E (APOE), mRNA. | -2.11 | -3.86 | 0 |
| TKT | 7086 | transketolase (Wernicke-Korsakoff syndrome) (TKT), mRNA. | -2.11 | -5.44 | 0 |
| C8ORF13///FAM167A | 83648 | chromosome 8 open reading frame 13 (C8orf13), mRNA./// family with sequence similarity 167, member A (FAM167A), mRNA. | -2.11 | -5.66 | 0 |
| LOC730107 | 730107 | PREDICTED: similar to Glycine cleavage system H protein, mitochondrial (LOC730107), mRNA. | -2.11 | -4.70 | 0 |
| CD59 | 966 | CD59 molecule, complement regulatory protein (CD59), transcript variant 2, mRNA./// CD59 molecule, complement regulatory protein (CD59), transcript variant 4, mRNA. | -2.12 | -5.04 | 0 |
| CYR61 | 3491 | cysteine-rich, angiogenic inducer, 61 (CYR61), mRNA. | -2.13 | -1.99 | 0.003 |
| ZNF91 | 7644 | zinc finger protein 91 (ZNF91), mRNA. | -2.13 | -3.99 | 0 |
| TCEAL2 | 140597 | transcription elongation factor A (SII)-like 2 (TCEAL2), mRNA. | -2.13 | -4.00 | 0 |
| IRS2 | 8660 | insulin receptor substrate 2 (IRS2), mRNA. | -2.14 | -4.28 | 0 |
| HBA1 | 3039 | hemoglobin, alpha 1 (HBA1), mRNA. | -2.15 | -1.43 | 0.006 |
| CFH | 3075 | complement factor H (CFH), transcript variant 1, mRNA./// complement factor H (CFH), transcript variant 2, mRNA. | -2.15 | -5.19 | 0 |
| DDIT4 | 54541 | DNA-damage-inducible transcript 4 (DDIT4), mRNA. | -2.15 | -3.78 | 0 |
| TSPAN6 | 7105 | tetraspanin 6 (TSPAN6), mRNA. | -2.15 | -5.46 | 0 |
| SLCO4C1 | 353189 | solute carrier organic anion transporter family, member 4C1 (SLCO4C1), mRNA. | -2.16 | -4.18 | 0 |
| CMTM4 | 146223 | CKLF-like MARVEL transmembrane domain containing 4 (CMTM4), transcript variant 2, mRNA. | -2.16 | -9.97 | 0 |
| FAM3C | 10447 | family with sequence similarity 3, member C (FAM3C), transcript variant 2, mRNA. | -2.16 | -5.42 | 0 |
| NFIL3 | 4783 | nuclear factor, interleukin 3 regulated (NFIL3), mRNA. | -2.16 | -3.88 | 0 |
| RPL7 | 6129 | ribosomal protein L7 (RPL7), mRNA. | -2.17 | -5.27 | 0 |
| NR4A2 | 4929 | nuclear receptor subfamily 4, group A, member 2 (NR4A2), transcript variant 1, mRNA. | -2.17 | -2.16 | 0.003 |
| AGPAT1 | 10554 | 1-acylglycerol-3-phosphate O-acyltransferase 1 (lysophosphatidic acid acyltransferase, alpha) (AGPAT1), transcript variant 1, mRNA. | -2.18 | -4.18 | 0 |
| HS.19339 |  | cDNA clone IMAGE:5263177 | -2.18 | -5.10 | 0 |
| LOC100130892 | 100130892 | PREDICTED: hypothetical protein LOC100130892 (LOC100130892), mRNA. | -2.18 | -5.85 | 0 |
| HS.374278 |  | cDNA FLJ38388 fis, clone FEBRA2004485 | -2.18 | -3.90 | 0 |
| IL1R2 | 7850 | interleukin 1 receptor, type II (IL1R2), transcript variant 2, mRNA. | -2.18 | -4.88 | 0 |
| FCER1A | 2205 | Fc fragment of IgE, high affinity I, receptor for; alpha polypeptide (FCER1A), mRNA. | -2.19 | -3.25 | 0 |
| C17ORF45 | 125144 | chromosome 17 open reading frame 45 (C17orf45), mRNA. | -2.19 | -6.93 | 0 |
| LOC391019 | 391019 | PREDICTED: misc_RNA (LOC391019), miscRNA. | -2.19 | -4.04 | 0 |
| GABARAPL1 | 23710 | GABA(A) receptor-associated protein like 1 (GABARAPL1), mRNA. | -2.19 | -6.73 | 0 |
| NNAT | 4826 | neuronatin (NNAT), transcript variant 2, mRNA. | -2.19 | -3.00 | 0 |
| HS.10862 |  | cDNA: FLJ23313 fis, clone HEP11919 | -2.19 | -5.55 | 0 |
| SOX9 | 6662 | SRY (sex determining region Y)-box 9 (campomelic dysplasia, autosomal sex-reversal) (SOX9), mRNA. | -2.19 | -4.12 | 0 |
| LOC728843 | 728843 | PREDICTED: misc_RNA (LOC728843), miscRNA. | -2.20 | -5.24 | 0 |
| MYOZ1 | 58529 | myozenin 1 (MYOZ1), mRNA. | -2.20 | -2.27 | 0.002 |
| TM7SF2 | 7108 | transmembrane 7 superfamily member 2 (TM7SF2), mRNA. | -2.20 | -3.53 | 0 |
| DDAH1 | 23576 | dimethylarginine dimethylaminohydrolase 1 (DDAH1), mRNA. | -2.20 | -7.87 | 0 |
| CLDN1 | 9076 | claudin 1 (CLDN1), mRNA. | -2.21 | -4.84 | 0 |
| LPHN3 | 23284 | latrophilin 3 (LPHN3), mRNA. | -2.21 | -9.85 | 0 |
| MYL2 | 4633 | myosin, light polypeptide 2, regulatory, cardiac, slow (MYL2), mRNA. | -2.21 | -2.68 | 0.0007 |
| UGT2B7 | 7364 | PREDICTED: UDP glucuronosyltransferase 2 family, polypeptide B7 (UGT2B7), mRNA.///PREDICTED: UDP glucuronosyltransferase 2 family, polypeptide B7, transcript variant 3 (UGT2B7), mRNA. | -2.22 | -4.22 | 0 |
| RTN4 | 57142 | reticulon 4 (RTN4), transcript variant 3, mRNA./// reticulon 4 (RTN4), transcript variant 1, mRNA. | -2.23 | -5.53 | 0 |
| GCSH | 2653 | glycine cleavage system protein H (aminomethyl carrier) (GCSH), mRNA. | -2.24 | -3.94 | 0 |
| CNTFR | 1271 | ciliary neurotrophic factor receptor (CNTFR), transcript variant 2, mRNA. | -2.24 | -6.53 | 0 |
| HS.88156 |  | 602507996F1 NIH_MGC_79 cDNA clone IMAGE:4605190 5, mRNA sequence | -2.24 | -4.59 | 0 |
| HDC | 3067 | histidine decarboxylase (HDC), mRNA. | -2.25 | -5.57 | 0 |
| ELOVL4 | 6785 | elongation of very long chain fatty acids (FEN1/Elo2, SUR4/Elo3, yeast)-like 4 (ELOVL4), mRNA. | -2.26 | -5.42 | 0 |
| BCHE | 590 | butyrylcholinesterase (BCHE), mRNA. | -2.26 | -6.84 | 0 |
| WFDC3 | 140686 | WAP four-disulfide core domain 3 (WFDC3), mRNA./// WAP four-disulfide core domain 3 (WFDC3), transcript variant 2, mRNA. | -2.26 | -5.41 | 0 |
| NEB | 4703 | nebulin (NEB), mRNA. | -2.26 | -2.25 | 0.002 |
| DPT | 1805 | dermatopontin (DPT), mRNA. | -2.27 | -3.62 | 0 |
| CTGF | 1490 | connective tissue growth factor (CTGF), mRNA. | -2.27 | -2.70 | 0.0007 |
| UGT2B17 | 7367 | UDP glucuronosyltransferase 2 family, polypeptide B17 (UGT2B17), mRNA. | -2.27 | -4.30 | 0 |
| BCAP29 | 55973 | B-cell receptor-associated protein 29 (BCAP29), transcript variant 4, mRNA./// B-cell receptor-associated protein 29 (BCAP29), transcript variant 2, mRNA./// B-cell receptor-associated protein 29 (BCAP29), transcript variant 3, mRNA./// B-cell receptor-associated protein 29 (BCAP29), transcript variant 1, mRNA. | -2.28 | -4.06 | 0 |
| PROL1 | 58503 | proline rich, lacrimal 1 (PROL1), mRNA. | -2.29 | -1.62 | 0.005 |
| NSDHL | 50814 | NAD(P) dependent steroid dehydrogenase-like (NSDHL), mRNA. | -2.29 | -4.12 | 0 |
| UNG | 7374 | uracil-DNA glycosylase (UNG), nuclear gene encoding mitochondrial protein, transcript variant 1, mRNA. | -2.29 | -5.34 | 0 |
| STC1 | 6781 | stanniocalcin 1 (STC1), mRNA. | -2.29 | -3.60 | 0 |
| IL17D | 53342 | interleukin 17D (IL17D), mRNA. | -2.29 | -5.53 | 0 |
| ALDH1L1 | 10840 | aldehyde dehydrogenase 1 family, member L1 (ALDH1L1), mRNA. | -2.30 | -3.71 | 0 |
| MT1X | 4501 | metallothionein 1X (MT1X), mRNA. | -2.30 | -3.77 | 0 |
| GSTM3 | 2947 | glutathione S-transferase M3 (brain) (GSTM3), mRNA. | -2.30 | -4.82 | 0 |
| SLC7A2 | 6542 | solute carrier family 7 (cationic amino acid transporter, y+ system), member 2 (SLC7A2), transcript variant 2, mRNA. | -2.31 | -8.04 | 0 |
| FRZB | 2487 | frizzled-related protein (FRZB), mRNA. | -2.31 | -5.29 | 0 |
| C21ORF63 | 59271 | chromosome 21 open reading frame 63 (C21orf63), mRNA. | -2.31 | -5.37 | 0 |
| LOC100134504 | 100134504 | PREDICTED: hypothetical protein LOC100134504 (LOC100134504), mRNA. | -2.32 | -5.38 | 0 |
| LOC651149 | 651149 | PREDICTED: similar to 60S ribosomal protein L3 (L4) (LOC651149), mRNA. | -2.32 | -6.79 | 0 |
| PNLIPRP3 | 119548 | pancreatic lipase-related protein 3 (PNLIPRP3), mRNA. | -2.33 | -2.35 | 0.001 |
| LOC642934 | 642934 | PREDICTED: hypothetical LOC642934 (LOC642934), mRNA. | -2.34 | -5.78 | 0 |
| BRP44 | 25874 | brain protein 44 (BRP44), transcript variant 2, mRNA. | -2.35 | -4.16 | 0 |
| KRT27 | 342574 | keratin 27 (KRT27), mRNA. | -2.35 | -1.38 | 0.006 |
| REEP6 | 92840 | receptor accessory protein 6 (REEP6), mRNA. | -2.36 | -5.07 | 0 |
| MVD | 4597 | mevalonate (diphospho) decarboxylase (MVD), mRNA. | -2.36 | -4.64 | 0 |
| ERRFI1 | 54206 | ERBB receptor feedback inhibitor 1 (ERRFI1), mRNA. | -2.37 | -3.33 | 0 |
| AACS | 65985 | acetoacetyl-CoA synthetase (AACS), mRNA. | -2.37 | -4.72 | 0 |
| PRDX2 | 7001 | peroxiredoxin 2 (PRDX2), nuclear gene encoding mitochondrial protein, transcript variant 3, mRNA. | -2.38 | -4.63 | 0 |
| TNMD | 64102 | tenomodulin (TNMD), mRNA. | -2.39 | -5.01 | 0 |
| MAOA | 4128 | monoamine oxidase A (MAOA), nuclear gene encoding mitochondrial protein, mRNA. | -2.39 | -5.24 | 0 |
| CHI3L2 | 1117 | chitinase 3-like 2 (CHI3L2), transcript variant 1, mRNA. | -2.39 | -3.47 | 0 |
| PADI4 | 23569 | peptidyl arginine deiminase, type IV (PADI4), mRNA. | -2.39 | -4.79 | 0 |
| GGT6 | 124975 | gamma-glutamyltransferase 6 homolog (rat) (GGT6), mRNA. | -2.40 | -6.28 | 0 |
| DGAT2 | 84649 | diacylglycerol O-acyltransferase homolog 2 (mouse) (DGAT2), mRNA. | -2.40 | -5.25 | 0 |
| PMVK | 10654 | phosphomevalonate kinase (PMVK), mRNA. | -2.40 | -4.23 | 0 |
| CMYA5 | 202333 | cardiomyopathy associated 5 (CMYA5), mRNA. | -2.40 | -3.93 | 0 |
| ADAMTS1 | 9510 | ADAM metallopeptidase with thrombospondin type 1 motif, 1 (ADAMTS1), mRNA. | -2.40 | -3.68 | 0 |
| ANGPTL4 | 51129 | angiopoietin-like 4 (ANGPTL4), transcript variant 3, mRNA./// angiopoietin-like 4 (ANGPTL4), transcript variant 1, mRNA. | -2.41 | -4.59 | 0 |
| HADH | 3033 | hydroxyacyl-Coenzyme A dehydrogenase (HADH), nuclear gene encoding mitochondrial protein, mRNA. | -2.41 | -5.69 | 0 |
| IL20RA | 53832 | interleukin 20 receptor, alpha (IL20RA), mRNA. | -2.41 | -6.47 | 0 |
| EFNB2 | 1948 | ephrin-B2 (EFNB2), mRNA. | -2.41 | -7.53 | 0 |
| HIBCH | 26275 | 3-hydroxyisobutyryl-Coenzyme A hydrolase (HIBCH), nuclear gene encoding mitochondrial protein, transcript variant 2, mRNA. | -2.43 | -4.08 | 0 |
| UAP1 | 6675 | UDP-N-acteylglucosamine pyrophosphorylase 1 (UAP1), mRNA. | -2.44 | -4.21 | 0 |
| UPB1 | 51733 | ureidopropionase, beta (UPB1), mRNA. | -2.45 | -3.55 | 0 |
| MIR1974 | 100302207 | microRNA 1974 (MIR1974), microRNA. | -2.46 | -2.91 | 0.0007 |
| HSD17B2 | 3294 | hydroxysteroid (17-beta) dehydrogenase 2 (HSD17B2), mRNA. | -2.47 | -3.53 | 0 |
| IER3 | 8870 | immediate early response 3 (IER3), mRNA. | -2.47 | -5.01 | 0 |
| ALCAM | 214 | activated leukocyte cell adhesion molecule (ALCAM), mRNA. | -2.48 | -6.15 | 0 |
| AQP9 | 366 | aquaporin 9 (AQP9), mRNA. | -2.48 | -4.89 | 0 |
| C5ORF27///FIS | 202299 | PREDICTED: misc_RNA (C5orf27), miscRNA./// FIS (FIS), mRNA. | -2.48 | -3.94 | 0 |
| PANK1 | 53354 | pantothenate kinase 1 (PANK1), transcript variant gamma, mRNA./// pantothenate kinase 1 (PANK1), transcript variant beta, mRNA. | -2.48 | -5.00 | 0 |
| LOC100134361 | 100134361 | PREDICTED: similar to hCG1811002 (LOC100134361), mRNA. | -2.48 | -8.14 | 0 |
| LOC100129781 | 100129781 | PREDICTED: hypothetical protein LOC100129781 (LOC100129781), mRNA. | -2.49 | -3.50 | 0 |
| ACOT1 | 641371 | acyl-CoA thioesterase 1 (ACOT1), mRNA. | -2.49 | -3.59 | 0 |
| MUC1 | 4582 | mucin 1, cell surface associated (MUC1), transcript variant 5, mRNA./// mucin 1, cell surface associated (MUC1), transcript variant 1, mRNA./// mucin 1, cell surface associated (MUC1), transcript variant 6, mRNA. | -2.51 | -3.05 | 0 |
| IDI1 | 3422 | isopentenyl-diphosphate delta isomerase 1 (IDI1), mRNA. | -2.53 | -4.56 | 0 |
| TLCD1 | 116238 | TLC domain containing 1 (TLCD1), mRNA. | -2.54 | -4.22 | 0 |
| ACP6 | 51205 | acid phosphatase 6, lysophosphatidic (ACP6), mRNA. | -2.56 | -4.73 | 0 |
| PPARG | 5468 | peroxisome proliferator-activated receptor gamma (PPARG), transcript variant 2, mRNA./// peroxisome proliferator-activated receptor gamma (PPARG), transcript variant 1, mRNA. | -2.56 | -4.68 | 0 |
| PEX11A | 8800 | peroxisomal biogenesis factor 11 alpha (PEX11A), mRNA. | -2.57 | -4.27 | 0 |
| MT1M | 4499 | metallothionein 1M (MT1M), mRNA. | -2.58 | -3.42 | 0 |
| HMGCR | 3156 | 3-hydroxy-3-methylglutaryl-Coenzyme A reductase (HMGCR), mRNA. | -2.59 | -5.42 | 0 |
| LOC730005 | 730005 | PREDICTED: similar to SEC14p-like protein TAP3 (LOC730005), mRNA. | -2.59 | -4.25 | 0 |
| FNDC4 | 64838 | fibronectin type III domain containing 4 (FNDC4), mRNA. | -2.59 | -8.26 | 0 |
| ECHDC3 | 79746 | enoyl Coenzyme A hydratase domain containing 3 (ECHDC3), mRNA. | -2.59 | -5.53 | 0 |
| SRD5A1 | 6715 | steroid-5-alpha-reductase, alpha polypeptide 1 (3-oxo-5 alpha-steroid delta 4-dehydrogenase alpha 1) (SRD5A1), mRNA. | -2.60 | -4.14 | 0 |
| IL6 | 3569 | interleukin 6 (interferon, beta 2) (IL6), mRNA. | -2.61 | -2.42 | 0.001 |
| GK5 | 256356 | glycerol kinase 5 (putative) (GK5), mRNA. | -2.61 | -4.03 | 0 |
| ME1 | 4199 | malic enzyme 1, NADP(+)-dependent, cytosolic (ME1), mRNA. | -2.62 | -4.62 | 0 |
| HACL1 | 26061 | 2-hydroxyacyl-CoA lyase 1 (HACL1), mRNA. | -2.62 | -4.32 | 0 |
| PGRMC1 | 10857 | progesterone receptor membrane component 1 (PGRMC1), mRNA. | -2.63 | -5.52 | 0 |
| ENO3 | 2027 | enolase 3 (beta, muscle) (ENO3), transcript variant 1, mRNA. | -2.63 | -2.39 | 0.001 |
| SLC25A18 | 83733 | solute carrier family 25 (mitochondrial carrier), member 18 (SLC25A18), nuclear gene encoding mitochondrial protein, mRNA. | -2.63 | -4.18 | 0 |
| MYOT | 9499 | myotilin (MYOT), mRNA. | -2.65 | -2.96 | 0 |
| FBP1 | 2203 | fructose-1,6-bisphosphatase 1 (FBP1), mRNA. | -2.67 | -3.54 | 0 |
| EGR1 | 1958 | early growth response 1 (EGR1), mRNA. | -2.67 | -3.34 | 0 |
| GNPAT | 8443 | glyceronephosphate O-acyltransferase (GNPAT), mRNA. | -2.67 | -4.54 | 0 |
| FABP9 | 646480 | fatty acid binding protein 9, testis (FABP9), mRNA. | -2.68 | -1.69 | 0.005 |
| ACADM | 34 | acyl-Coenzyme A dehydrogenase, C-4 to C-12 straight chain (ACADM), nuclear gene encoding mitochondrial protein, mRNA. | -2.68 | -3.45 | 0 |
| DIO2 | 1734 | deiodinase, iodothyronine, type II (DIO2), transcript variant 1, mRNA./// deiodinase, iodothyronine, type II (DIO2), transcript variant 3, mRNA. | -2.68 | -4.97 | 0 |
| C5ORF4 | 10826 | chromosome 5 open reading frame 4 (C5orf4), mRNA. | -2.70 | -4.82 | 0 |
| HBEGF | 1839 | heparin-binding EGF-like growth factor (HBEGF), mRNA. | -2.71 | -3.10 | 0 |
| RARRES1 | 5918 | retinoic acid receptor responder (tazarotene induced) 1 (RARRES1), transcript variant 2, mRNA./// retinoic acid receptor responder (tazarotene induced) 1 (RARRES1), transcript variant 1, mRNA. | -2.72 | -4.21 | 0 |
| C7ORF41 | 222166 | chromosome 7 open reading frame 41 (C7orf41), mRNA. | -2.74 | -8.97 | 0 |
| FOXC1 | 2296 | forkhead box C1 (FOXC1), mRNA. | -2.76 | -4.19 | 0 |
| SGPP2 | 130367 | sphingosine-1-phosphate phosphotase 2 (SGPP2), mRNA.///PREDICTED: sphingosine-1-phosphate phosphotase 2 (SGPP2), mRNA. | -2.77 | -6.07 | 0 |
| KRT15 | 3866 | keratin 15 (KRT15), mRNA. | -2.79 | -2.96 | 0 |
| FDPS | 2224 | farnesyl diphosphate synthase (farnesyl pyrophosphate synthetase, dimethylallyltranstransferase, geranyltranstransferase) (FDPS), mRNA. | -2.81 | -4.93 | 0 |
| ACAA2 | 10449 | acetyl-Coenzyme A acyltransferase 2 (mitochondrial 3-oxoacyl-Coenzyme A thiolase) (ACAA2), nuclear gene encoding mitochondrial protein, mRNA. | -2.81 | -4.29 | 0 |
| CILP | 8483 | cartilage intermediate layer protein, nucleotide pyrophosphohydrolase (CILP), mRNA. | -2.81 | -3.56 | 0 |
| FOSB | 2354 | FBJ murine osteosarcoma viral oncogene homolog B (FOSB), mRNA. | -2.82 | -1.81 | 0.003 |
| ACSS2 | 55902 | acyl-CoA synthetase short-chain family member 2 (ACSS2), transcript variant 2, mRNA./// acyl-CoA synthetase short-chain family member 2 (ACSS2), transcript variant 1, mRNA. | -2.82 | -4.60 | 0 |
| C10ORF57 | 80195 | chromosome 10 open reading frame 57 (C10orf57), mRNA. | -2.83 | -5.19 | 0 |
| EBP | 10682 | emopamil binding protein (sterol isomerase) (EBP), mRNA. | -2.85 | -4.88 | 0 |
| LOC643031 | 643031 | PREDICTED: similar to NADH dehydrogenase subunit 5 (LOC643031), mRNA. | -2.86 | -10.17 | 0 |
| PXMP2 | 5827 | peroxisomal membrane protein 2, 22kDa (PXMP2), mRNA. | -2.87 | -4.72 | 0 |
| SERPINA3 | 12 | serpin peptidase inhibitor, clade A (alpha-1 antiproteinase, antitrypsin), member 3 (SERPINA3), mRNA. | -2.87 | -3.08 | 0 |
| ASIP | 434 | agouti signaling protein, nonagouti homolog (mouse) (ASIP), mRNA. | -2.87 | -6.54 | 0 |
| HS.534061 |  | full-length cDNA clone XCL0BB001ZD04 of Neuroblastoma of (human) | -2.92 | -5.13 | 0 |
| KRT25 | 147183 | keratin 25 (KRT25), mRNA. | -2.92 | -1.82 | 0.003 |
| ISOC1 | 51015 | isochorismatase domain containing 1 (ISOC1), mRNA. | -2.97 | -7.45 | 0 |
| BRI3BP | 140707 | PREDICTED: BRI3 binding protein (BRI3BP), mRNA./// BRI3 binding protein (BRI3BP), mRNA. | -3.00 | -5.33 | 0 |
| ACSL1 | 2180 | acyl-CoA synthetase long-chain family member 1 (ACSL1), mRNA. | -3.00 | -5.09 | 0 |
| HMGCS2 | 3158 | 3-hydroxy-3-methylglutaryl-Coenzyme A synthase 2 (mitochondrial) (HMGCS2), nuclear gene encoding mitochondrial protein, mRNA. | -3.02 | -6.99 | 0 |
| DHCR7 | 1717 | 7-dehydrocholesterol reductase (DHCR7), mRNA./// 7-dehydrocholesterol reductase (DHCR7), transcript variant 1, mRNA. | -3.03 | -4.91 | 0 |
| TCHH | 7062 | trichohyalin (TCHH), mRNA. | -3.03 | -2.11 | 0.003 |
| GATM | 2628 | glycine amidinotransferase (L-arginine:glycine amidinotransferase) (GATM), nuclear gene encoding mitochondrial protein, mRNA. | -3.04 | -6.75 | 0 |
| ABHD5 | 51099 | abhydrolase domain containing 5 (ABHD5), mRNA. | -3.04 | -4.97 | 0 |
| FOS | 2353 | v-fos FBJ murine osteosarcoma viral oncogene homolog (FOS), mRNA. | -3.04 | -2.38 | 0.001 |
| HLA-DQB2 | 3120 | major histocompatibility complex, class II, DQ beta 2 (HLA-DQB2), mRNA. | -3.10 | -5.89 | 0 |
| HS.388347 |  | mRNA; cDNA DKFZp686J0156 (from clone DKFZp686J0156) | -3.12 | -8.24 | 0 |
| IRX6 | 79190 | iroquois homeobox 6 (IRX6), mRNA. | -3.13 | -5.59 | 0 |
| SOAT1 | 6646 | sterol O-acyltransferase 1 (SOAT1), transcript variant 688113, mRNA. | -3.15 | -4.86 | 0 |
| FCGBP | 8857 | Fc fragment of IgG binding protein (FCGBP), mRNA.///PREDICTED: Fc fragment of IgG binding protein (FCGBP), mRNA. | -3.15 | -4.69 | 0 |
| ANKRD1 | 27063 | ankyrin repeat domain 1 (cardiac muscle) (ANKRD1), mRNA. | -3.16 | -2.88 | 0.0007 |
| CST6 | 1474 | PREDICTED: cystatin E/M (CST6), mRNA./// cystatin E/M (CST6), mRNA. | -3.17 | -3.60 | 0 |
| DHCR24 | 1718 | 24-dehydrocholesterol reductase (DHCR24), mRNA. | -3.17 | -6.36 | 0 |
| SPRR4 | 163778 | small proline-rich protein 4 (SPRR4), mRNA. | -3.19 | -3.04 | 0 |
| PDK4 | 5166 | pyruvate dehydrogenase kinase, isozyme 4 (PDK4), mRNA. | -3.20 | -4.64 | 0 |
| CYB5A | 1528 | cytochrome b5 type A (microsomal) (CYB5A), transcript variant 2, mRNA. | -3.27 | -4.94 | 0 |
| CD1A | 909 | CD1a molecule (CD1A), mRNA. | -3.28 | -6.99 | 0 |
| THBS4 | 7060 | thrombospondin 4 (THBS4), mRNA. | -3.28 | -3.27 | 0 |
| TNNC2 | 7125 | troponin C type 2 (fast) (TNNC2), mRNA. | -3.30 | -3.08 | 0 |
| DCT | 1638 | dopachrome tautomerase (dopachrome delta-isomerase, tyrosine-related protein 2) (DCT), mRNA. | -3.34 | -5.21 | 0 |
| ELOVL5 | 60481 | ELOVL family member 5, elongation of long chain fatty acids (FEN1/Elo2, SUR4/Elo3-like, yeast) (ELOVL5), mRNA. | -3.34 | -4.85 | 0 |
| MYBPC1 | 4604 | myosin binding protein C, slow type (MYBPC1), transcript variant 2, mRNA./// myosin binding protein C, slow type (MYBPC1), transcript variant 4, mRNA. | -3.35 | -3.28 | 0 |
| SCGB2A1 | 4246 | secretoglobin, family 2A, member 1 (SCGB2A1), mRNA. | -3.35 | -2.84 | 0.0007 |
| PSAPL1 | 768239 | prosaposin-like 1 (PSAPL1), mRNA. | -3.38 | -6.16 | 0 |
| BCAT2 | 587 | branched chain aminotransferase 2, mitochondrial (BCAT2), nuclear gene encoding mitochondrial protein, mRNA. | -3.42 | -4.77 | 0 |
| ACSM3 | 6296 | acyl-CoA synthetase medium-chain family member 3 (ACSM3), transcript variant 1, mRNA. | -3.46 | -4.34 | 0 |
| RPS23 | 6228 | ribosomal protein S23 (RPS23), mRNA. | -3.52 | -4.71 | 0 |
| C20ORF3 | 57136 | chromosome 20 open reading frame 3 (C20orf3), mRNA. | -3.54 | -6.11 | 0 |
| PLIN2 | 123 | perilipin 2 (PLIN2), mRNA. | -3.58 | -5.26 | 0 |
| FA2H | 79152 | fatty acid 2-hydroxylase (FA2H), mRNA. | -3.58 | -7.80 | 0 |
| ACOX2 | 8309 | acyl-Coenzyme A oxidase 2, branched chain (ACOX2), mRNA. | -3.61 | -4.92 | 0 |
| TNNC1 | 7134 | troponin C type 1 (slow) (TNNC1), mRNA. | -3.61 | -3.46 | 0 |
| IL8 | 3576 | interleukin 8 (IL8), mRNA. | -3.66 | -2.68 | 0.0007 |
| KBTBD10 | 10324 | kelch repeat and BTB (POZ) domain containing 10 (KBTBD10), mRNA. | -3.73 | -2.90 | 0.0007 |
| TMEM97 | 27346 | transmembrane protein 97 (TMEM97), mRNA. | -3.74 | -5.85 | 0 |
| MGST1 | 4257 | microsomal glutathione S-transferase 1 (MGST1), transcript variant 1a, mRNA./// microsomal glutathione S-transferase 1 (MGST1), transcript variant 1b, mRNA./// microsomal glutathione S-transferase 1 (MGST1), transcript variant 1c, mRNA. | -3.79 | -4.66 | 0 |
| UBIAD1 | 29914 | UbiA prenyltransferase domain containing 1 (UBIAD1), mRNA. | -3.80 | -5.23 | 0 |
| SC5DL | 6309 | sterol-C5-desaturase (ERG3 delta-5-desaturase homolog, S. cerevisiae)-like (SC5DL), transcript variant 1, mRNA. | -3.83 | -5.19 | 0 |
| ACAT2 | 39 | acetyl-Coenzyme A acetyltransferase 2 (acetoacetyl Coenzyme A thiolase) (ACAT2), mRNA./// acetyl-Coenzyme A acetyltransferase 2 (ACAT2), mRNA. | -3.84 | -5.17 | 0 |
| CRAT | 1384 | carnitine acetyltransferase (CRAT), nuclear gene encoding mitochondrial protein, transcript variant 1, mRNA. | -3.86 | -4.84 | 0 |
| ERV3 | 2086 | endogenous retroviral sequence 3 (includes zinc finger protein H-plk/HPF9) (ERV3), mRNA. | -3.86 | -4.72 | 0 |
| GPT | 2875 | glutamic-pyruvate transaminase (alanine aminotransferase) (GPT), mRNA. | -3.88 | -7.71 | 0 |
| SC4MOL | 6307 | sterol-C4-methyl oxidase-like (SC4MOL), transcript variant 2, mRNA./// sterol-C4-methyl oxidase-like (SC4MOL), transcript variant 1, mRNA. | -3.90 | -5.16 | 0 |
| C6ORF105 | 84830 | chromosome 6 open reading frame 105 (C6orf105), mRNA. | -4.25 | -4.33 | 0 |
| INSIG1 | 3638 | insulin induced gene 1 (INSIG1), transcript variant 2, mRNA. | -4.31 | -4.88 | 0 |
| HMGCS1 | 3157 | 3-hydroxy-3-methylglutaryl-Coenzyme A synthase 1 (soluble) (HMGCS1), transcript variant 2, mRNA. | -4.37 | -5.54 | 0 |
| PECR | 55825 | peroxisomal trans-2-enoyl-CoA reductase (PECR), mRNA. | -4.39 | -4.13 | 0 |
| SLN | 6588 | sarcolipin (SLN), mRNA. | -4.43 | -3.42 | 0 |
| FASN | 2194 | fatty acid synthase (FASN), mRNA. | -4.43 | -5.40 | 0 |
| CHI3L1 | 1116 | chitinase 3-like 1 (cartilage glycoprotein-39) (CHI3L1), mRNA. | -4.69 | -5.36 | 0 |
| APOC1 | 341 | apolipoprotein C-I (APOC1), mRNA. | -4.70 | -4.66 | 0 |
| ACO1 | 48 | aconitase 1, soluble (ACO1), mRNA. | -4.95 | -6.35 | 0 |
| TMEM91 | 641649 | transmembrane protein 91 (TMEM91), mRNA. | -4.97 | -4.97 | 0 |
| LRG1 | 116844 | leucine-rich alpha-2-glycoprotein 1 (LRG1), mRNA. | -4.98 | -5.85 | 0 |
| ACTA1 | 58 | actin, alpha 1, skeletal muscle (ACTA1), mRNA. | -5.07 | -2.31 | 0.002 |
| C7ORF68 | 29923 | chromosome 7 open reading frame 68 (C7orf68), transcript variant 1, mRNA. | -5.10 | -5.70 | 0 |
| MB | 4151 | myoglobin (MB), transcript variant 1, mRNA. | -5.11 | -3.11 | 0 |
| HSD11B1 | 3290 | hydroxysteroid (11-beta) dehydrogenase 1 (HSD11B1), transcript variant 2, mRNA./// hydroxysteroid (11-beta) dehydrogenase 1 (HSD11B1), transcript variant 1, mRNA. | -5.19 | -5.20 | 0 |
| PDZK1 | 5174 | PDZ domain containing 1 (PDZK1), mRNA. | -5.29 | -5.69 | 0 |
| PON3 | 5446 | paraoxonase 3 (PON3), mRNA. | -5.45 | -5.46 | 0 |
| MOGAT1 | 116255 | monoacylglycerol O-acyltransferase 1 (MOGAT1), mRNA. | -5.47 | -4.89 | 0 |
| FADS2 | 9415 | fatty acid desaturase 2 (FADS2), mRNA. | -5.65 | -4.93 | 0 |
| CIDEA | 1149 | cell death-inducing DFFA-like effector a (CIDEA), transcript variant 2, mRNA./// cell death-inducing DFFA-like effector a (CIDEA), transcript variant 1, mRNA. | -5.72 | -6.36 | 0 |
| TF | 7018 | transferrin (TF), mRNA. | -5.76 | -5.62 | 0 |
| PM20D1///FLJ32569 | 148811 | peptidase M20 domain containing 1 (PM20D1), mRNA.///PREDICTED: hypothetical protein FLJ32569 (FLJ32569), mRNA. | -6.22 | -4.48 | 0 |
| FAR2 | 55711 | fatty acyl CoA reductase 2 (FAR2), mRNA. | -6.32 | -5.09 | 0 |
| KRT79 | 338785 | keratin 79 (KRT79), mRNA. | -6.64 | -6.70 | 0 |
| GLDC | 2731 | glycine dehydrogenase (decarboxylating) (GLDC), mRNA. | -6.66 | -5.77 | 0 |
| FADS1 | 3992 | fatty acid desaturase 1 (FADS1), mRNA. | -6.70 | -4.05 | 0 |
| THRSP | 7069 | thyroid hormone responsive (SPOT14 homolog, rat) (THRSP), mRNA. | -7.21 | -4.39 | 0 |
| FABP7 | 2173 | fatty acid binding protein 7, brain (FABP7), mRNA. | -7.79 | -5.87 | 0 |
| ALOX15B | 247 | arachidonate 15-lipoxygenase, type B (ALOX15B), transcript variant b, mRNA./// arachidonate 15-lipoxygenase, type B (ALOX15B), transcript variant d, mRNA. | -7.97 | -4.68 | 0 |
| COCH | 1690 | coagulation factor C homolog, cochlin (Limulus polyphemus) (COCH), mRNA. | -8.42 | -4.34 | 0 |
| HSD3B1 | 3283 | hydroxy-delta-5-steroid dehydrogenase, 3 beta- and steroid delta-isomerase 1 (HSD3B1), mRNA. | -9.49 | -7.14 | 0 |
| ELOVL3 | 83401 | elongation of very long chain fatty acids (FEN1/Elo2, SUR4/Elo3, yeast)-like 3 (ELOVL3), mRNA. | -9.76 | -5.83 | 0 |
| ACSBG1 | 23205 | acyl-CoA synthetase bubblegum family member 1 (ACSBG1), mRNA. | -10.78 | -7.33 | 0 |
| LOC126767 | 126767 | PREDICTED: similar to arylacetamide deacetylase, transcript variant 2 (LOC126767), mRNA. | -11.05 | -5.67 | 0 |
| GAL | 51083 | galanin prepropeptide (GAL), mRNA. | -12.75 | -5.88 | 0 |

Table S3. List of commonly differentially expressed genes in DLE and normal skin, and IFN-γ treated (M1) and IL-4 treated (M2) macrophages.

| Gene Symbol | Gene Title | Fold Change (DLE vs normal) | Differentially expressed in IFN-γ (M1) or IL-4 (M2) treated macrophages? |
| --- | --- | --- | --- |
| CXCL10 | chemokine (C-X-C motif) ligand 10 | 66.66 | M1 |
| ISG15 | ISG15 ubiquitin-like modifier | 57.09 | M1 |
| IFI44L | interferon-induced protein 44-like | 35.74 | M1 |
| MX1 | myxovirus (influenza virus) resistance 1, interferon-inducible protein p78 (mouse) | 26.52 | M1 |
| CXCL9 | chemokine (C-X-C motif) ligand 9 | 23.09 | M1 |
| OAS2 | 2'-5'-oligoadenylate synthetase 2, 69/71kDa | 22.22 | M1 |
| IFIT1 | interferon-induced protein with tetratricopeptide repeats 1 | 20.38 | M1 |
| IFIT2 | interferon-induced protein with tetratricopeptide repeats 2 | 20.09 | M1 |
| EPSTI1 | epithelial stromal interaction 1 (breast) | 19.97 | M1 |
| IFI6 | interferon, alpha-inducible protein 6 | 19.33 | M1 |
| IFI44 | interferon-induced protein 44 | 18.31 | M1 |
| MX2 | myxovirus (influenza virus) resistance 2 (mouse) | 15.99 | M1 |
| RSAD2 | radical S-adenosyl methionine domain containing 2 | 15.87 | M1 |
| IFIT3 | interferon-induced protein with tetratricopeptide repeats 3 | 15.78 | M1 |
| BST2 | bone marrow stromal cell antigen 2 | 14.38 | M1 |
| IFI27 | interferon, alpha-inducible protein 27 | 13.23 | M1 |
| XAF1 | XIAP associated factor 1 | 12.57 | M1 |
| OAS3 | 2'-5'-oligoadenylate synthetase 3, 100kDa | 11.16 | M1 |
| STAT1 | signal transducer and activator of transcription 1, 91kDa | 10.33 | M1 |
| IDO1 | indoleamine 2,3-dioxygenase 1 | 9.44 | M1 |
| ISG20 | interferon stimulated exonuclease gene 20kDa | 9.39 | M1 |
| SAMD9L | sterile alpha motif domain containing 9-like | 9.27 | M1 |
| GBP4 | guanylate binding protein 4 | 8.91 | M1 |
| IRF7 | interferon regulatory factor 7 | 8.53 | M1 |
| IFI35 | interferon-induced protein 35 | 8.09 | M1 |
| GBP5 | guanylate binding protein 5 | 7.90 | M1 |
| OAS1 | 2'-5'-oligoadenylate synthetase 1, 40/46kDa | 7.36 | M1 |
| PARP14 | poly (ADP-ribose) polymerase family, member 14 | 7.12 | M1 |
| GBP1 | guanylate binding protein 1, interferon-inducible | 6.89 | M1 |
| UBE2L6 | ubiquitin-conjugating enzyme E2L 6 | 6.43 | M1 |
| PARP9 | poly (ADP-ribose) polymerase family, member 9 | 6.15 | M1 |
| EIF2AK2 | eukaryotic translation initiation factor 2-alpha kinase 2 | 6.11 | M1 |
| DDX60 | DEAD (Asp-Glu-Ala-Asp) box polypeptide 60 | 6.01 | M1 |
| HLA-F | major histocompatibility complex, class I, F | 5.90 | M1 |
| CFB | complement factor B | 5.70 | M1 |
| SP110 | SP110 nuclear body protein | 5.40 | M1 |
| LGALS3BP | lectin, galactoside-binding, soluble, 3 binding protein | 5.23 | M1 |
| TAP1 | transporter 1, ATP-binding cassette, sub-family B (MDR/TAP) | 5.23 | M1 |
| RTP4 | receptor (chemosensory) transporter protein 4 | 5.23 | M1 |
| STAT2 | signal transducer and activator of transcription 2, 113kDa | 5.04 | M1 |
| LY6E | lymphocyte antigen 6 complex, locus E | 4.76 | M1 |
| WARS | tryptophanyl-tRNA synthetase | 4.47 | M1 |
| UBA7 | ubiquitin-like modifier activating enzyme 7 | 4.42 | M1 |
| TYMP | thymidine phosphorylase | 4.36 | M1 |
| IFITM3 | interferon induced transmembrane protein 3 | 4.36 | M1 |
| DHX58 | DEXH (Asp-Glu-X-His) box polypeptide 58 | 4.29 | M1 |
| PSMB9 | proteasome (prosome, macropain) subunit, beta type, 9 | 4.03 | M1 |
| PARP10 | poly (ADP-ribose) polymerase family, member 10 | 3.88 | M1 |
| TNFSF10 | tumor necrosis factor (ligand) superfamily, member 10 | 3.76 | M1 |
| IRF9 | interferon regulatory factor 9 | 3.64 | M1 |
| ZNFX1 | zinc finger, NFX1-type containing 1 | 3.58 | M1 |
| IFITM2 | interferon induced transmembrane protein 2 | 3.49 | M1 |
| IL4I1 | interleukin 4 induced 1 | 3.44 | M1 |
| IL10RA | interleukin 10 receptor, alpha | 3.42 | M1 |
| FAM26F | family with sequence similarity 26, member F | 3.36 | M1 |
| APOL3 | apolipoprotein L, 3 | 3.34 | M1 |
| CD38 | CD38 molecule | 3.32 | M1 |
| LAP3 | leucine aminopeptidase 3 | 3.31 | M1 |
| IRF1 | interferon regulatory factor 1 | 3.26 | M1 |
| HLA-E | major histocompatibility complex, class I, E | 3.26 | M1 |
| ADAR | adenosine deaminase, RNA-specific | 3.22 | M1 |
| GIMAP8 | GTPase, IMAP family member 8 | 3.16 | M1 |
| GSDMD | gasdermin D | 3.14 | M1 |
| TRIM21 | tripartite motif containing 21 | 3.13 | M1 |
| FBXO6 | F-box protein 6 | 2.97 | M1 |
| PSME2 | proteasome (prosome, macropain) activator subunit 2 (PA28 beta) | 2.84 | M1 |
| PHF11 | PHD finger protein 11 | 2.80 | M1 |
| CASP1 | caspase 1, apoptosis-related cysteine peptidase | 2.77 | M1 |
| SCO2 | SCO2 cytochrome c oxidase assembly protein | 2.74 | M1 |
| TAP2 | transporter 2, ATP-binding cassette, sub-family B (MDR/TAP) | 2.66 | M1 |
| NUB1 | negative regulator of ubiquitin-like proteins 1 | 2.63 | M1 |
| SERPING1 | serpin peptidase inhibitor, clade G (C1 inhibitor), member 1 | 2.59 | M1 |
| MFNG | MFNG O-fucosylpeptide 3-beta-N-acetylglucosaminyltransferase | 2.56 | M2 |
| VAMP5 | vesicle-associated membrane protein 5 | 2.45 | M1 |
| NCF1C | neutrophil cytosolic factor 1C pseudogene | 2.41 | M1 |
| PHF15 | PHD finger protein 15 | 2.41 | M1 |
| PLA1A | phospholipase A1 member A | 2.36 | M1 |
| IL32 | interleukin 32 | 2.30 | M1 |
| GBP2 | guanylate binding protein 2, interferon-inducible | 2.23 | M1 |
| PSME1 | proteasome (prosome, macropain) activator subunit 1 (PA28 alpha) | 2.13 | M1 |
| SOCS1 | suppressor of cytokine signaling 1 | 2.06 | M2 |
| HAPLN3 | hyaluronan and proteoglycan link protein 3 | 2.04 | M1 |
| HIST2H2AC | histone cluster 2, H2ac | 2.03 | M1 |

Table S4. qRT-PCR analysis of selected genes in DLE lesional (N=17) and normal (N=12) skin

| Gene Symbol | Title | Fold change (DLE vs. normal skin) | p-value^***^ |
| --- | --- | --- | --- |
| ISG15^§^ | ISG15 ubiquitin-like modifier | 93.97 | <0.0001 |
| MX1^*,§^ | MX dynamin-like GTPase 1 | 50.01 | <0.0001 |
| GZMB^‡^ | Granzyme B | 19.64 | <0.0001 |
| CD8^‡^ | CD8 antigen | 9.31 | <0.0001 |
| OAS1^§^ | 2’,5’-oligoadenylate synthetase 1 | 8.89 | <0.0001 |
| LY6E^§^ | Lymphocyte antigen 6 complex | 5.13 | <0.0001 |
| CXCR3^†^ | Chemokine (C-X-C motif) receptor 3 | 3.75 | <0.0001 |
| CD86^*,^^ | CD86 molecule | 2.40 | 0.003 |
| CD14^^^ | CD14 molecule | 2.12 | 0.007 |
| FOLR2^**^ | Folate receptor 2 | 1.73 | 0.06 |
| IL-10^**^ | Interleukin-10 | 1.73 | 0.18 |
| IFN-γ^*,†^ | Interferon-γ | 1.72 | 0.10 |
| IL-12^*,†^ | Interleukin-12 | 1.29 | 0.18 |
| CD56^^^ | CD56 molecule, Neural cell adhesion molecule 1 | 0.84 | 0.05 |
| NOS2^*^ | Nitric oxide synthetase 2 | 0.60 | 0.49 |
| CD1A^^^ | CD1a molecule | 0.21 | <0.0001 |

^§^: Type I IFN-related gene

*: M1 macrophage gene

^‡^: Cytotoxic T cell-related gene

^†^: T_H_1-related gene

^^^: Cell surface marker genes for other inflammatory cells (CD86: dendritic cell, CD14: monocyte, CD56: NK cell, CD1a: Langerhans cells)

**: M2 macrophage gene

***: p-values calculated based on Mann-Whitney U test.
